# One-pot preparation of 4-aryl-3-bromocoumarins from 4-aryl-2-propynoic acids with diaryliodonium salts, TBAB, and Na_2_S_2_O_8_

**DOI:** 10.3762/bjoc.14.22

**Published:** 2018-02-05

**Authors:** Teppei Sasaki, Katsuhiko Moriyama, Hideo Togo

**Affiliations:** 1 Department of Chemistry, Graduate School of Science, Chiba University, Yayoi-cho 1-33, Inage-ku, Chiba 263-8522, Japan; 2 Molecular Chirality Research Center, Chiba University, Yayoi-cho 1-33, Inage-ku, Chiba 263-8522, Japan

**Keywords:** 3-aryl-2-propynoic acid, bromo-cyclization, coumarin, diaryliodonium triflate, O-phenylation

## Abstract

Various 4-aryl-3-bromocoumarins were smoothly obtained in moderate yields in one pot by treating 3-aryl-2-propynoic acids with diaryliodonium triflates and K_2_CO_3_ in the presence of CuCl, followed by the reaction with tetrabutylammonium bromide (TBAB) and Na_2_S_2_O_8_. The obtained 3-bromo-4-phenylcoumarin was transformed into 4-phenylcoumarin derivatives bearing C–H, C–S, C–N, and C–C bonds at 3-position.

## Introduction

Coumarin is a benzo-α-pyrone and one of the typical heterocyclic compounds. The importance of coumarins arises from the fact that the coumarin skeleton is present in many natural products extracted from plants [1–3] and some of them show potent pharmacological activities, such as antidepressant [4], antimicrobial [5–6], antioxidants [7–8], anti-inflammatory [9–10], antinociceptive [11], antitumor [1], antiasthmatic [12], and antiviral including anti-HIV [13–14].

Comprehensive synthetic studies of coumarins and their derivatives have been carried out [15–16]. Typically, coumarins are prepared by the acid-catalyzed condensation of 2-alkynoic acids and phenols or the condensation of β-ketoesters and phenols (the Pechmann condensation) [17]. Recent studies for the metal-catalyzed reactions for the synthesis of the coumarin skeleton are as follows: the Yb(OTf)_3_-catalyzed microwave irradiation of phenols and propynoic acids [18], the Pd(OAc)_2_-catalyzed oxidative cyclocarbonylation of 2-vinylphenols at 110 °C [19], the FeCl_3_-catalyzed areneselenyl-cyclization of aryl 2-alkynoates with ArSeSeAr at rt [20], and the Rh-catalyzed annulation of arylthiocarbamates with alkynes/AgOTf/Cu(OAc)_2_ at 120 °C [21]. As examples of the transition-metal-free construction of the coumarin skeleton, the Brønsted acid-catalyzed reaction of phenols and propynoic acids [22] and the (−)-riboflavin-catalyzed photochemical reaction of cinnamic acids [23] were reported recently. Moreover, the use of radical cyclization for the construction of the coumarin skeleton has become widespread. Examples include the radical addition–cyclization reactions of aryl 2-alkynoates with RC(=O)CO_2_H/AgNO_3_(cat.)/K_2_S_2_O_8_ at 60 °C [24], with Cu(OAc)_2_/1-trifluoromethyl-3,3-dimethyl-1,2-benziodoxole (Togni reagent) at 60 °C [25], with R_2_P(=O)H/Ag_2_CO_3_(cat.)/Mg(NO_3_)_2_ at 100 °C [26], with BrCF_2_CO_2_Et/*fac*-Ir(ppy)_3_(cat.) under irradiation at rt [27], with R-CH=O/(*n*-Bu)_4_NBr (TBAB, cat.)/K_2_S_2_O_8_ at 90 °C [28], with ArSO_2_H/Eosin Y(cat.)/*tert*-butyl hydrogen peroxide (TBHP) at rt [29], and with ArSO_2_NHNH_2_/*n*-Bu_4_NI(cat.)/TBHP at 80 °C [30].

In addition, the formation of coumarins via the bromine-radical-mediated reaction of aryl 2-alkynoates with TBAB/K_2_S_2_O_8_ at 90 °C [31], the cyanomethyl-radical-mediated reaction of aryl 2-alkynoates with *tert*-butyl peroxybenzoate (TBPB)/acetonitrile at 130 °C [32], the sunlight-promoted reaction of aryl 2-alkynoates with *N*-iodosuccinimide (NIS) at rt [33], and the visible-light-mediated reaction of aryl 2-alkynoates with *N*-bromosuccinimide (NBS) at rt [34], where those reactions proceed via radical spiro-cyclization and then radical 1,2-carboxyl group migration, were reported.

On the other hand, diaryliodonium salts are very useful for the C-arylation of active CH groups, the O-arylation of OH groups, and the N-arylation of NH groups under metal-free conditions [35–39]. For example, treatment of arenecarboxylic acids and alkanecarboxylic acids with diaryliodonium salts and *t*-BuOK under toluene refluxing conditions provides the corresponding aryl carboxylates in good yields [40–41]. However, the O-arylation of 2-alkynoic acids, which are much more acidic than arenecarboxylic acids and alkanecarboxylic acids, and therefore, the conjugate bases of 2-alkynoic acids are much less nucleophilic than those of arenecarboxylic acids and alkanecarboxylic acids, was not studied. On the other hand, it is known that 4-arylcoumarins have antitumor activity [42]. Therefore, the one-pot preparation of 4-arylcoumarins from 3-aryl-2-alkynoic acids via aryl esters and cyclization is attractive and important.

Here, as part of our ongoing investigation of the synthetic use of diaryliodonium salts for the preparation of heterocyclic compounds [43–46], we would like to report an efficient one-pot preparation of 4-aryl-3-bromocoumarins by treatment of 3-aryl-2-propynoic acids with diaryliodonium triflate in the presence of a base, followed by the reaction with tetrabutylammonium bromide (TBAB) and Na_2_S_2_O_8_ in a mixture of 1,2-dichloroethane and water [31].

## Results and Discussion

First, treatment of 3-phenyl-2-propynoic acid (**1a**, 0.5 mmol) with diphenyliodonium triflate (**A**, 1.0 equiv) in the presence of CuCl (5 mol %) and K_2_CO_3_ (1.0 equiv) in dichloromethane (3.0 mL) at 40 °C based on a previous report [46] gave phenyl 3-phenyl-2-propynoate (**2Aa**) in 46% yield, as shown in Table 1, entry 1. When the amount of the solvent was increased to 7.5 mL under the same conditions, the yield of phenyl ester **2Aa** was increased to 74% (Table 1, entry 2). Under the same conditions, the base was changed to NaH, Cs_2_CO_3_, *t*-BuOK, NaNH_2_, and K_3_PO_4_ instead of K_2_CO_3_. However, the yield of phenyl ester **2Aa** was moderate to low (Table 1, entries 3–7). When the amount of K_2_CO_3_ was reduced to 0.5 equiv under the same conditions as those in entry 2, the yield of phenyl ester **2Aa** was increased to 80% (Table 1, entry 8). When CuCl was changed to CuI and CuBr, the difference of the yield of phenyl ester **2Aa** was small, but CuCl gave the highest yield (Table 1, entries 8–10). Then, the reaction temperature was changed to 0 °C, rt, 50 °C, and 60 °C under the same conditions as those in entry 8, and phenyl ester **2Aa** was obtained in 83% yield at 50 °C (Table 1, entries 11–14). On the other hand, when the present reaction was carried out without CuCl under the same conditions, phenyl ester **2Aa** was not obtained at all (Table 1, entry 15).

**Table 1:** O-Phenylation of 3-phenyl-2-propynoic acid (**1a**) with diphenyliodonium triflate (**A**).

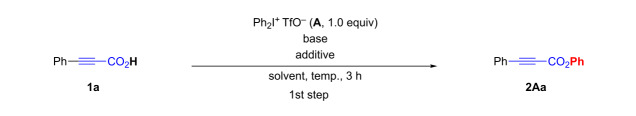					

entry	base	solvent (mL)	additive (mol %)	temp. (°C)	yield (%)

1	K_2_CO_3_ (1.0)	CH_2_Cl_2_ (3.0)	CuCl (5)	40	46
2	K_2_CO_3_ (1.0)	CH_2_Cl_2_ (7.5)	CuCl (5)	40	74
3	NaH (1.0)	CH_2_Cl_2_ (7.5)	CuCl (5)	40	24
4	Cs_2_CO_3_ (0.5)	CH_2_Cl_2_ (7.5)	CuCl (5)	40	17
5	*t*-BuOK (1.0)	CH_2_Cl_2_ (7.5)	CuCl (5)	40	48
6	NaNH_2_ (1.0)	CH_2_Cl_2_ (7.5)	CuCl (5)	40	9
7	K_3_PO_4_ (1.0)	CH_2_Cl_2_ (7.5)	CuCl (5)	40	30
8	K_2_CO_3_ (0.5)	CH_2_Cl_2_ (7.5)	CuCl (5)	40	80
9	K_2_CO_3_ (0.5)	CH_2_Cl_2_ (7.5)	CuI (5)	40	78
10	K_2_CO_3_ (0.5)	CH_2_Cl_2_ (7.5)	CuBr (5)	40	77
11	K_2_CO_3_ (0.5)	CH_2_Cl_2_ (7.5)	CuCl (5)	0	11
12	K_2_CO_3_ (0.5)	CH_2_Cl_2_ (7.5)	CuCl (5)	rt	71
**13**	**K_2_CO_3_ (0.5)**	**DCE (7.5)**	**CuCl (5)**	**50**	**83**
14	K_2_CO_3_ (0.5)	DCE (7.5)	CuCl (5)	60	75
15	K_2_CO_3_ (0.5)	DCE (7.5)	–	50	0

Then, the iodocyclization of phenyl ester **2Aa** to 3-iodo-4-phenylcoumarin (**3Aa’**) with *N*-iodosuccinimide (NIS, 2.0 equiv)/BF_3_·Et_2_O (2.0 or 1.1 equiv) was studied based on the previous reports [45–46], as shown in Table 2. However, 3-iodo-4-phenylcoumarin (**3Aa’**) was obtained in low to moderate yields (Table 2, entries 1 and 2). To improve the yield of 3-halo-4-phenylcoumarins **3Aa** or **3Aa’**, the halocyclization of **2Aa** with *N*-bromosuccinimide (NBS, 2.0 equiv)/BF_3_·Et_2_O (1.1 equiv), with 1,3-diiodo-5,5-dimethylhydantoin (DIH, 2.0 equiv)/BF_3_·Et_2_O (1.1 equiv), and with 1,3-dibromo-5,5-dimethylhydantoin (DBH, 2.0 equiv)/BF_3_·Et_2_O (1.1 equiv) was carried out to form 3-bromo-4-phenylcoumarin (**3Aa**), 3-iodo-4-phenylcoumarin (**3Aa’**), and 3-bromo-4-phenylcoumarin (**3Aa**) in 28, 49 and 46% yields, respectively (Table 2, entries 3–5). The treatment of phenyl ester **2Aa** with molecular iodine (2.0 equiv)/K_2_CO_3_ (2.0 equiv) did not generate 3-iodo-4-phenylcoumarin (**3Aa’**) at all (Table 2, entry 6). Thus, the iodonium-based or bromonium-based electrophilic cyclization of phenyl 3-phenyl-2-propynoate (**2Aa**) does not proceed efficiently. Then, the bromo-radical-based cyclization of phenyl 3-phenyl-2-propynoate (**2Aa**) with tetrabutylammonium bromide (TBAB, 2.0 equiv)/Na_2_S_2_O_8_ (1.5 equiv) [31] in a mixture of 1,2-dichloroethane (DCE) and water at 90 °C was carried out to give 3-bromo-4-phenylcoumarin (**3Aa**) in 68% yield (Table 2, entry 7). When the iodocyclization of phenyl ester **2Aa** with tetrabutylammonium iodide (TBAI, 2.0 equiv)/Na_2_S_2_O_8_ (1.5 equiv) was carried out, the yield of iodocyclization product **3Aa’** was decreased to 45% (Table 2, entry 8). When the bromocyclization of phenyl ester **2Aa** with TBAB (2.0 equiv)/Na_2_S_2_O_8_ (1.0 equiv) in a mixture of DCE and water at 90 °C was carried out, 3-bromo-4-phenylcoumarin (**3Aa**) was obtained in 79% yield (Table 2, entry 9). When Na_2_S_2_O_8_ was increased to 2.0 equivalents or TBAB was increased to 2.5 equivalents under the same conditions, the yields of 3-bromo-4-phenylcoumarin (**3Aa**) were decreased to 51 and 69%, respectively (Table 2, entries 10 and 11). Moreover, when Na_2_S_2_O_8_ was changed to K_2_S_2_O_8_, (NH_4_)_2_S_2_O_8_, and Oxone^®^ (2KHSO_5_·KHSO_4_·K_2_SO_4_), the yields of 3-bromo-4-phenylcoumarin (**3Aa**) were decreased to 71, 69 and 37%, respectively (Table 2, entries 12–14). Thus, it was confirmed that the treatment of phenyl ester **2Aa** with TBAB (2.0 equiv)/Na_2_S_2_O_8_ (1.0 equiv) in a mixture of DCE and water at 90 °C for 19 h was the most efficient, giving 3-bromo-4-phenylcoumarin (**3Aa**) in good yield (Table 2, entry 9).

**Table 2:** Halocyclization of phenyl 3-phenyl-2-propynoate (**2Aa**) to 3-halo-4-phenylcoumarins **3Aa** and **3Aa’**.

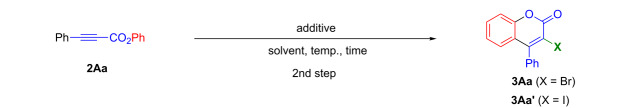					

entry	additive (equiv)	solvent (mL)	temp. (°C)	time (h)	yield (%)

1	NIS (2.0), BF_3_·Et_2_O (2.0)	CH_2_Cl_2_ (3.0)	40	1	36 (**3Aa’**)
2	NIS (2.0), BF_3_·Et_2_O (1.1)	CH_2_Cl_2_ (3.0)	40	1	45 (**3Aa’**)
3	NBS (2.0), BF_3_·Et_2_O (1.1)	CH_2_Cl_2_ (3.0)	40	1	28 (**3Aa**)
4	DIH (2.0), BF_3_·Et_2_O (1.1)	CH_2_Cl_2_ (3.0)	40	1	49 (**3Aa’**)
5	DBH (2.0), BF_3_·Et_2_O (1.1)	CH_2_Cl_2_ (3.0)	40	1	46 (**3Aa**)
6	I_2_ (2.0), K_2_CO_3_ (2.0)	CH_3_CN (3.0)	40	1	0
7	TBAB (2.0), Na_2_S_2_O_8_ (1.5)	DCE:H_2_O (1:1, 5.0)	90	19	68 (**3Aa**)
8	TBAI (2.0), Na_2_S_2_O_8_ (1.5)	DCE:H_2_O (1:1, 5.0)	90	19	45 (**3Aa’**)
**9**	**TBAB (2.0), Na_2_S_2_O_8_ (1.0)**	**DCE:H_2_O (1:1, 5.0)**	**90**	**19**	**79** (**3Aa**)
10	TBAB (2.0), Na_2_S_2_O_8_ (2.0)	DCE:H_2_O (1:1, 5.0)	90	19	51 (**3Aa**)
11	TBAB (2.5), Na_2_S_2_O_8_ (1.0)	DCE:H_2_O (1:1, 5.0)	90	19	69 (**3Aa**)
12	TBAB (2.0), K_2_S_2_O_8_ (1.0)	DCE:H_2_O (1:1, 5.0)	90	19	71 (**3Aa**)
13	TBAB (2.0), (NH_4_)_2_S_2_O_8_ (1.0)	DCE:H_2_O (1:1, 5.0)	90	19	69 (**3Aa**)
14	TBAB (2.0), Oxone^®^ (1.0)	DCE:H_2_O (1:1, 5.0)	90	19	37 (**3Aa**)

**Scheme 1:**
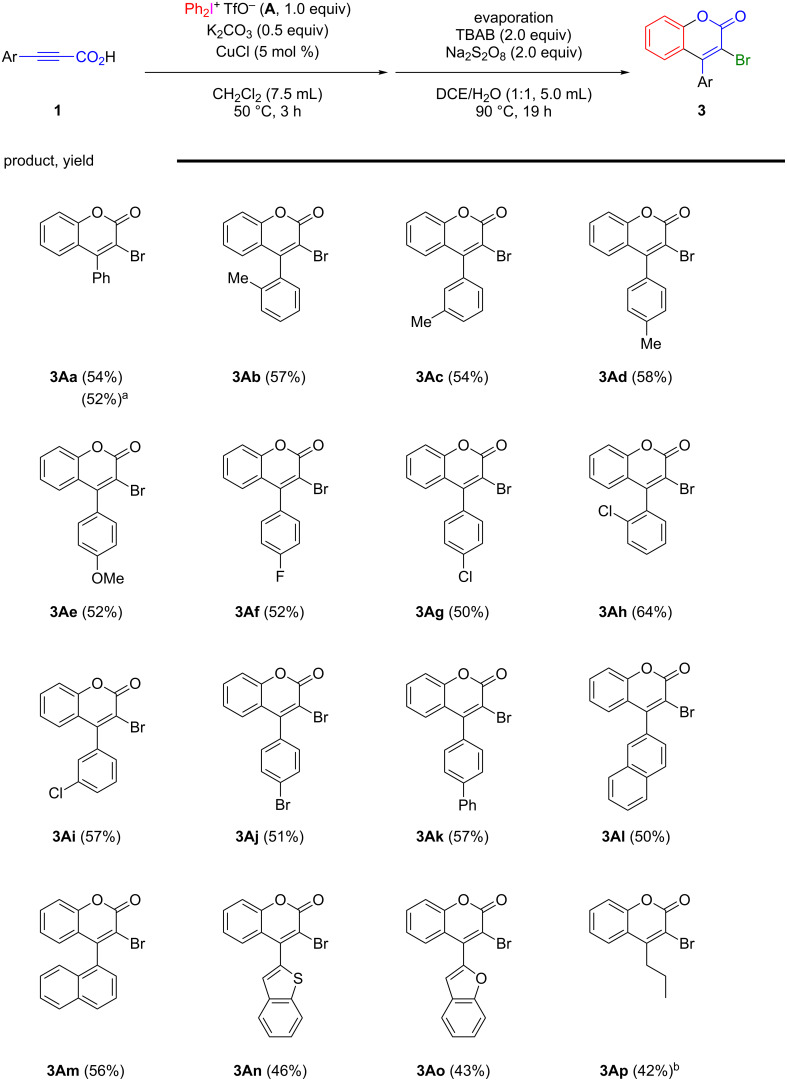
One-pot preparation of 4-aryl-3-bromocoumarins **3** from 3-aryl-2-propynoic acids **1** with diphenyliodonium triflate (**A**). ^a^3-Phenyl-2-propynoic acid (**1a**, 8.0 mmol) was used. ^b^The first reaction step was conducted with K_2_CO_3_ (1.0 equiv) under refluxing conditions.

Finally, based on the results in Table 1 and Table 2, the one-pot preparation of 4-aryl-3-bromocoumarins **3** from 3-aryl-2-propynoic acids **1** was carried out. 3-Aryl-2-propynoic acids **1**, such as 3-phenyl-2-propynoic acid (**1a**), 3-(*o*-methylphenyl)-2-propynoic acid (**1b**), 3-(*m*-methylphenyl)-2-propynoic acid (**1c**), 3-(*p*-methylphenyl)-2-propynoic acid (**1d**), 3-(*p*-methoxyphenyl)-2-propynoic acid (**1e**), 3-(*p*-fluorophenyl)-2-propynoic acid (**1f**), 3-(*p*-chlorophenyl)-2-propynoic acid (**1g**), 3-(*o*-chlorophenyl)-2-propynoic acid (**1h**), 3-(*m*-chlorophenyl)-2-propynoic acid (**1i**), 3-(*p*-bromophenyl)-2-propynoic acid (**1j**), 3-(*p*-biphenyl)-2-propynoic acid (**1k**), 3-(naphthalen-2’-yl)-2-propynoic acid (**1l**), and 3-(naphthalen-1’-yl)-2-propynoic acid (**1m**), were treated with diphenyliodonium triflate (**A**, 1.0 equiv) in the presence of CuCl and K_2_CO_3_ in CH_2_Cl_2_ for 3 h under refluxing conditions. After removal of the solvent, the second-step treatment of the reaction mixture with TBAB (2.0 equiv) and Na_2_S_2_O_8_ (2.0 equiv) in a mixture of DCE and water at 90 °C for 19 h gave 4-aryl-3-bromocoumarins **3Aa**–**3Am** in moderate yields, respectively, as shown in Scheme 1. As a gram-scale experiment, treatment of 3-phenyl-2-propynoic acid (**1a**, 8 mmol) with diphenyliodonium triflate **A** in the presence of CuCl and K_2_CO_3_ in CH_2_Cl_2_ for 3 h, followed by removal of the solvent and the reaction with TBAB and Na_2_S_2_O_8_ in a mixture of DCE and water at 90 °C for 19 h gave 3-bromo-4-phenylcoumarin (**3Aa**) in 52% yield. For 3-aryl-2-propynoic acids bearing heteroaromatic groups, treatment of 3-(benzothiophen-2’-yl)-2-propynoic acid (**1n**) and 3-(benzofuran-2’-yl)-2-propynoic acid (**1o**) under the same procedure and conditions gave the corresponding coumarins **3An** and **3Ao** in moderate yields, respectively. Under the present procedure and conditions, use of 2-hexynoic acid (**1p**), a 3-alkyl-2-propynoic acid, provided 3-bromo-4-propylcoumarin (**3Ap**) in 42% yield, as shown in Scheme 1.

**Scheme 2:**
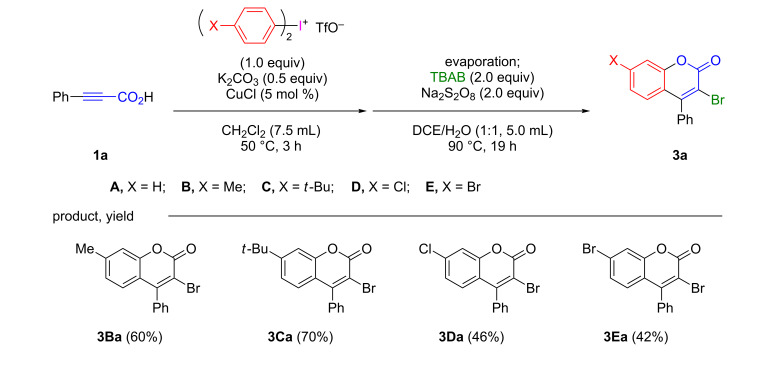
One-pot preparation of 3-bromo-4-phenylcoumarins **3a** from 3-phenyl-2-propynoic acid (**1a**) with daryliodonium triflates **B**–**E**.

Then, other diaryliodonium triflates were used instead of diphenyliodonium triflate (**A**). Treatment of 3-phenyl-2-propynoic acid (**1a**) with diaryliodonium triflates (1.0 equiv), such as di(*p*-methylphenyl)iodonium triflate (**B**), di(*tert*-butylphenyl)iodonium triflate (**C**), di(*p*-chlorophenyl)iodonium triflate (**D**), and di(*p*-bromophenyl)iodonium triflate (**E**), in the presence of CuCl and K_2_CO_3_ in CH_2_Cl_2_ for 3 h under refluxing conditions, followed by removal of the solvent and the reaction with TBAB (2.0 equiv) and Na_2_S_2_O_8_ (2.0 equiv) in a mixture of DCE and water at 90 °C for 19 h gave 3-bromo-4-phenylcoumarin derivatives **3Ba**–**3Ea** bearing methyl, *tert*-butyl, chloro, and bromo groups at 7-position in good to moderate yields, respectively, as shown in Scheme 2.

**Scheme 3:**
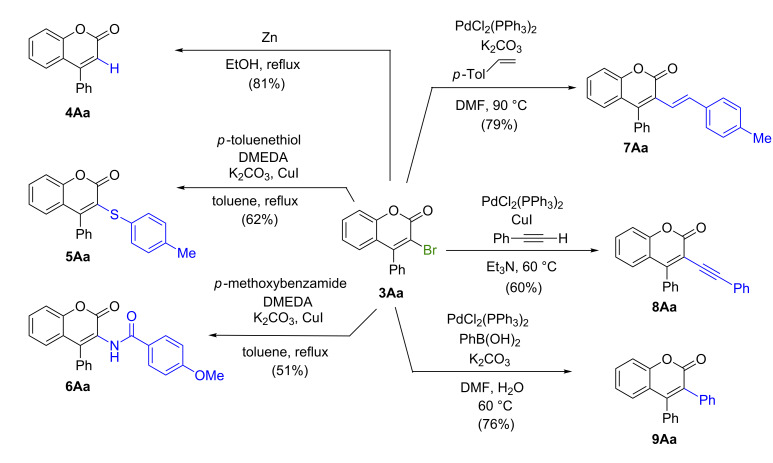
Derivatization of 3-bromo-4-phenylcoumarin.

As regards the synthetic utilization of the products in the present one-pot reaction, treatment of 3-bromo-4-phenylcoumarin (**3Aa**) with Zn in ethanol under refluxing conditions gave 4-phenylcoumarin (**4Aa**) in 81% yield. Treatment of 3-bromo-4-phenylcoumarin (**3Aa**) with *p*-toluenethiol/*N,N’*-dimethylethylenediamine (DMEDA)/K_2_CO_3_ in the presence of CuI in toluene at refluxing temperature and with *p*-methoxybenzamide/DMEDA/K_2_CO_3_ in the presence of CuI in toluene at refluxing temperature generated 3-(4-methylbenzenesulfenyl)-4-phenylcoumarin (**5Aa**) and 3-(4-methoxylbenzoylamino)-4-phenylcoumarin (**6Aa**) in 62 and 51% yields, respectively. The Pd-catalyzed coupling reactions of 3-bromo-4-phenylcoumarin (**3Aa**) with 4-methylstyrene/K_2_CO_3_/PdCl_2_(Ph_3_P)_2_, with phenylacetylene/PdCl_2_(Ph_3_P)_2_/Et_3_N and with PhB(OH)_2_/K_2_CO_3_/PdCl_2_(Ph_3_P)_2_ provided the corresponding C–C bonded coumarin derivatives **7Aa**, **8Aa**, and **9Aa** in 79, 60 and 76% yields, respectively (Scheme 3).

**Figure 1:**
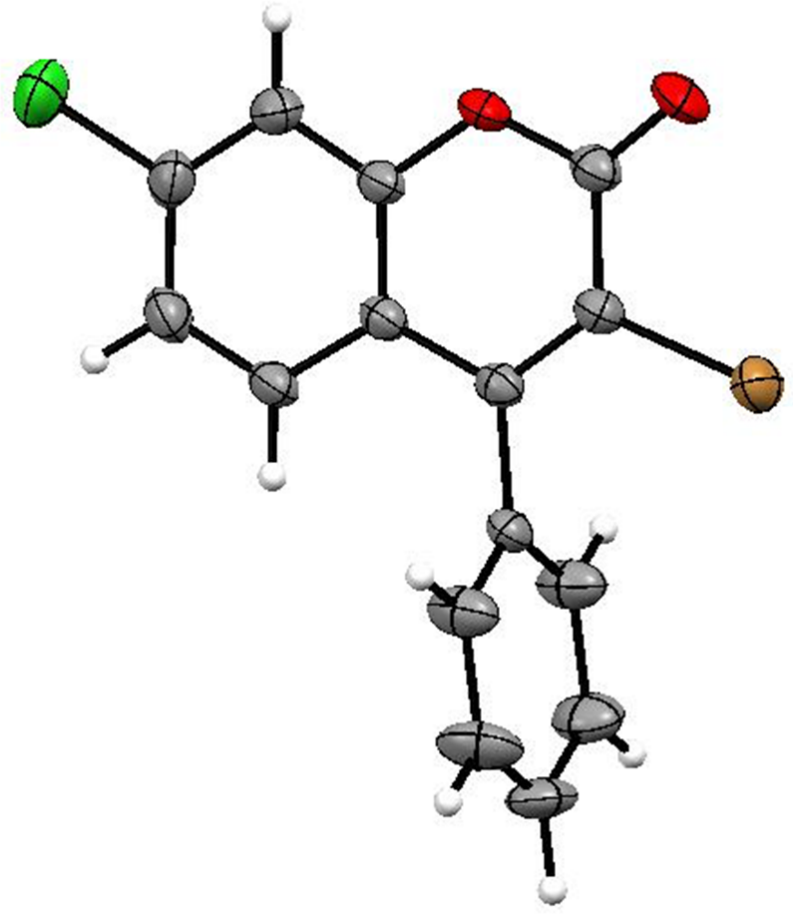
ORTEP of 3-bromo-7-chloro-4-phenylcoumarin (**3Da**).

**Scheme 4:**
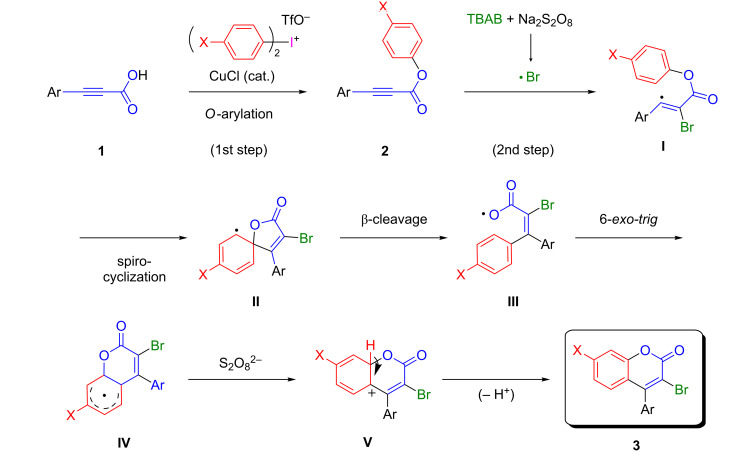
Possible reaction pathway.

To support the present bromocyclization reaction to form 4-aryl-3-bromocoumarins with TBAB and Na_2_S_2_O_8_ at the second step, the present one-pot preparation of 3-bromo-4-phenylcoumarin (**3Aa**) from 3-phenyl-2-propynoic acid (**1a**) was carried out in the presence of 2,2,6,6-tetramethylpiperidine 1-oxyl radical (TEMPO, 2.0 equiv) or 2,6-di(*tert*-butyl-*p*-cresol (BHT, 3.0 equiv) at the second step under the same procedure and conditions, but 3-bromo-4-phenylcoumarin (**3Aa**) was not obtained at all in both reactions. Thus, the present bromocyclization of the formed phenyl 3-phenyl-2-propynoate (**2Aa**) with TBAB and Na_2_S_2_O_8_ in a mixture of DCE and water is a radical-mediated bromocyclization reaction. X-ray crystallographic analysis of 3-bromo-7-chloro-4-phenylcoumarin (**3Da**), which was formed by the subsequent treatment of 3-phenyl-2-propynoic acid (**1a**) with di(*p*-chlorophenyl)iodonium triflate (**D**) and then with TBAB and Na_2_S_2_O_8_, was carried out, as shown in Figure 1. Based on those results, the possible reaction pathway is shown in Scheme 4.

The O-arylation of 3-aryl-2-propynoic acid **1** with diaryliodonium triflate in the presence of K_2_CO_3_ and CuCl occurs to form aryl 3-aryl-2-propynoate **2** (1st step). The bromocyclization of aryl 3-aryl-2-propynoate **2** with TBAB and Na_2_S_2_O_8_ proceeds via a bromoradical addition to the triple bond to form very reactive vinyl radical **I** [47]. *Ipso*-cyclization of the vinyl radical **I** occurs to form spiro radical intermediate **II**. Then, β-cleavage of the spiro radical intermediate **II** proceeds to form carboxyl radical **III**. *6-Exo-trig* cyclization of the carboxyl radical **III** onto the aromatic ring takes place to form adduct radical **IV**, which would be rapidly oxidized by Na_2_S_2_O_8_ to form cation intermediate **V**. Smooth deprotonation of cation intermediate **V** occurs to generate 4-aryl-3-bromocoumarin **3** (2nd step). The radical *ipso*-cyclization of the formed vinyl radical and its 1,2-carboxyl group migration agree with previously reported results [31–34].

## Conclusion

The successive treatment of 3-aryl-2-propynoic acids with diaryliodonium triflates in the presence of K_2_CO_3_ and CuCl, and then with tetrabutylammonium bromide (TBAB) and Na_2_S_2_O_8_ gave 4-aryl-3-bromocoumarins bearing hydrogen, methyl, *tert*-butyl, chloro, and bromo groups at 7-position in moderate yields, respectively. In one of the obtained 4-aryl-3-bromocoumarins, the C–Br bond of 3-bromo-4-phenylcoumarin was smoothly converted into 4-phenylcoumarins bearing C–H, C–S, C–N, and C–C bonds at 3-position. We believe the present method will be useful for the preparation of various 4-arylcoumarin derivatives due to its simple one-pot synthesis.

## Supporting Information

File 1 NMR charts of all coumarin derivatives **3Aa**–**3Ap**, **3Ba**–**3Ea**, and **4Aa**–**9Aa**, and X-ray analytical data of **3Da**.
