# Supplementary material for: One-pot preparation of 4-aryl-3-bromocoumarins from 4-aryl-2-propynoic acids with diaryliodonium salts, TBAB, and Na2S2O8
Source: Beilstein J Org Chem. 2018 Feb 5;14:345–53. doi: 10.3762/bjoc.14.22 (PMC5815302; doi:10.3762/bjoc.14.22)
Supplement: File 1 — NMR charts of all coumarin derivatives 3Aa–3Ap, 3Ba–3Ea, and 4Aa–9Aa, and X-ray analytical data of 3Da. [file Beilstein_J_Org_Chem-14-345-s001.pdf]

Supporting Information

for

One-pot preparation of 4-aryl-3-bromocoumarins from 4-aryl-2-propynoic acids with diaryliodonium salts, TBAB, and $Na_2S_2O_8$

Teppei Sasaki[1], Katsuhiko Moriyama[1,2] and Hideo Togo*[1]

Address: [1]Department of Chemistry, Graduate School of Science, Chiba University, Yayoi-cho 1-33, Inage-ku, Chiba 263-8522, Japan and [2]Molecular Chirality Research Center, Chiba University, Yayoi-cho 1-33, Inage-ku, Chiba 263-8522, Japan

Email: Hideo Togo - togo@faculty.chiba -u.jp

*Corresponding author

NMR charts of all coumarin derivatives 3Aa–3Ap, 3Ba–3Ea, and 4Aa–9Aa, and X-ray analytical data of 3Da

Table of contents

Experimental section for compounds **3** and **4Aa–9Aa** ------------------ S1–S10

^{1}H NMR and ^{13}C NMR spectra of compounds **3** and **4Aa–9Aa** ------- S11–S36

General

^{1}H NMR spectra were measured on 400 MHz spectrometers. Chemical shifts were recorded as follows: chemical shift in ppm from internal tetramethylsilane on the δ scale, multiplicity (s = singlet; d = doublet; t = triplet; q = quartet; m = multiplet; br = broad), coupling constant (Hz), integration, and assignment. ^{13}C NMR spectra were measured on 100 MHz spectrometers. Chemical shifts were recorded in ppm from the solvent resonance employed as the internal standard (deuterochloroform at 77.0 ppm). Characteristic peaks in the infrared (IR) spectra were recorded in wave number, cm^{-1}. High-resolution mass spectra were recorded by Thermo Fisher Scientific Exactive Orbitrap mass spectrometers. Melting points were uncorrected. Thin-layer chromatography (TLC) was performed using 0.25 mm silica gel plates (60F-254). The products were purified by column chromatography on neutral silica gel 60N (63–210 mesh).

Typical procedure for the one-pot conversion of 3-aryl-2-propynoic acids 1 into 4-aryl-3-bromocoumarins 3

To a mixture of 3-phenyl-2-propynoic acid (**1a**, 0.5 mmol, 73.1 mg), CuCl (5 mol %, 2.5 mg), and K_2CO_3 (0.25 mmol, 34.6 mg) in CH_2Cl_2 (7.5 mL) was added diphenyliodonium trifluoromethanesulfonate (0.5 mmol, 215.1 mg). The flask was flushed with argon gas, and then the obtained mixture was stirred for 3 h at 50 °C. The solvent was removed, and TBAB (1.0 mmol, 322.4 mg), $Na_2S_2O_8$ (1.0 mmol, 238.1 mg), and DCE/H_2O (1:1, 5.0 mL) were added to the residue. The obtained mixture was stirred for 19 h at 90 °C. Water (5.0 mL) was added to the reaction mixture and the product was extracted with $CHCl_3$ (15 mL × 3). The organic layer was dried over Na_2SO_4. After removal of the solvent under reduced pressure, the residue was purified by silica-gel column chromatography (eluent: *n*-hexane/EtOAc = 9:1) to give 3-bromo-4-phenylcoumarin (**3Aa**, 81.5 mg, 54% yield).

3-Bromo-4-phenylcoumarin (3Aa)[1]: Yield: 81.5 mg (54%); white solid; Mp: 146-147 °C; IR (neat) 1725, 1273, 1034 cm^{-1}; ^{1}H-NMR (400 MHz, $CDCl_3$): δ = 7.08 (dd, 1H, *J* = 8.0, 1.6 Hz), 7.20 (td, 1H, *J* = 7.8, 1.1 Hz), 7.29-7.32 (m, 2H), 7.42 (dd, 1H, *J* = 8.3, 0.9 Hz), 7.52-7.60 (m, 4H); ^{13}C-NMR (100 MHz, $CDCl_3$): δ = 112.5, 116.7, 120.2, 124.7, 127.6, 128.0, 128.8, 129.3, 132.0, 135.2, 152.4, 154.6, 157.3; HRMS (APCI) Calcd for $C_{15}H_{10}O_2Br$ $[M+H]^+$ = 300.9859, Found = 300.9857.

3-Bromo-4-(2'-methylphenyl)coumarin (3Ab): Yield: 90.3 mg (57%); white solid; Mp: 99-100 °C; IR (neat) 1729, 1275, 1035 cm^{-1}; ^{1}H-NMR (400 MHz, $CDCl_3$): δ = 2.12 (s, 3H), 6.93 (dd, 1H, *J* = 7.9, 1.4 Hz), 7.10 (d, 1H, *J* = 7.9 Hz), 7.18 (td, 1H, *J* = 7.4, 1.1 Hz), 7.35-7.46 (m, 4H), 7.57 (td, 1H, *J* = 7.6, 1.6 Hz); ^{13}C-NMR (100 MHz, $CDCl_3$): δ = 19.2, 112.9, 116.8, 119.9, 124.9, 126.3, 127.1, 127.4, 129.4, 130.6, 132.1, 134.8, 134.9, 152.4, 155.0, 157.3; HRMS (APCI) Calcd for $C_{16}H_{12}O_2Br$ $[M+H]^+$ = 315.0015, Found = 315.0015.

3-Bromo-4-(3'-methylphenyl)coumarin (3Ac): Yield: 85.3 mg (54%); colorless oil; IR (neat) 1725, 1276, 1037 cm^{-1}; ^{1}H-NMR (400 MHz, $CDCl_3$): δ = 2.45 (s, 3H), 7.10 (d, 1H, *J* = 8.7 Hz), 7.10 (d, 1H, *J* = 7.9 Hz), 7.10 (s, 1H), 7.19 (td, 1H, *J* = 7.9, 1.1 Hz), 7.34 (d, 1H, *J* = 7.5 Hz), 7.41 (dd, 1H, *J* = 8.4, 0.9 Hz), 7.45 (t, 1H, *J* = 7.9 Hz), 7.56 (td, 1H, *J* = 7.8, 1.6 Hz); ^{13}C-NMR (100 MHz, $CDCl_3$): δ = 21.5, 112.4, 116.7, 120.3, 124.6, 125.0, 127.7, 128.4, 128.7, 130.0, 132.0, 135.1, 138.6, 152.4, 154.8, 157.4; HRMS (APCI) Calcd for $C_{16}H_{12}O_2Br$ $[M+H]^+$ = 315.0015, Found = 315.0015.

3-Bromo-4-(4'-methylphenyl)coumarin (3Ad)[2]: Yield: 91.4 mg (58%); white solid; Mp: 163-164 °C; IR (neat) 1721, 1273, 1038 cm^{-1}; ^{1}H-NMR (400 MHz, $CDCl_3$): δ = 2.48 (s, 3H), 7.12 (dd, 1H, *J* = 7.9, 1.6 Hz), 7.16-7.51 (m, 3H), 7.37 (d, 2H, *J* = 7.9 Hz), 7.41 (dd, 1H, *J* = 8.4, 0.9 Hz), 7.56 (td, 1H, *J* = 7.7, 1.8 Hz); ^{13}C-NMR (100 MHz, $CDCl_3$): δ = 21.4, 112.6, 116.8, 120.4, 124.6, 127.7, 128.0, 129.5, 132.0, 132.3, 139.4, 152.4, 154.8, 157.5; HRMS (APCI) Calcd for $C_{16}H_{12}O_2Br$ $[M+H]^+$ = 315.0015, Found = 315.0015.

3-Bromo-4-(4'-methoxyphenyl)coumarin (3Ae)[2]: Yield: 86.2 mg (52%); pale yellow solid; Mp: 167-168 °C; IR (neat) 1730, 1251, 1034 cm^{-1}; ^{1}H-NMR (400 MHz, $CDCl_3$): δ = 3.91 (s, 3H), 7.08 (d, 2H, *J* = 8.8 Hz), 7.16 (dd, 1H, *J* = 8.2, 1.8 Hz), 7.20 (td, 1H, *J* = 7.6, 1.1 Hz), 7.26 (d, 2H, *J* = 8.8 Hz), 7.41 (d, 1H, *J* = 8.4 Hz), 7.56 (td, 1H, *J* = 7.6, 2.0 Hz); ^{13}C-NMR (100 MHz, $CDCl_3$): δ = 55.3, 112.7, 114.1, 116.7, 120.4, 124.6, 127.2, 127.7, 129.7, 131.9, 152.3, 134.4, 157.4, 160.2; HRMS (APCI) Calcd for $C_{16}H_{12}O_3Br$ $[M+H]^+$ = 330.9964, Found = 330.9962.

3-Bromo-4-(4'-fluorophenyl)coumarin (3Af)[2]: Yield: 82.3 mg (52%); white solid; Mp: 124-125 °C; IR (neat) 1710, 1275, 1038 cm^{-1}; ^{1}H-NMR (400 MHz, $CDCl_3$): δ = 7.07 (dd, 1H, *J* = 8.0, 1.4 Hz), 7.21 (td, 1H, *J* = 7.6, 1.1 Hz), 7.23-7.33 (m, 4H), 7.42 (dd, 1H, *J* = 8.4, 0.9 Hz), 7.58 (td, 1H, *J* = 7.8, 1.6 Hz); ^{13}C-NMR (100 MHz, $CDCl_3$): δ

= 113.1, 116.2 (J_{C-F} = 21.9 Hz), 116.9, 120.2, 124.8, 127.4, 130.2 (J_{C-F} = 8.6 Hz), 131.1 (J_{C-F} = 3.8 Hz), 132.2, 152.4, 153.7, 157.2, 163.1 (J_{C-F} = 249.9 Hz); HRMS (APCI) Calcd for $C_{15}H_9O_2BrF$ $[M+H]^+$ = 318.9764, Found = 318.9764

3-Bromo-4-(4'-chlorophenyl)coumarin (3Ag)[2]: Yield: 152.2 mg (50%); white solid; Mp: 152-153 °C; IR (neat) 1717, 1277, 1037 cm^{-1}; ^1H-NMR (400 MHz, $CDCl_3$): δ = 7.06 (dd, 1H, J = 8.2, 1.6 Hz), 7.21 (td, 1H, J = 7.6, 1.1 Hz), 7.25-7.28 (m, 2H), 7.42 (dd, 1H, J = 8.4, 1.1 Hz), 7.56 (d, 2H, J = 8.4 Hz), 7.58 (td, 1H, J = 7.8, 1.6 Hz); ^{13}C-NMR (100 MHz, $CDCl_3$): δ =112.9, 117.0, 120.0, 124.8, 127.3, 129.3, 129.6, 132.3, 133.5, 135.6, 152.4, 153.5, 157.1; HRMS (APCI) Calcd for $C_{15}H_9O_2BrCl$ $[M+H]^+$ = 334.9469, Found = 334.9468.

3-Bromo-4-(2'-chlorophenyl)coumarin (3Ah): Yield: 106.7 mg (64%); white solid; Mp: 162-163 °C; IR (neat) 1732, 1277, 1036 cm^{-1}; ^1H-NMR (400 MHz, $CDCl_3$): δ = 6.94 (dd, 1H, J = 7.9, 1.4 Hz), 7.21 (t, 1H, J = 7.7 Hz), 7.25 (dd, 1H, J = 7.3, 2.0 Hz) , 7.43 (d, 1H, J = 8.4 Hz), 7.45-7.53 (m, 2H), 7.56-7.62 (m, 2H); ^{13}C-NMR (100 MHz, $CDCl_3$): δ = 113.6, 116.8, 119.4, 124.9, 126.8, 127.4, 129.4, 130.0, 130.8, 132.0, 132.1, 134.2, 152.3, 152.4, 157.1; HRMS (APCI) Calcd for $C_{15}H_9O_2BrCl$ $[M+H]^+$ = 334.9469, Found = 334.9468.

3-Bromo-4-(3'-chlorophenyl)coumarin (3Ai): Yield: 95.2 mg (57%); white solid; Mp: 138-139 °C; IR (neat) 1715, 1276, 1038 cm^{-1}; ^1H-NMR (400 MHz, $CDCl_3$): δ = 7.06 (dd, 1H, J = 8.0, 1.6 Hz), 7.18-7.25 (m, 2H), 7.32 (s, 1H), 7.42 (d, 1H, J = 8.4 Hz), 7.49-7.55 (m, 2H), 7.59 (td, 1H, J = 8.0, 1.6 Hz); ^{13}C-NMR (100 MHz, $CDCl_3$): δ = 112.8, 116.8, 119.7, 124.8, 126.3, 127.2, 128.0, 129.5, 130.3, 132.2, 134.8, 136.7, 152.2, 152.9, 156.9; HRMS (APCI) Calcd for $C_{15}H_9O_2BrCl$ $[M+H]^+$ = 334.9469, Found = 334.9468.

3-Bromo-4-(4'-bromophenyl)coumarin (3Aj): Yield: 95.2 mg (51%); white solid; Mp: 154-155 °C; IR (neat) 1718, 1276, 1037 cm^{-1}; ^1H-NMR (400 MHz, $CDCl_3$): δ = 7.06 (dd, 1H, J = 7.9, 1.6 Hz), 7.18-7.23 (m, 3H), 7.42 (dd, 1H, J = 8.4, 1.1 Hz), 7.58 (td, 1H, J = 7.9, 1.6 Hz), 7.72 (d, 2H, J = 8.6 Hz); ^{13}C-NMR (100 MHz, $CDCl_3$): δ = 112.8, 116.9, 119.9, 123.8, 124.8, 127.2, 129.8, 132.2, 132.2, 134.0, 152.4, 153.4, 157.1; HRMS (APCI) Calcd for $C_{15}H_9O_2Br_2$ $[M+H]^+$ = 378.8964, Found = 378.8966.

4-(Biphenyl-4'-yl)-3-bromocoumarin (3Ak): Yield: 107.6 mg (57%); yellow solid;

Mp: 199-200 °C; IR (neat) 1733, 1275, 1036 cm^{-1}; ^1H-NMR (400 MHz, $CDCl_3$): δ = 7.18 (dd, 1H, *J* = 8.0, 1.8 Hz), 7.22 (td, 1H, *J* = 7.4, 1.1 Hz), 7.39 (d, 2H, *J* = 8.4 Hz), 7.43 (d, 2H, *J* = 8.6 Hz), 7.51 (t, 2H, *J* = 7.3 Hz), 7.58 (td, 1H, *J* = 7.6, 2.0 Hz), 7.69 (d, 2H, *J* = 7.5 Hz), 7.79 (d, 2H, *J* = 8.4 Hz); ^{13}C-NMR (100 MHz, $CDCl_3$): δ = 112.6, 116.8, 120.2, 124.7, 127.1, 127.4, 127.6, 127.9, 128.6, 128.9, 132.0, 134.0, 139.9, 142.1, 152.4, 154.4, 157.3; HRMS (APCI) Calcd for $C_{21}H_{14}O_2Br$ $[M+H]^+$ = 377.0172, Found = 377.0170.

3-Bromo-4-(naphthalen-2'-yl)coumarin (3Al): Yield: 88.3 mg (50%); white solid; Mp: 180-181 °C; IR (neat) 1721, 1275, 1038 cm^{-1}; ^1H-NMR (400 MHz, $CDCl_3$): δ = 7.11 (dd, 1H, *J* = 8.0, 1.6 Hz), 7.17 (td, 1H, *J* = 7.6, 1.1 Hz), 7.40 (dd, 1H, *J* = 8.4, 1.8 Hz), 7.44 (dd, 1H, *J* = 8.3, 0.7 Hz), 7.55-7.65 (m, 3H), 7.81 (s, 1H), 7.90-7.99 (m, 2H), 8.04 (d, 1H, *J* = 8.6 Hz); ^{13}C-NMR (100 MHz, $CDCl_3$): δ = 112.8, 116.8, 120.3, 124.7, 125.3, 126.9, 127.2, 127.7, 127.7, 127.9, 128.3, 128.7, 132.1, 132.5, 132.8, 133.2, 152.4, 154.6, 157.4; HRMS (APCI) Calcd for $C_{19}H_{12}O_2Br$ $[M+H]^+$ = 351.0015, Found = 351.0013.

3-Bromo-4-(naphthalen-1'-yl)coumarin (3Am): Yield: 98.3 mg (56%); white solid; Mp: 106-107 °C; IR (neat) 1731, 1277, 1035 cm^{-1}; ^1H-NMR (400 MHz, $CDCl_3$): δ = 6.83 (dd, 1H, *J* = 8.0, 1.6 Hz), 7.08 (dt, 1H, *J* = 7.6, 1.1 Hz), 7.39 (dd, 1H, *J* = 7.0, 1.1 Hz), 7.44-7.48 (m, 3H), 7.54-7.58 (m, 2H), 7.65 (t, 1H, *J* = 7.6 Hz), 7.99 (d, 1H, *J* = 8.2 Hz), 8.04 (d, 1H, *J* = 8.2 Hz); ^{13}C-NMR (100 MHz, $CDCl_3$): δ = 114.1, 116.8, 120.6, 124.6, 124.8, 125.4, 126.0, 126.7, 127.2, 127.7, 128.7, 129.6, 129.7, 132.1, 132.8, 133.5, 152.3, 154.1, 157.3; HRMS (APCI) Calcd for $C_{19}H_{12}O_2Br$ $[M+H]^+$ = 351.0015, Found = 351.0014.

4-(Benzo[*b*]thiophen-2'-yl)-3-bromocoumarin (3An): Yield: 82.7 mg (46%); red solid; Mp: 214-215 °C; IR (neat) 1722, 1272, 1038 cm^{-1}; ^1H-NMR (400 MHz, $CDCl_3$): δ = 7.23 (td, 1H, *J* = 7.7, 0.9 Hz), 7.39 (dd, 1H, *J* = 8.0, 1.4 Hz), 7.39 (s, 1H), 7.43 (d, 1H, *J* = 8.4 Hz), 7.45-7.51 (m, 2H), 7.59 (td, 1H, *J* = 7.8, 1.6 Hz), 7.88-7.96 (m, 2H);^{13}C-NMR (100 MHz, $CDCl_3$): δ = 115.2, 116.8, 120.2, 122.4, 124.4, 124.9, 125.0, 125.5, 125.8, 127.4, 132.3, 134.8, 139.1, 140.6, 148.5, 152.1, 156.9; HRMS (APCI) Calcd for $C_{17}H_{10}O_2BrS$ $[M+H]^+$ = 356.9579, Found = 356.9579.

4-(Benzofuran-2'-yl)-3-bromocoumarin (3Ao): Yield: 73.0 mg (43%); white solid; Mp: 129-130 °C; IR (neat) 1739, 1278, 1038 cm^{-1}; ^1H-NMR (400 MHz, $CDCl_3$): δ =

7.28-7.31 (m, 2H), 7.37 (t, 1H, J = 7.7 Hz), 7.41-7.48 (m, 2H), 7.57-7.64 (m, 3H), 7.75 (d, 1H, J = 7.9 Hz); ^{13}C-NMR (100 MHz, $CDCl_3$): δ = 111.7, 111.8, 114.3, 117.0, 118.8, 122.0, 123.8, 124.9, 126.1, 127.1, 127.4, 132.3, 143.5, 148.1, 152.3, 155.0, 157.1; HRMS (APCI) Calcd for $C_{17}H_{10}O_3Br$ $[M+H]^+$ = 340.9808, Found = 340.9808.

3-Bromo-4-propylcoumarin (3Ap): Yield: 55.6 mg (42%); white solid; Mp: 58-59 °C; IR (neat) 2931, 1720, 1272, 1036 cm^{-1}; ^{1}H-NMR (400 MHz, $CDCl_3$): δ = 1.13 (s, 3H, J = 7.3 Hz), 1.72 (sextet, 2H, J = 8.2 Hz), 3.03 (t, 2H, J = 8.2 Hz), 7.34 (td, 1H, J = 7.7, 1.1 Hz), 7.37 (d, 1H, J = 8.2 Hz), 7.57 (td, 1H, J = 7.7, 1.4 Hz), 7.66 (dd, 1H, J = 8.2, 1.4 Hz); ^{13}C-NMR (100 MHz, $CDCl_3$): δ = 14.3, 21.5, 34.6, 112.7, 117.2, 118.9, 124.8, 124.8, 131.8, 152.1, 154.7, 157.1; HRMS (APCI) Calcd for $C_{12}H_{12}O_2Br$ $[M+H]^+$ = 267.0015, Found = 267.0015.

3-Bromo-7-methyl-4-phenycoumarin (3Ba): Yield: 95.2 mg (60%); white solid; Mp: 186-187 °C; IR (neat) 1722, 1255, 1022 cm^{-1}; ^{1}H-NMR (400 MHz, $CDCl_3$): δ = 2.44 (s, 3H), 6.95 (d, 1H, J = 8.2 Hz), 7.00 (dd, 1H, J = 8.2, 0.9 Hz), 7.21 (s, 1H), 7.29 (dd, 2H, J = 7.6, 1.4 Hz), 7.51-7.59 (m, 3H); ^{13}C-NMR (100 MHz, $CDCl_3$): δ = 21.6, 111.2, 116.8, 117.9, 125.9, 127.2, 128.0, 128.7, 129.2, 135.3, 143.5, 152.4, 154.6, 157.6; HRMS (APCI) Calcd for $C_{16}H_{12}O_2Br$ $[M+H]^+$ = 315.0015, Found = 315.0014.

3-Bromo-7-(*tert*-butyl)-4-phenylcoumarin (3Ca): Yield: 124.6 mg (70%); white solid; Mp: 132-133 °C; IR (neat) 2951, 1718, 1281, 1031 cm^{-1}; ^{1}H-NMR (400 MHz, $CDCl_3$): δ = 1.34 (s, 9H), 7.01 (d, 1H, J = 8.4 Hz), 7.22 (dd, 1H, J = 8.4, 1.8 Hz), 7.27-7.33 (m, 2H), 7.41 (d, 1H, J = 1.8 Hz), 7.50-7.58 (m, 3H); ^{13}C-NMR (100 MHz, $CDCl_3$): δ = 30.8, 35.1, 111.3, 113.4, 117.7, 122.2, 127.1, 127.9, 128.7, 129.2, 135.2, 152.3, 154.4, 156.6, 157.6; HRMS (APCI) Calcd for $C_{19}H_{18}O_2Br$ $[M+H]^+$ = 357.0485, Found = 357.0484.

3-Bromo-7-chloro-4-phenylcoumarin (3Da): Yield: 77.6 mg (46%); white solid; Mp: 191-192 °C; IR (neat) 1731, 1237, 1034 cm^{-1}; ^{1}H-NMR (400 MHz, $CDCl_3$): δ = 7.01 (d, 1H, J = 8.6 Hz), 7.16 (dd, 1H, J = 8.7, 1.8 Hz), 7.27-7.30 (m, 2H), 7.42 (s, 1H), 7.52-7.60 (m, 3H); ^{13}C-NMR (100 MHz, $CDCl_3$): δ = 112.5, 117.0, 118.9, 125.3, 127.9, 128.4, 128.9, 129.5, 134.8, 137.9, 152.5, 153.9, 156.7; HRMS (APCI) Calcd for $C_{15}H_9O_2BrCl$ $[M+H]^+$ = 334.9469, Found = 334.9469.

Crystal Data for 3-Bromo-7-chloro-4-phenylcoumarin 3Da: Formula $C_{15}H_8BrClO_2$, colorless, crystal dimensions 0.20 × 0.20 × 0.10 mm^3, Plate, space group P 1 21/c 1', a = 9.6791(10) Å, b = 15.5782(17) Å, c = 8.9056(10) Å, α = 90°, β = 102.9168(15)°, γ = 90°, V = 1308.8(2) $Å^3$, Z = 4, ρ_{calc} = 1.269 g cm^{-3}, F(000) = 664, μ(MoKα) = 3.337 mm^{-1}, T = 173 K. 7298 reflections collected, 2994 independent reflections with $I > 2\sigma(I)$ ($2\theta_{max}$ = 27.56°), and 172 parameters were used for the solution of structure. The non-hydrogen atoms were refined anisotropically. R_1 = 0.0286 and wR_2 = 0.0657. GOF = 1.053. Crystallographic data (excluding structure factors) for the structure reported in this paper have been deposited with the Cambridge Crystallographic Data Centre as supplementary publication no. CCDC-1587945. Copies of the data can be obtained free of charge on application to CCDC, 12 Union Road, Cambridge CB2 1EZ, UK [Fax: int. code + 44(1223)336-033; E-mail: deposit@ccdc.cam.ac.uk].

3,7-Dibromo-4-phenylcoumarin (3Ea): Yield: 79.5 mg (42%); white solid; Mp: 186-187 °C; IR (neat) 1730, 1235, 1069 cm^{-1}; ^{1}H-NMR (400 MHz, $CDCl_3$): δ = 6.94 (d, 1H, J = 8.6 Hz), 7.27-7.33 (m, 3H), 7.54-7.61 (m, 4H); ^{13}C-NMR (100 MHz, $CDCl_3$): δ = 112.7, 119.3, 119.9, 125.9, 127.9, 128.1, 128.6, 129.0, 129.5, 134.7, 152.4, 154.0, 156.7; HRMS (APCI) Calcd for $C_{15}H_9O_2Br_2$ $[M+H]^+$ = 378.8964, Found = 378.8964.

Preparation of 4-phenylcoumarin (4Aa)

To a mixture of 3-bromo-4-phenylcoumarin (**3Aa**, 0.5 mmol, 150.6 mg) in EtOH (7.5 mL) was added Zn powder (5 mmol, 363.3 mg). Under argon atmosphere, the obtained mixture was stirred for 16 h at refluxing temperature. The cooled mixture was filtered through celite, and then the filtrate was concentrated under reduced pressure. The residue was purified by column chromatography on silica gel (eluent: *n*-hexane/AcOEt = 9:1) to afford 4-phenylcoumarin (**4Aa**, 89.9 mg, 81% yield).

4-Phenylcoumarin (4Aa)[3]: Yield: 89.9 mg (81%); white solid; IR (neat) 1722, 1277, 1034 cm^{-1}; ^{1}H-NMR (400 MHz, $CDCl_3$): δ = 6.39 (s, 1H), 7.24 (td, 1H, J = 7.7, 0.9 Hz), 7.42 (dd, 1H, J = 8.3, 0.9 Hz), 7.45-7.59 (m, 7H); ^{13}C-NMR (100 MHz, $CDCl_3$): δ = 115.2, 117.3, 119.0, 124.1, 127.0, 128.4, 128.8, 129.7, 131.9, 135.2, 154.2, 155.7, 160.8; HRMS (APCI) Calcd for $C_{15}H_{11}O_2$ $[M+H]^+$ = 223.0754, Found = 223.0753.

Preparation of 4-phenyl-3-(4'-methylbenzenesulfenyl)coumarin (5Aa)

To a mixture of 3-bromo-4-phenylcoumarin (**3Aa**, 0.5 mmol, 150.6 mg) and K_2CO_3 (1.5 mmol, 103.7 mg), CuI (0.010 mmol, 9.6 mg), and DMEDA (0.020 mmol, 10μL) in

toluene (2.0 mL) was added *p*-toluenethiol (1.2 mmol, 74.6 mg). Under argon atmosphere, the obtained mixture was stirred for 2 h at 135 °C. Saturated $NaHCO_3$ aqueous solution (5.0 mL) was added to the reaction mixture, and the product was extracted with $CHCl_3$ (15 mL × 3). The organic layer was dried over Na_2SO_4. After removal of the solvent under reduced pressure, the residue was purified by silica-gel column chromatography (eluent: *n*-hexane/EtOAc = 9:1) to give 4-phenyl-3-(4'-methylbenzenesulfenyl)coumarin (**5Aa**, 106.6 mg, 62% yield).

4-Phenyl-3-(4'-methylbenzenesulfenyl)coumarin (5Aa): Yield: 106.6 mg (62%); yellow oil; IR(neat) 1719, 1276, 1036 cm^{-1}; ^{1}H-NMR (400 MHz, $CDCl_3$): δ = 2.28 (s, 3H), 7.02 (d, 2H, *J* = 7.9 Hz), 7.08-7.20 (m, 3H), 7.18 (td, 1H, *J* = 7.8, 1.1 Hz) , 7.24-7.28 (m, 1H), 7.39 (dd, 1H, *J* = 8.4, 0.9 Hz), 7.47-7.60 (m, 5H); ^{13}C-NMR (100 MHz, $CDCl_3$): δ = 21.0, 116.7, 120.4, 122.3, 124.2, 128.0, 128.3, 128.4, 128.9, 129.7, 129.8, 131.0, 132.0, 134.8, 136.8, 153.2, 158.3, 159.1; HRMS (APCI) Calcd for $C_{22}H_{17}O_2S$ $[M+H]^+$ = 345.0944, Found = 345.0943.

Preparation of 3-(4'-methoxybenzoylamino)-4-phenylcoumarin (6Aa)

To a mixture of 3-bromo-4-phenylcoumarin (**3Aa**, 0.5 mmol, 150.6 mg) and K_2CO_3 (1.5 mmol, 103.7 mg), CuI (0.010 mmol, 9.6 mg), and DMEDA (0.020 mmol, 10μL) in toluene (2.0 mL) was added *p*-methoxybenzamide (1.2 mmol, 90.7 mg). Under argon atmosphere, the obtained mixture was stirred for 2 h at 135 °C. Saturated $NaHCO_3$ aqueous solution (5.0 mL) was added to the reaction mixture, and the product was extracted with $CHCl_3$ (15 mL × 3). The organic layer was dried over Na_2SO_4. The organic layer was dried over Na_2SO_4. After removal of the solvent under reduced pressure, the residue was purified by silica-gel column chromatography (eluent: *n*-hexane/EtOAc = 1:1) to give *N*-(4'-phenylcoumarin-3'-yl)-4-methoxybenzamide (**6Aa**, 95.2 mg, 51% yield).

3-(4'-Methoxybenzoylamino)-4-phenylcoumarin (6Aa): Yield: 95.2 mg (51%); white solid; Mp: 234-235 °C; IR(neat) 1718, 1654, 1605, 1254, 1029 cm^{-1}; ^{1}H-NMR (400 MHz, $CDCl_3$): δ = 3.83 (s, 3H), 6.87 (d, 2H, *J* = 8.8 Hz) , 7.23 (td, 1H, *J* = 7.6, 1.1 Hz), 7.33 (dd, 1H, *J* = 8.0, 1.4 Hz) , 7.37-7.49 (m, 6H), 7.54 (td, 1H, *J* = 7.6, 1.6 Hz), 7.64 (d, 2H, *J* = 8.8 Hz); ^{13}C-NMR (100 MHz, $CDCl_3$): δ = 55.4, 113.7, 116.8, 120.2, 120.8, 124.6, 125.7, 127.2, 128.4, 128.5, 128.9, 129.3, 131.2, 133.1, 147.2, 151.9, 160.0, 162.6, 165.3; HRMS (APCI) Calcd for $C_{23}H_{18}O_4N$ $[M+H]^+$ = 372.1230, Found = 372.1228.

Preparation of (*E*)-3-(4'-methylstyryl)-4-phenylcoumarin (7Aa)

To a mixture of 3-bromo-4-phenylcoumarin **(3Aa)** (0.5 mmol, 150.6 mg), K_2CO_3 (1.0 mmol, 138.2 mg), and $PdCl_2(PPh_3)_2$ (0.010 mmol, 7.0 mg) in DMF (5.0 mL) was added 4-methylstyrene (1.0 mmol, 68 μL). Under argon atmosphere, the obtained mixture was stirred for 3 h at 90 °C. Saturated $NaHCO_3$ aqueous solution (5.0 mL) was added to the reaction mixture, and the product was extracted with EtOAc (15 mL × 3), and washed with brine (15 mL). The organic layer was dried over Na_2SO_4. After removal of the solvent under reduced pressure, the residue was purified by silica-gel column chromatography (eluent: *n*-hexane:EtOAc = 9:1) to give (*E*)-3-(4'-methylstyryl)-4-phenylcoumarin (**7Aa**) (133.4 mg, 79% yield).

(*E*)-3-(4'-Methylstyryl)-4-phenylcoumarin (7Aa): Yield: 133.4 mg (79%); light yellow solid; Mp: 129-130 °C; IR (neat) 3067, 1715, 1256, 969 cm^{-1}; ^{1}H-NMR (400 MHz, $CDCl_3$): δ = 2.30 (s, 3H), 6.65 (d, 1H, *J* = 16.2 Hz), 7.04-7.08 (m, 3H), 7.12-7.17 (m, 3H), 7.29-7.33 (m, 2H), 7.38 (dd, 1H, *J* = 8.2, 1.1 Hz), 7.48 (td, 1H, *J* = 7.8, 1.6 Hz), 7.52-7.60 (m, 3H), 7.91 (d, 1H, *J* = 16.2 Hz); ^{13}C-NMR (100 MHz, $CDCl_3$): δ =21.3, 116.4, 120.8, 120.9, 121.6, 124.1, 126.8, 127.4, 128.8, 128.9, 129.0, 129.3, 130.9, 134.7, 134.9, 135.6, 138.1, 149.8, 152.1, 160.0; HRMS (APCI) Calcd for $C_{24}H_{19}O_2$ $[M+H]^+$ = 339.1380, Found = 339.1377.

Preparation of 4-phenyl-3-(phenylethynyl)coumarin (8Aa)

To a mixture of 3-bromo-4-phenylcoumarin (**3Aa**, 0.5 mmol, 150.6 mg) and CuI (0.010 mmol, 1.9 mg) and $PdCl_2(PPh_3)_2$ (0.010 mmol, 7.0 mg) in Et_3N (2.5 mL) was added ethynylbenzene (0.6 mmol, 66 μL). Under argon atmosphere, the obtained mixture was stirred for 3 h at 60 °C. Water (2.5 mL) was added to the reaction mixture, and the product was extracted with EtOAc (15 mL × 3), and washed with brine (15 mL). The organic layer was dried over Na_2SO_4. After removal of the solvent under reduced pressure, the residue was purified by silica-gel column chromatography (eluent: *n*-hexane:EtOAc = 9:1) to give 4-phenyl-3-(phenylethynyl)coumarin (**8Aa**, 96.4 mg, 60% yield).

4-Phenyl-3-(phenylethynyl)coumarin (8Aa): Yield: 96.4 mg (60%); yellow solid; Mp: 159-160 °C; IR (neat) 2207, 1716, 1246, 1064 cm^{-1}; ^{1}H-NMR (400 MHz, $CDCl_3$): δ = 7.19-7.33 (m, 7H), 7.42 (dd, 1H, *J* = 8.4, 0.9 Hz), 7.48-7.61 (m, 6H); ^{13}C-NMR (100 MHz, $CDCl_3$): δ = 83.7, 98.7, 111.1, 117.0, 119.7, 122.4, 124.5, 127.6, 128.2, 128.4, 128.8, 129.1, 129.4, 131.7, 132.0, 134.3, 152.8, 156.2, 159.5; HRMS (APCI) Calcd for

$C_{23}H_{15}O_2$ [M+H]$^+$ = 323.1067, Found = 323.1064.

Preparation of 3,4-diphenylcoumarin (9Aa)

To a mixture of 3-bromo-4-phenylcoumarin (**3Aa**, 0.5 mmol, 150.6 mg) and $PhB(OH)_2$ (1.0 mmol, 121.9 mg) in DMF (10 mL) was added $PdCl_2(PPh_3)_2$ (0.025 mmol, 17.5 mg). Under the argon atmosphere, the obtained mixture was stirred for 30 min at room temperature. Then, K_2CO_3 (1.0 mmol, 138.2 mg) in H_2O (2 mL) was added to the mixture, and the obtained mixture was stirred for 1.5 h at 60 °C. Water (5 mL) was added to the reaction mixture, and the product was extracted with CH_2Cl_2 (15 mL × 3), and washed with brine (15 mL × 2). The organic layer was dried over Na_2SO_4. After removal of the solvent under reduced pressure, the residue was purified by silica-gel column chromatography (eluent: *n*-hexane/EtOAc = 9:1) to give 3,4-diphenylcoumarin (**9Aa**, 113.7 mg, 76% yield).

3,4-Diphenylcoumarin (9Aa)[1]: Yield: 113.7 mg (76%); white solid; Mp: 223-224 °C; IR (neat) 1711, 1297, 1038 cm^{-1}; ^{1}H-NMR (400 MHz, $CDCl_3$): δ = 7.10-7.25 (m, 9H), 7.28-7.35 (m, 3H), 7.44 (dd, 1H, *J* = 8.3, 0.7 Hz), 7.54 (td, 1H, *J* = 7.4, 2.0 Hz); ^{13}C-NMR (100 MHz, $CDCl_3$): δ = 116.7, 120.4, 124.1, 126.9, 127.6, 127.7, 127.8, 128.2, 128.3, 129.3, 130.5, 131.4, 133.8, 134.4, 151.6, 153.2, 161.3; HRMS (APCI) Calcd for $C_{21}H_{15}O_2$ [M+H]$^+$ = 299.1067, Found = 299.1065.

References

1. Zhang, L.; Meng, T.; Fan, T.; Wu, J. *J. Org. Chem*. **2007**, *72*, 7279-7286.
2. Qiu, G.; Liu, T.; Ding, Q. *Org. Chem. Front.* **2016**, *3*, 510-515.
3. Sasaki, T.; Miyagi, K.; Moriyama, K.; Togo, H. *Org. Lett*. **2016**, *18*, 944-947.

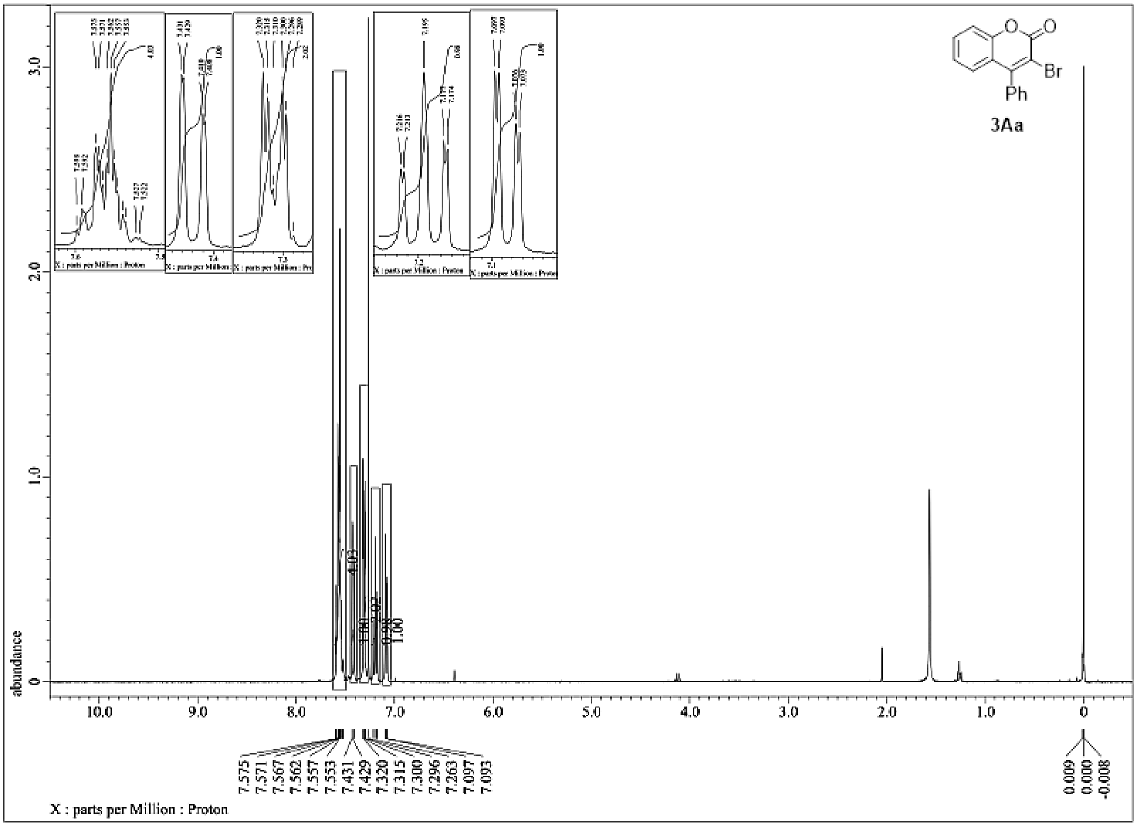

3Aa
Br
Ph
abundance
X : parts per Million : Proton
7.575
7.571
7.567
7.562
7.557
7.553
7.431
7.429
7.320
7.315
7.300
7.296
7.263
7.097
7.093
0.009
0.000
-0.008
4.03
1.00

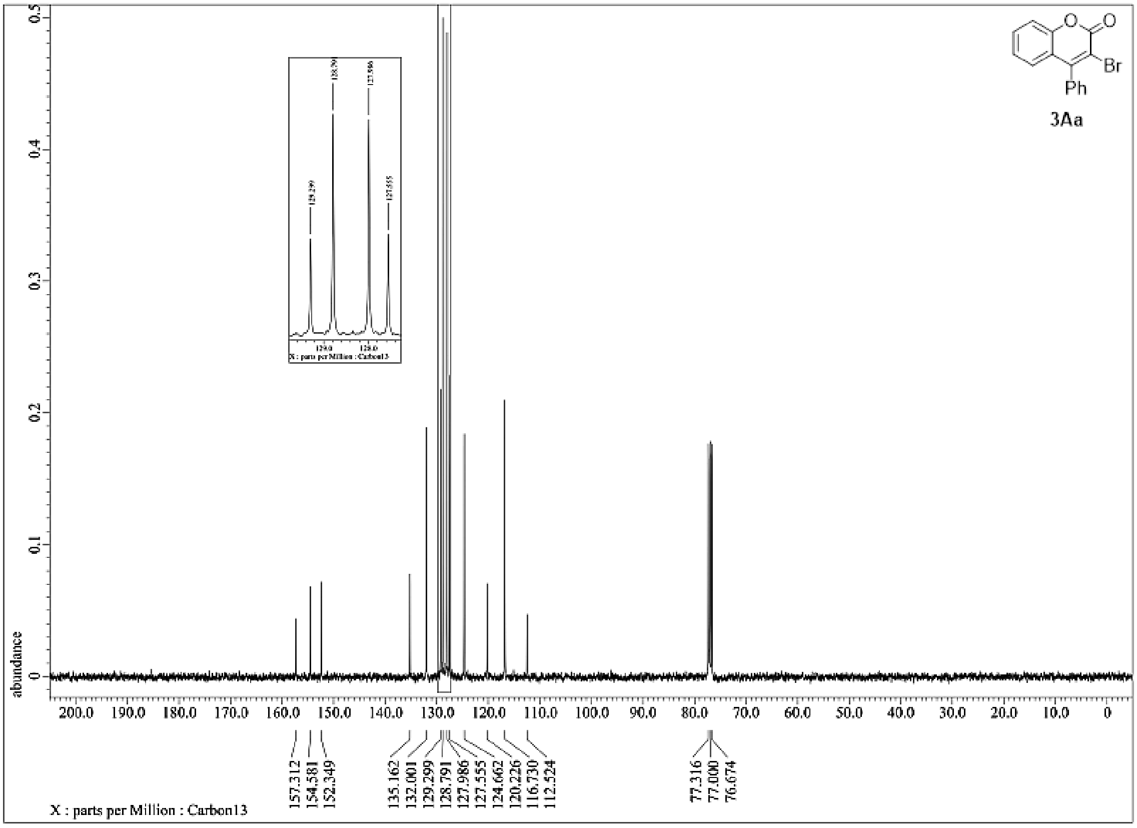

3Aa
Br
Ph
abundance
X : parts per Million : Carbon13
157.312
154.581
152.349
135.162
132.001
129.299
128.791
127.986
127.555
124.662
120.226
116.730
112.524
77.316
77.000
76.674

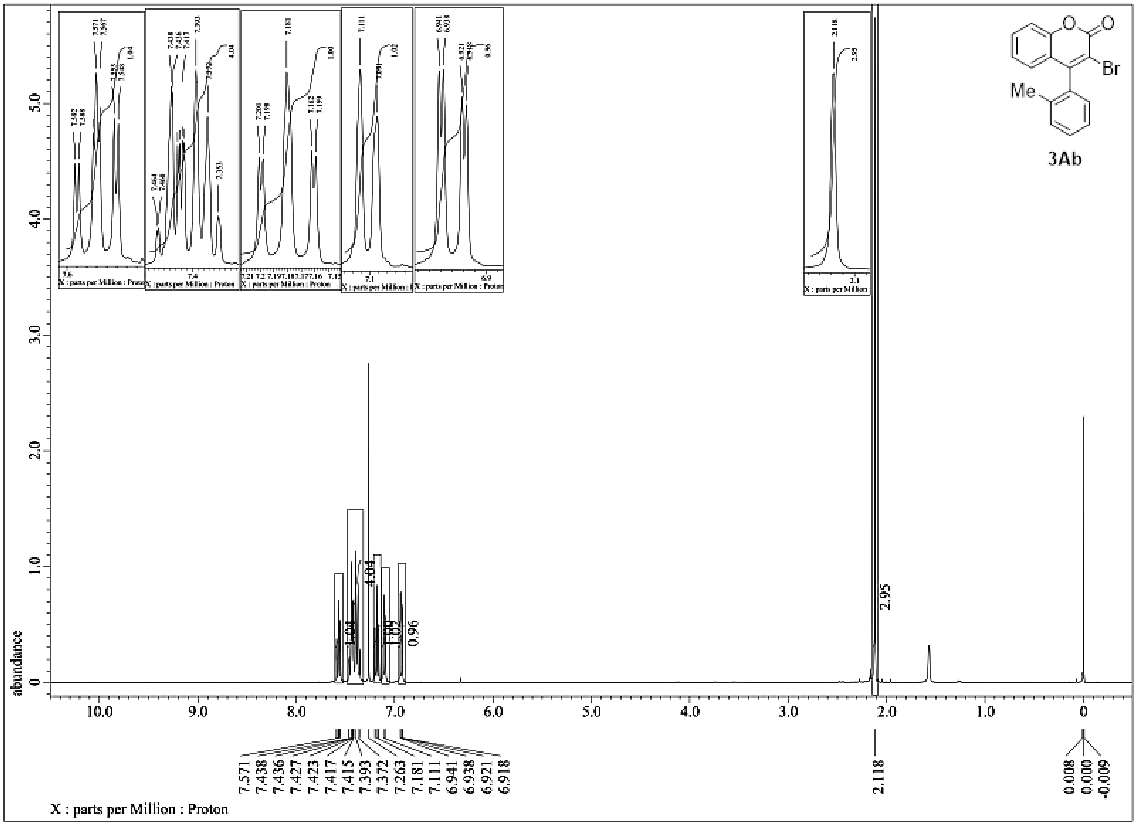

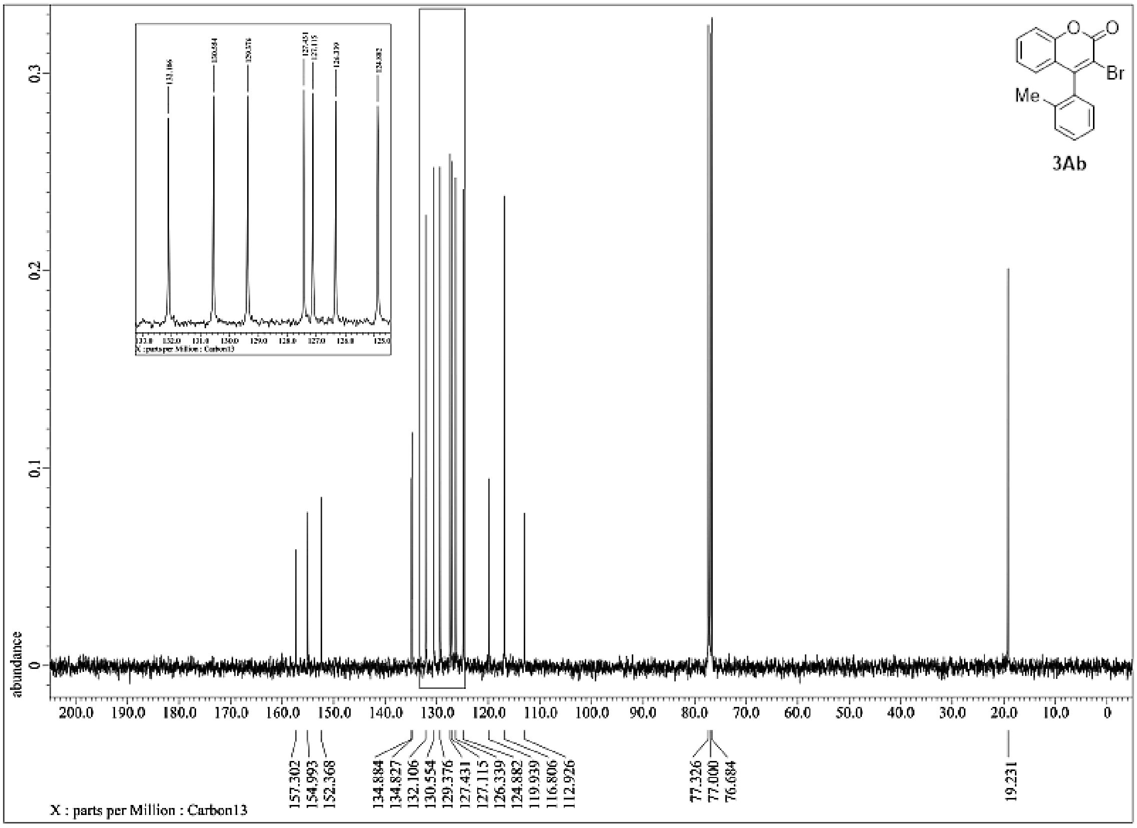

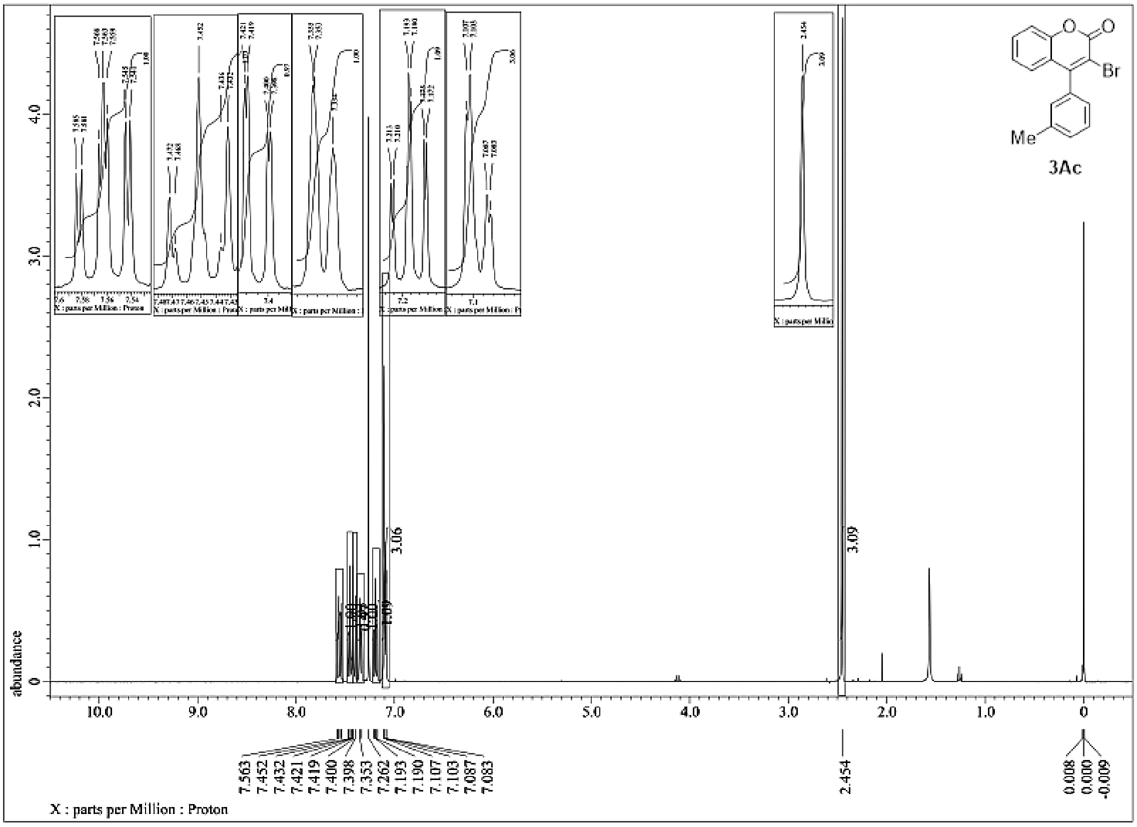
abundance
X : parts per Million : Proton
3Ac
Br
Me
3.06
3.09
2.454
7.563
7.452
7.432
7.421
7.419
7.400
7.398
7.353
7.262
7.193
7.190
7.107
7.103
7.087
7.083
0.008
0.000
-0.009

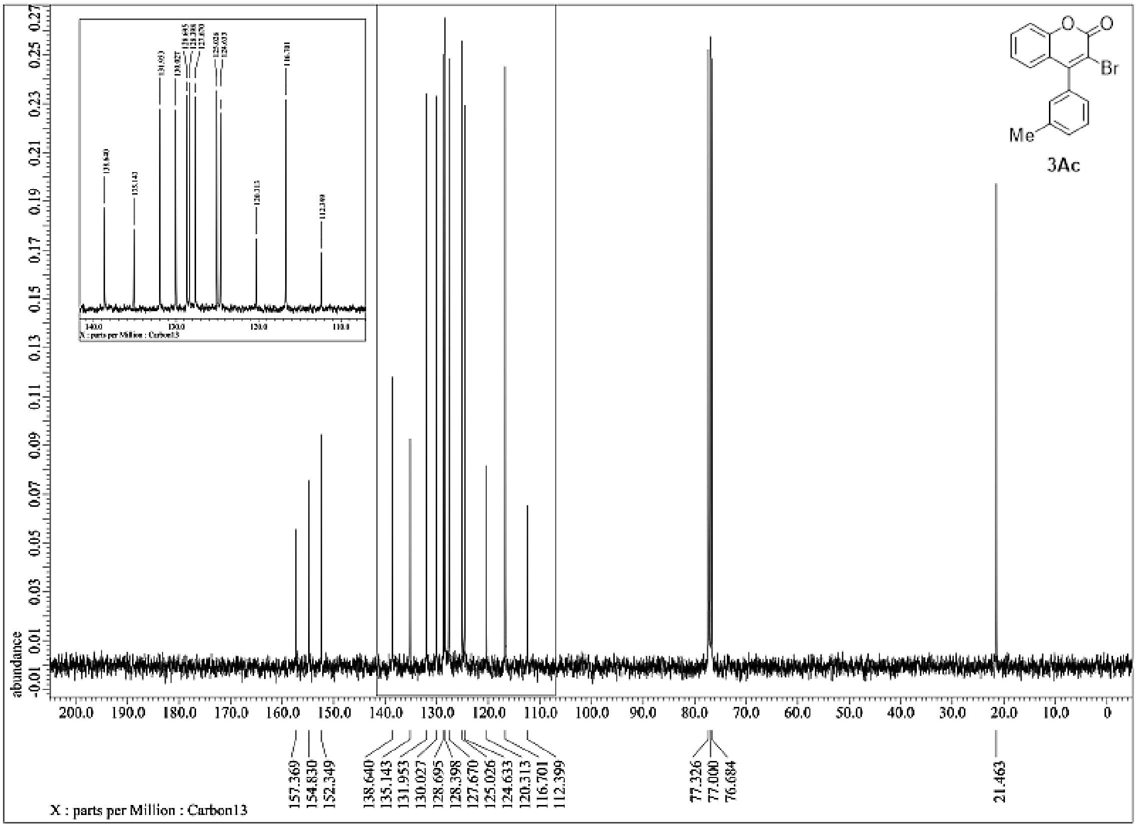
abundance
X : parts per Million : Carbon13
3Ac
Br
Me
157.369
154.830
152.349
138.640
135.143
131.953
130.027
128.695
128.398
127.670
125.026
124.633
120.313
116.701
112.399
77.326
77.000
76.684
21.463

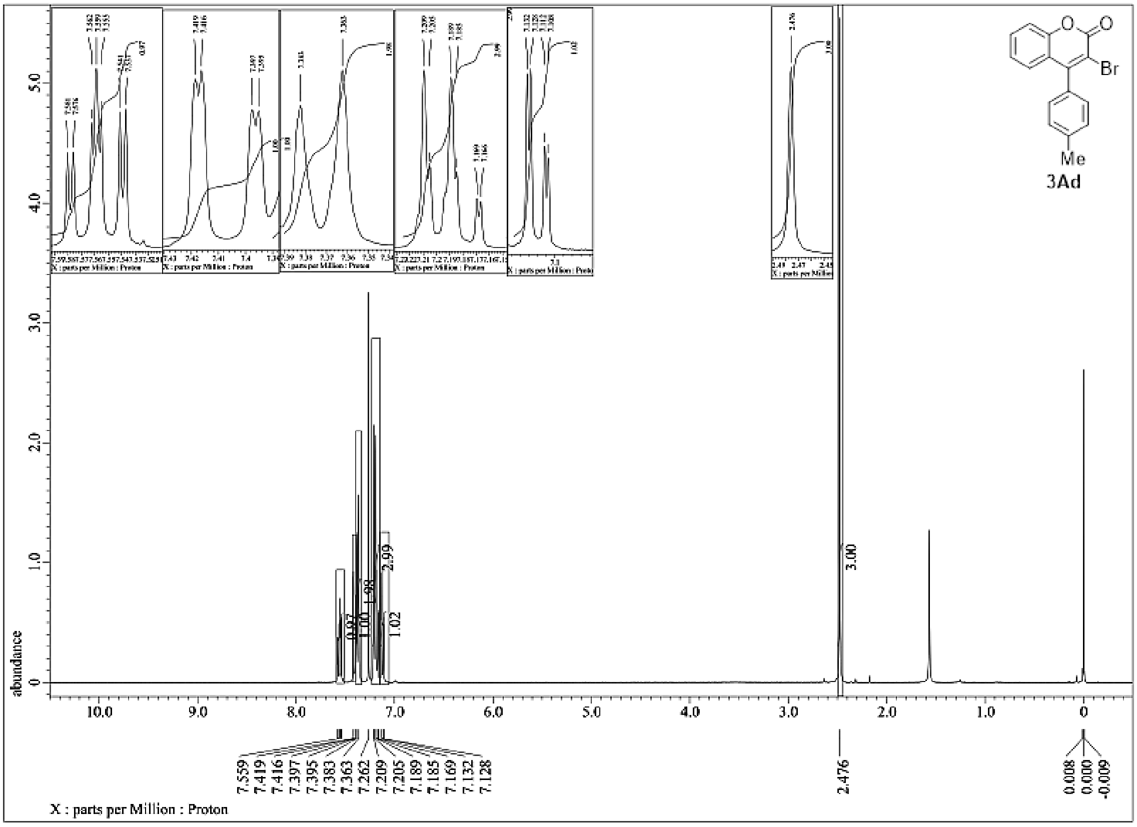
3Ad
Br
Me
X : parts per Million : Proton
abundance
3.00
2.476
0.008
0.000
-0.009
7.559
7.419
7.416
7.397
7.395
7.383
7.363
7.262
7.209
7.205
7.189
7.185
7.169
7.132
7.128

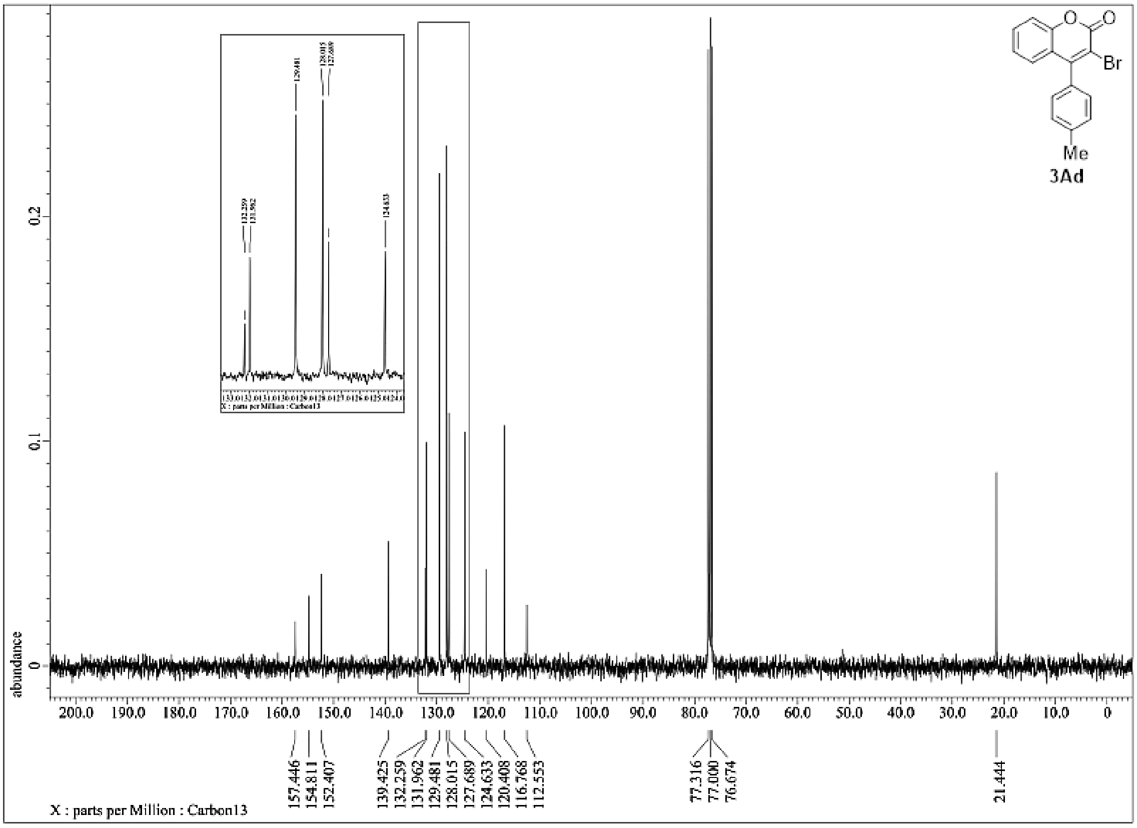
3Ad
Br
Me
X : parts per Million : Carbon13
abundance
157.446
154.811
152.407
139.425
132.259
131.962
129.481
128.015
127.689
124.633
120.408
116.768
112.553
77.316
77.000
76.674
21.444

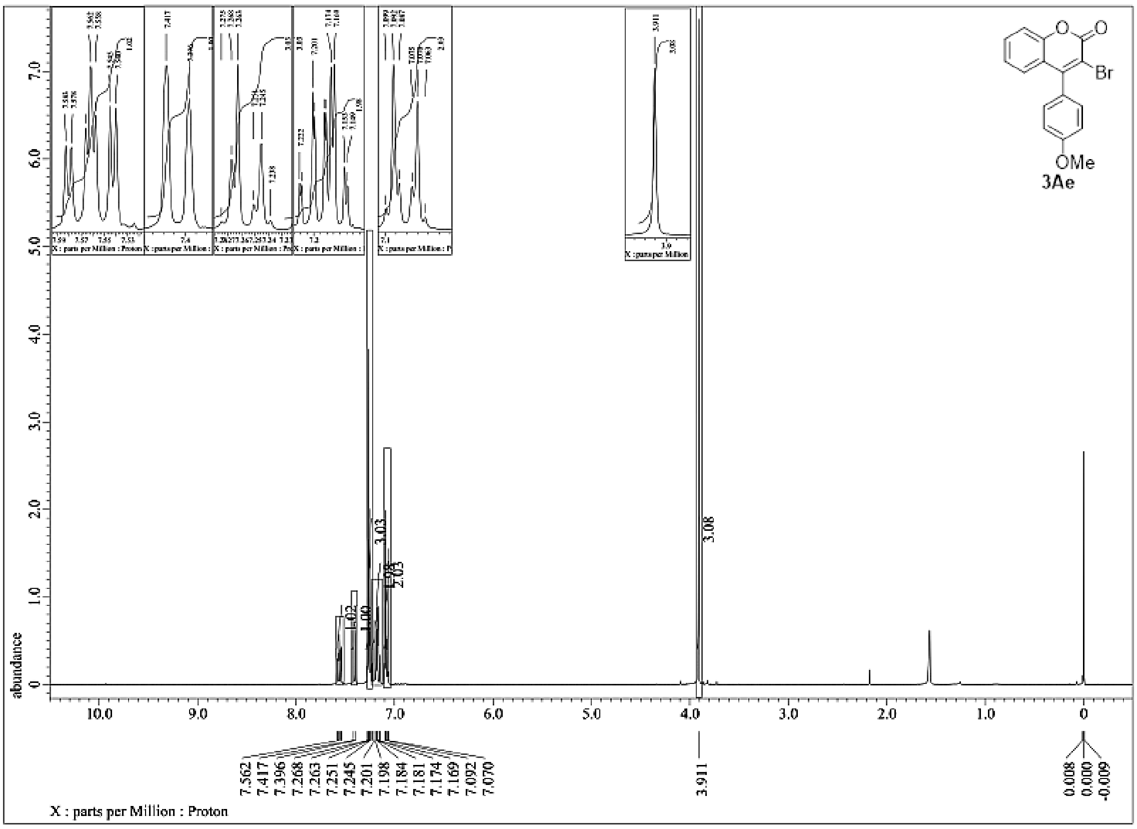

3Ae
OMe
Br
abundance
X : parts per Million : Proton
7.562
7.417
7.396
7.268
7.263
7.251
7.245
7.201
7.198
7.184
7.181
7.174
7.169
7.092
7.070
3.911
0.008
0.000
-0.009
3.08
3.03
2.03

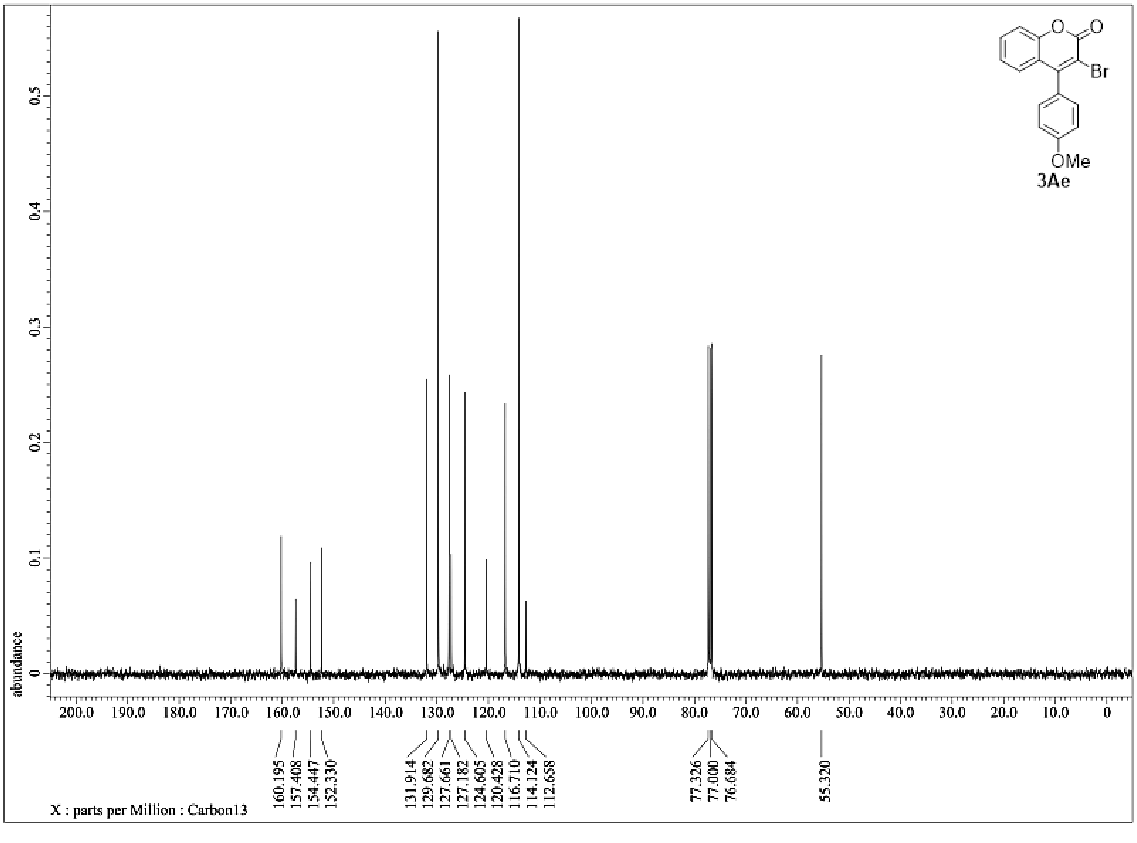

3Ae
OMe
Br
abundance
X : parts per Million : Carbon13
160.195
157.408
154.447
152.330
131.914
129.682
127.661
127.182
124.605
120.428
116.710
114.124
112.658
77.326
77.000
76.684
55.320

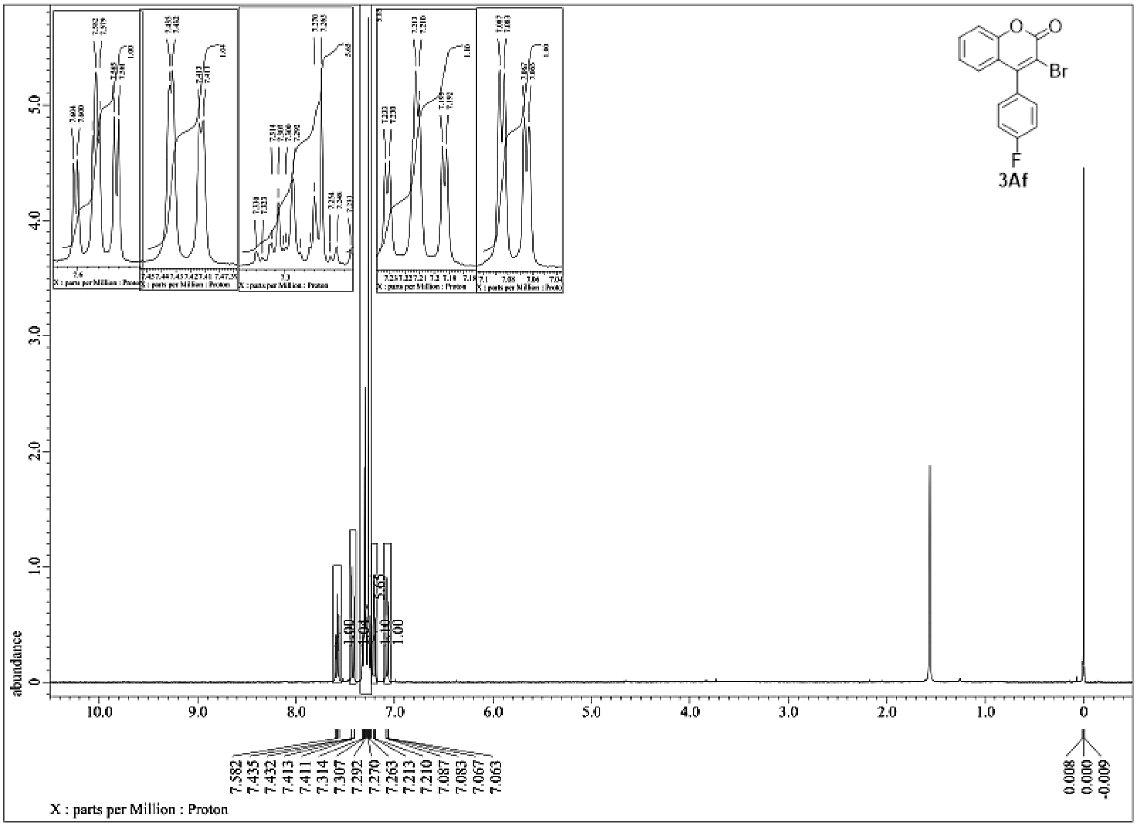

3Af
Br
F
abundance
X : parts per Million : Proton
7.582
7.435
7.432
7.413
7.411
7.314
7.307
7.292
7.270
7.263
7.213
7.210
7.087
7.083
7.067
7.063
0.008
0.000
-0.009
1.00
1.04
5.65
1.10
1.00

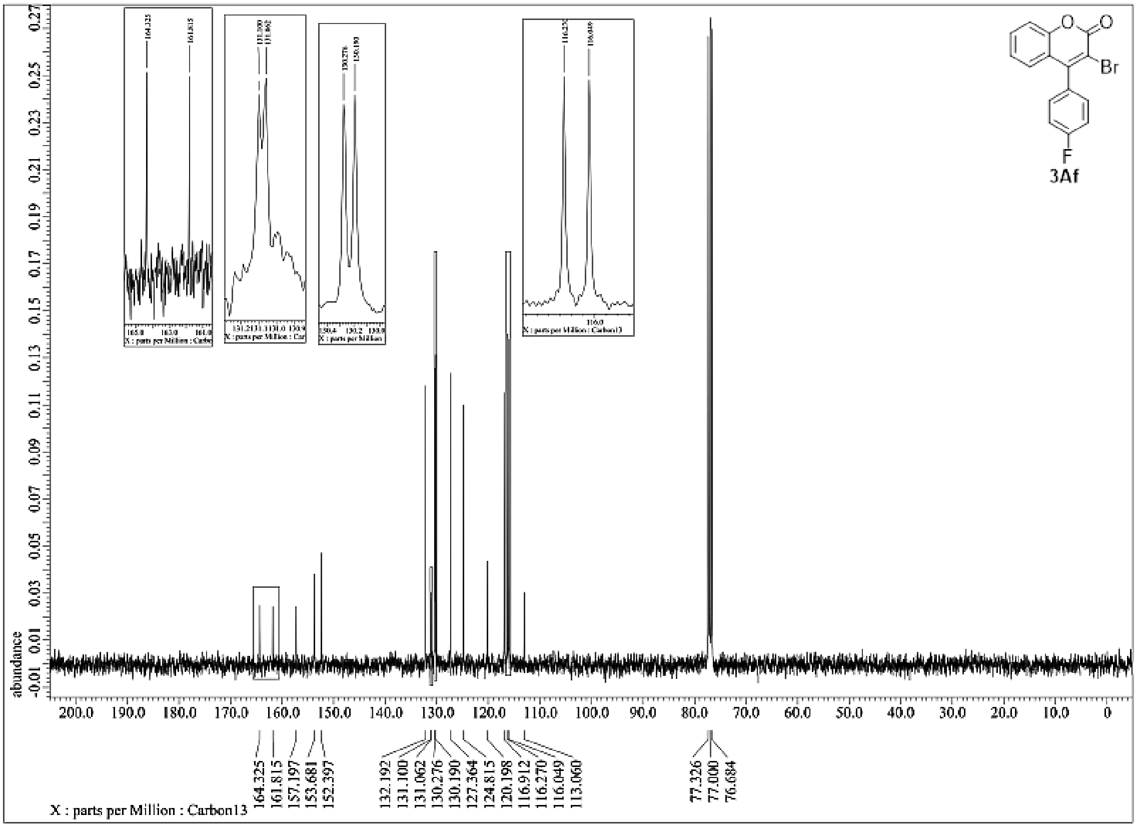

3Af
Br
F
abundance
X : parts per Million : Carbon13
164.325
161.815
157.197
153.681
152.397
132.192
131.100
131.062
130.276
130.190
127.364
124.815
120.198
116.912
116.270
116.049
113.060
77.326
77.000
76.684

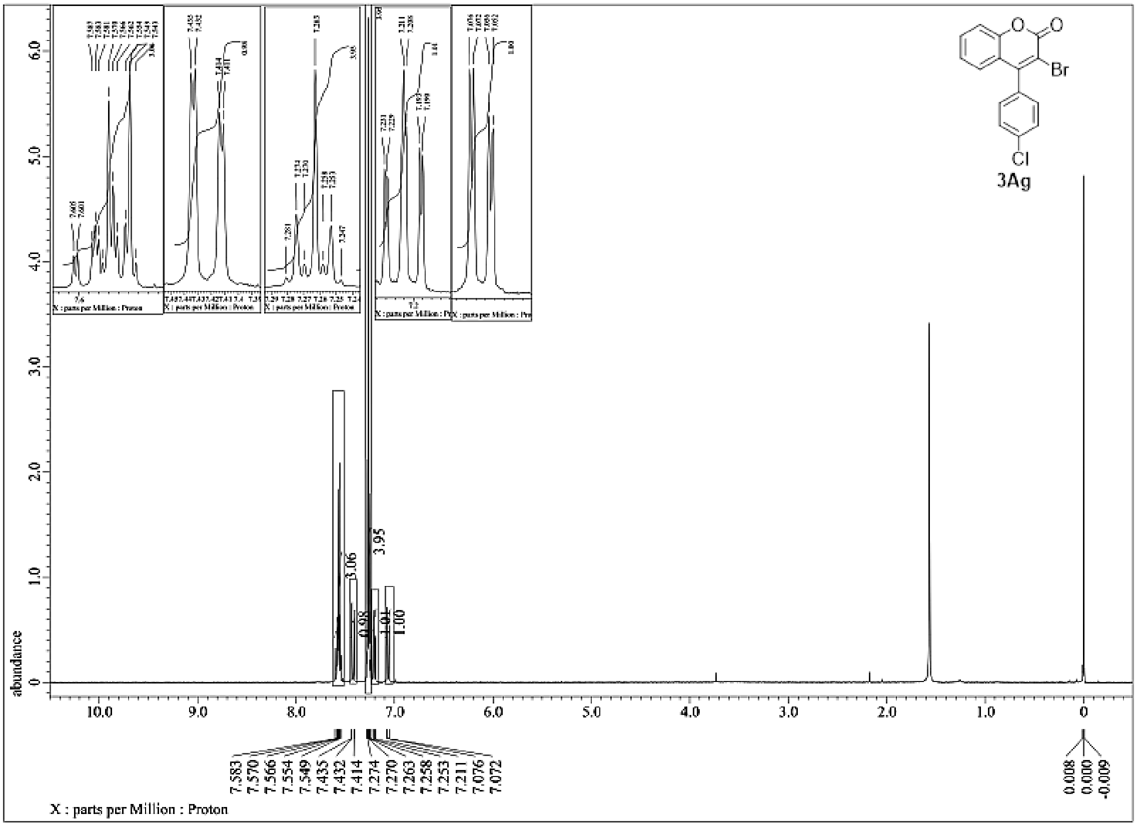
3Ag
Br
Cl
abundance
3.06
3.95
0.98
1.01
1.00
7.583
7.570
7.566
7.554
7.549
7.435
7.432
7.414
7.274
7.270
7.263
7.258
7.253
7.211
7.076
7.072
0.008
0.000
-0.009
X : parts per Million : Proton

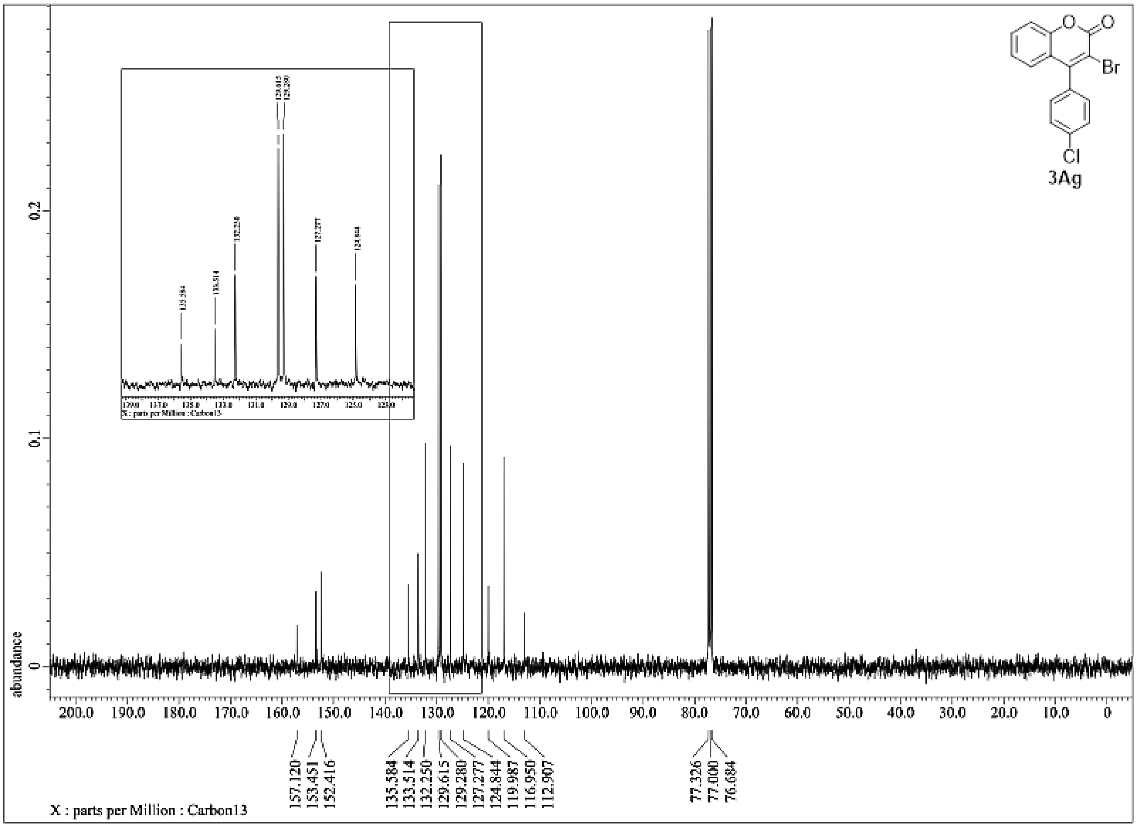
3Ag
Br
Cl
abundance
157.120
153.451
152.416
135.584
133.514
132.250
129.615
129.280
127.277
124.844
119.987
116.950
112.907
77.326
77.000
76.684
X : parts per Million : Carbon13

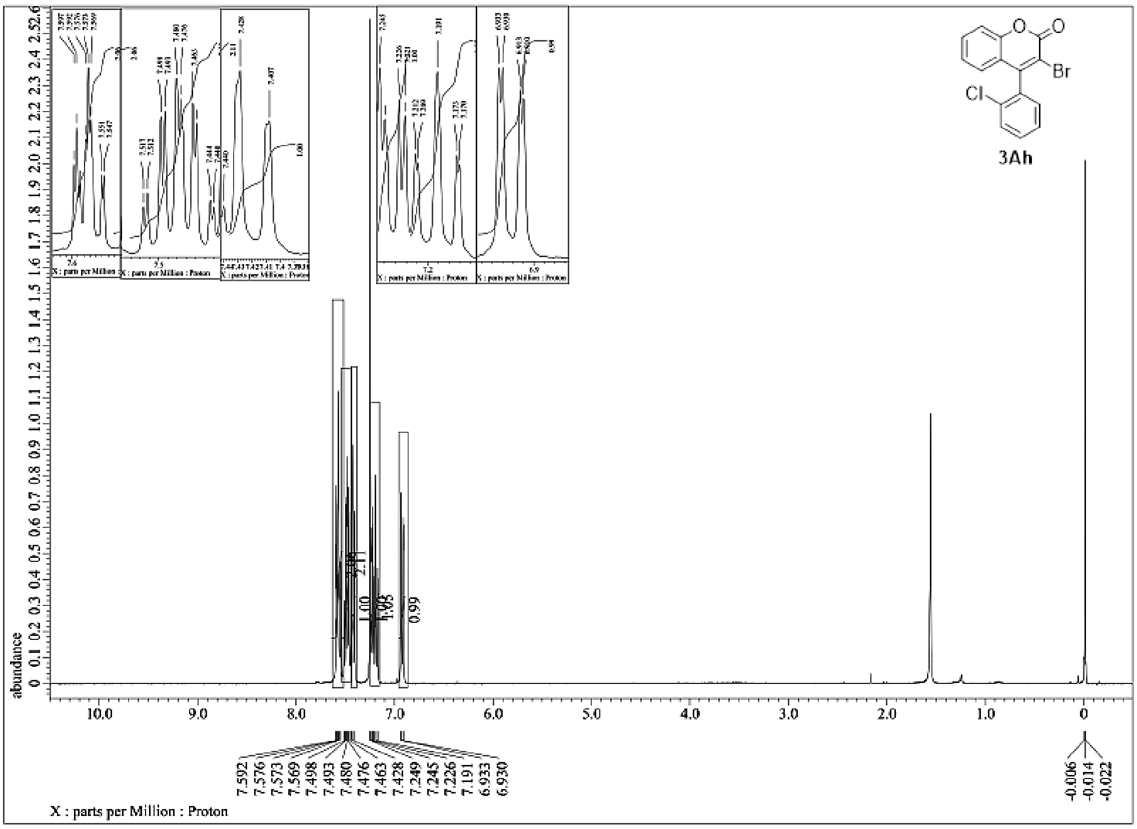
3Ah
X : parts per Million : Proton
abundance
7.592
7.576
7.573
7.569
7.498
7.493
7.480
7.476
7.463
7.428
7.249
7.245
7.226
7.191
6.933
6.930
-0.006
-0.014
-0.022

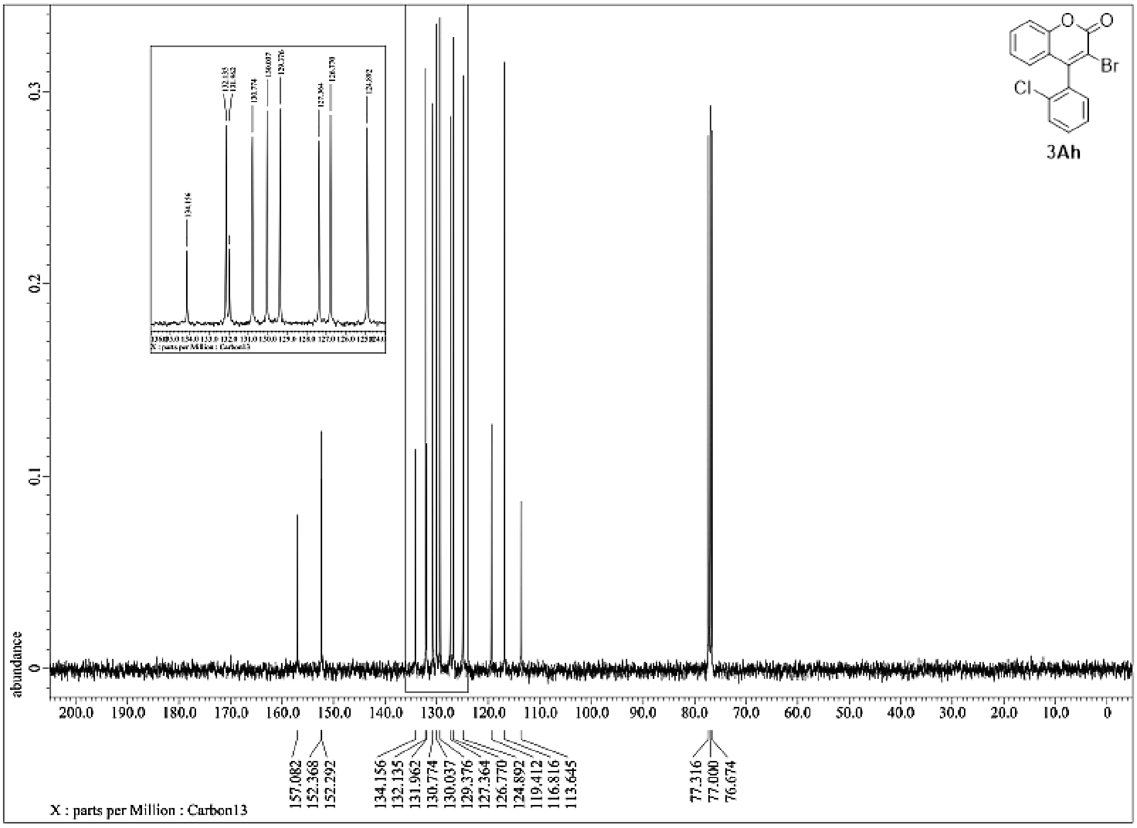
3Ah
X : parts per Million : Carbon13
abundance
157.082
152.368
152.292
134.156
132.135
131.962
130.774
130.037
129.376
127.364
126.770
124.892
119.412
116.816
113.645
77.316
77.000
76.674

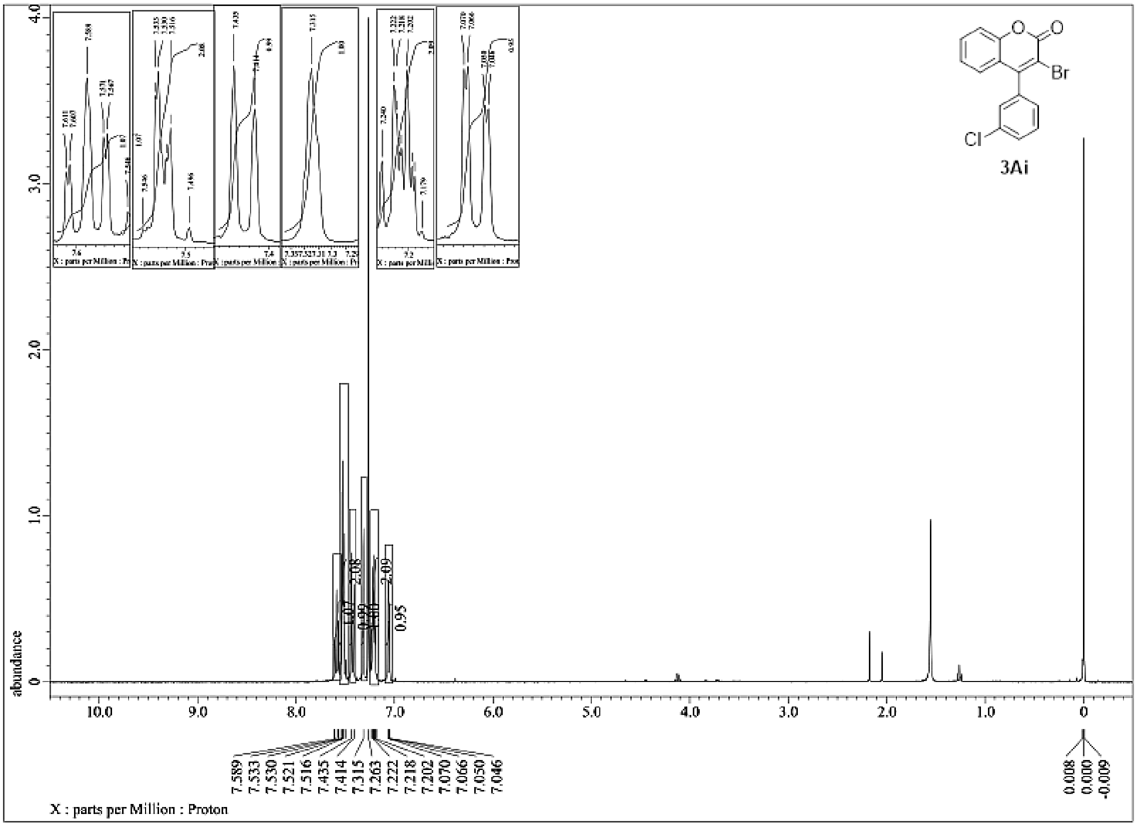
3Ai
Br
Cl
abundance
X : parts per Million : Proton
7.589
7.533
7.530
7.521
7.516
7.435
7.414
7.315
7.263
7.222
7.218
7.202
7.070
7.066
7.050
7.046
0.008
0.000
-0.009

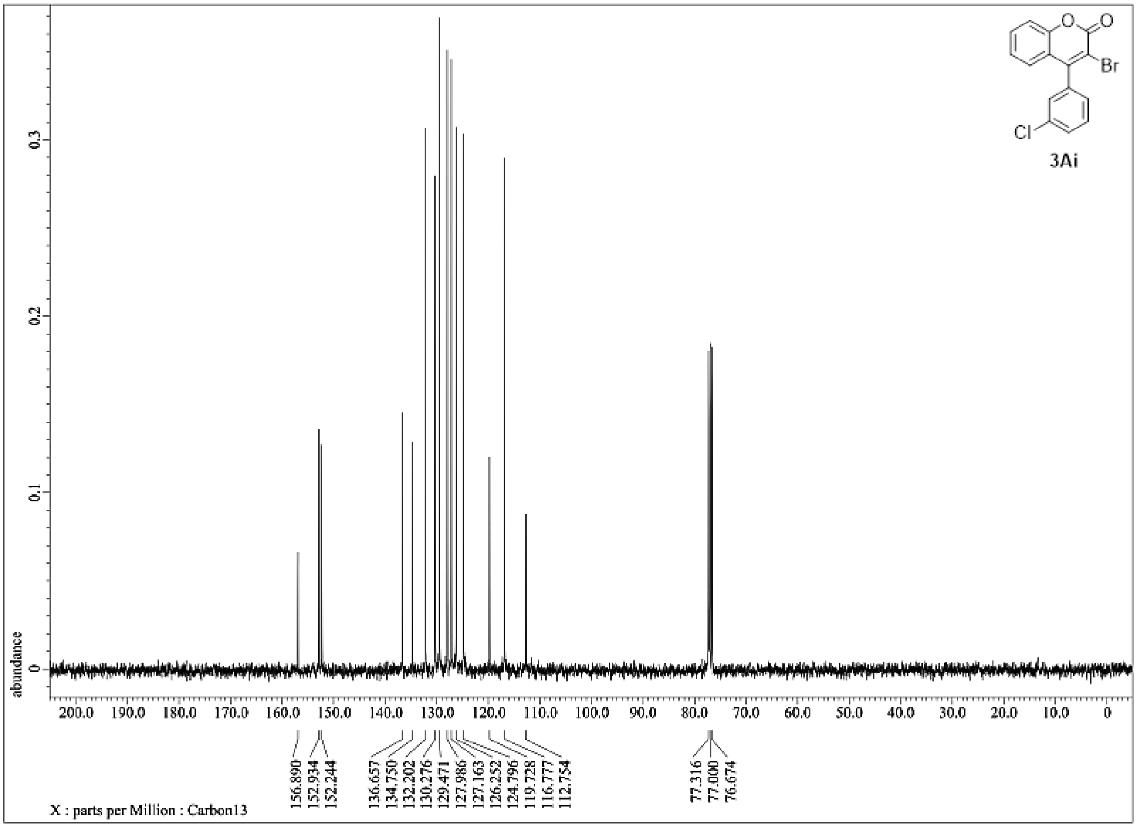
3Ai
Br
Cl
abundance
X : parts per Million : Carbon13
156.890
152.934
152.244
136.657
134.750
132.202
130.276
129.471
127.986
127.163
126.252
124.796
119.728
116.777
112.754
77.316
77.000
76.674

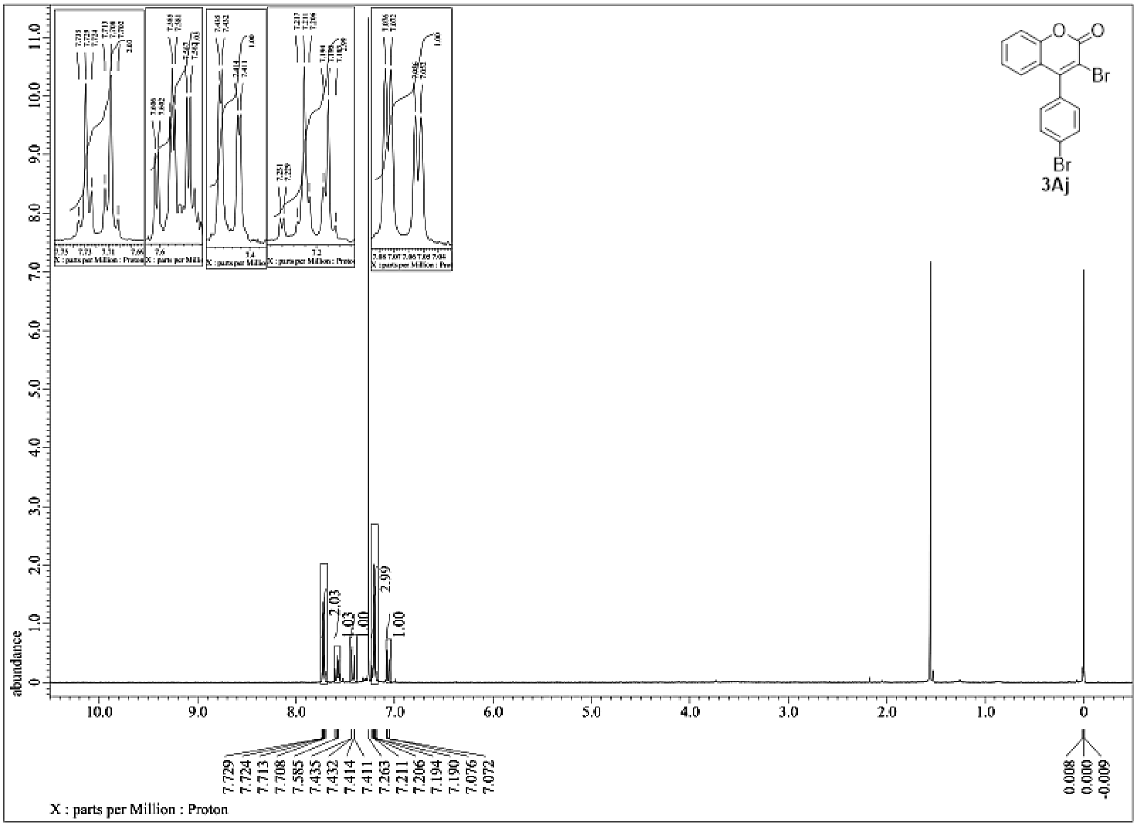
Br
Br
3Aj
abundance
X : parts per Million : Proton
7.729
7.724
7.713
7.708
7.585
7.435
7.432
7.414
7.411
7.263
7.211
7.206
7.194
7.190
7.076
7.072
0.008
0.000
-0.009
2.03
1.03
1.00
2.99
1.00

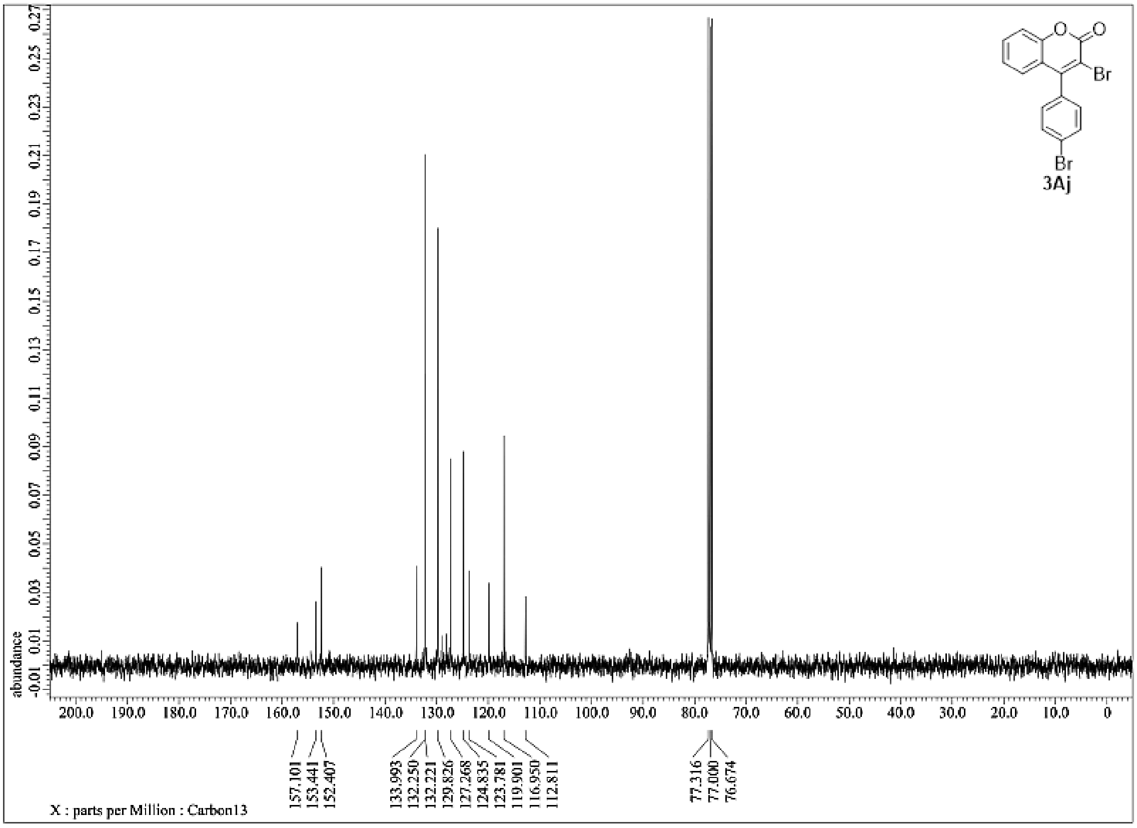
Br
Br
3Aj
abundance
X : parts per Million : Carbon13
157.101
153.441
152.407
133.993
132.250
132.221
129.826
127.268
124.835
123.781
119.901
116.950
112.811
77.316
77.000
76.674

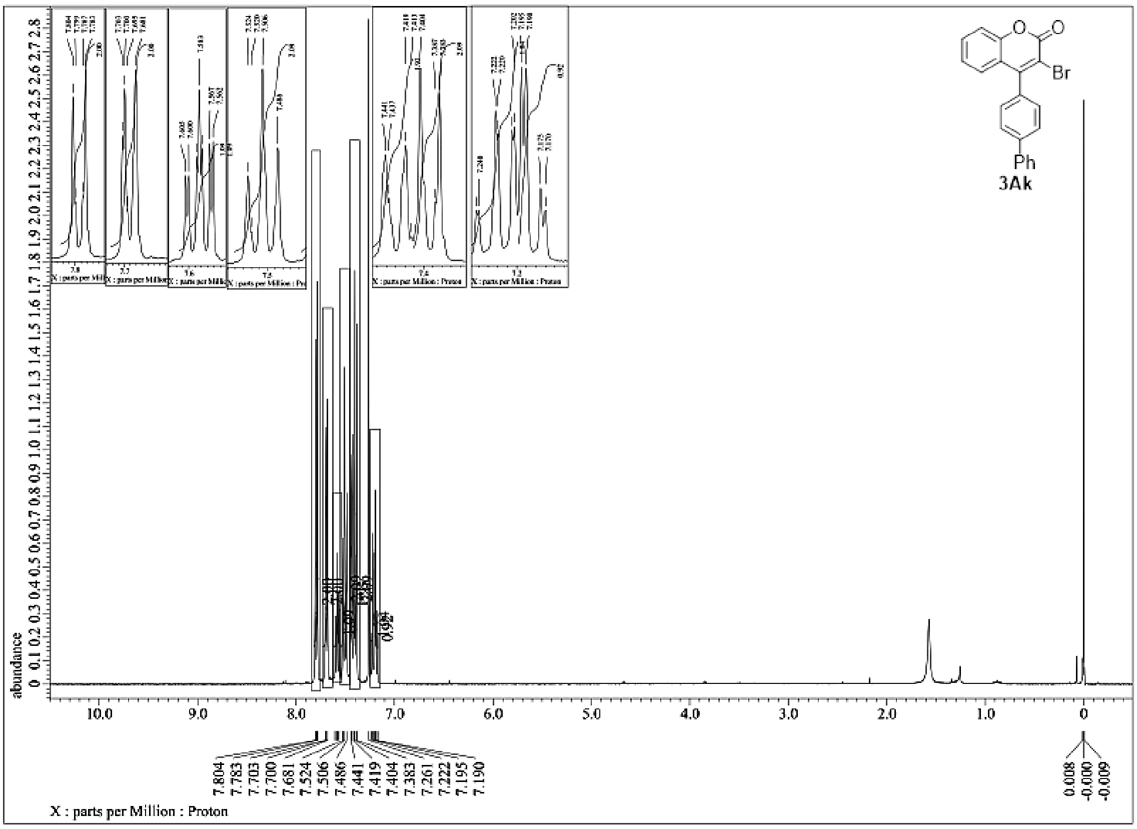
3Ak
Br
Ph
abundance
7.804
7.783
7.703
7.700
7.681
7.524
7.506
7.486
7.441
7.419
7.404
7.383
7.261
7.222
7.195
7.190
0.008
0.000
-0.009
X : parts per Million : Proton

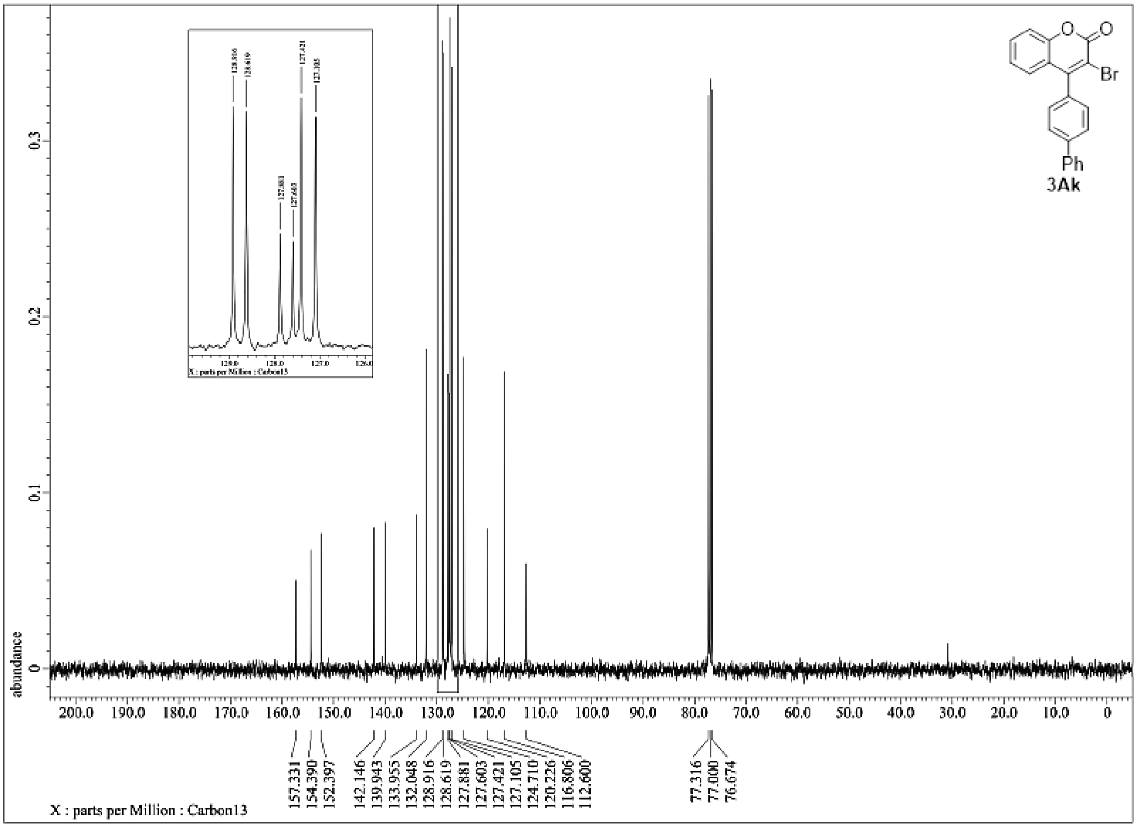
3Ak
Br
Ph
abundance
157.331
154.390
152.397
142.146
139.943
133.955
132.048
128.916
128.619
127.881
127.603
127.421
127.105
124.710
120.226
116.806
112.600
77.316
77.000
76.674
X : parts per Million : Carbon13

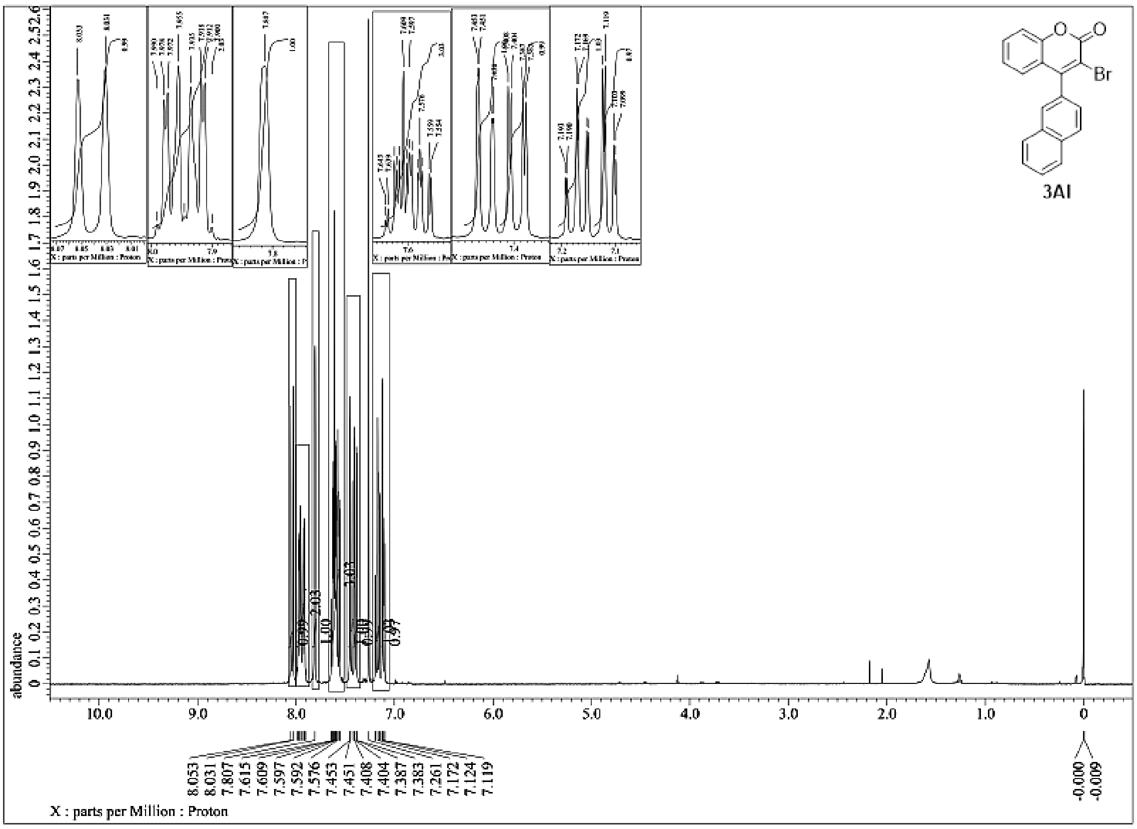
3AI
Br
abundance
X : parts per Million : Proton
8.053
8.031
7.807
7.615
7.609
7.597
7.592
7.576
7.453
7.451
7.408
7.404
7.387
7.383
7.261
7.172
7.124
7.119
-0.000
-0.009

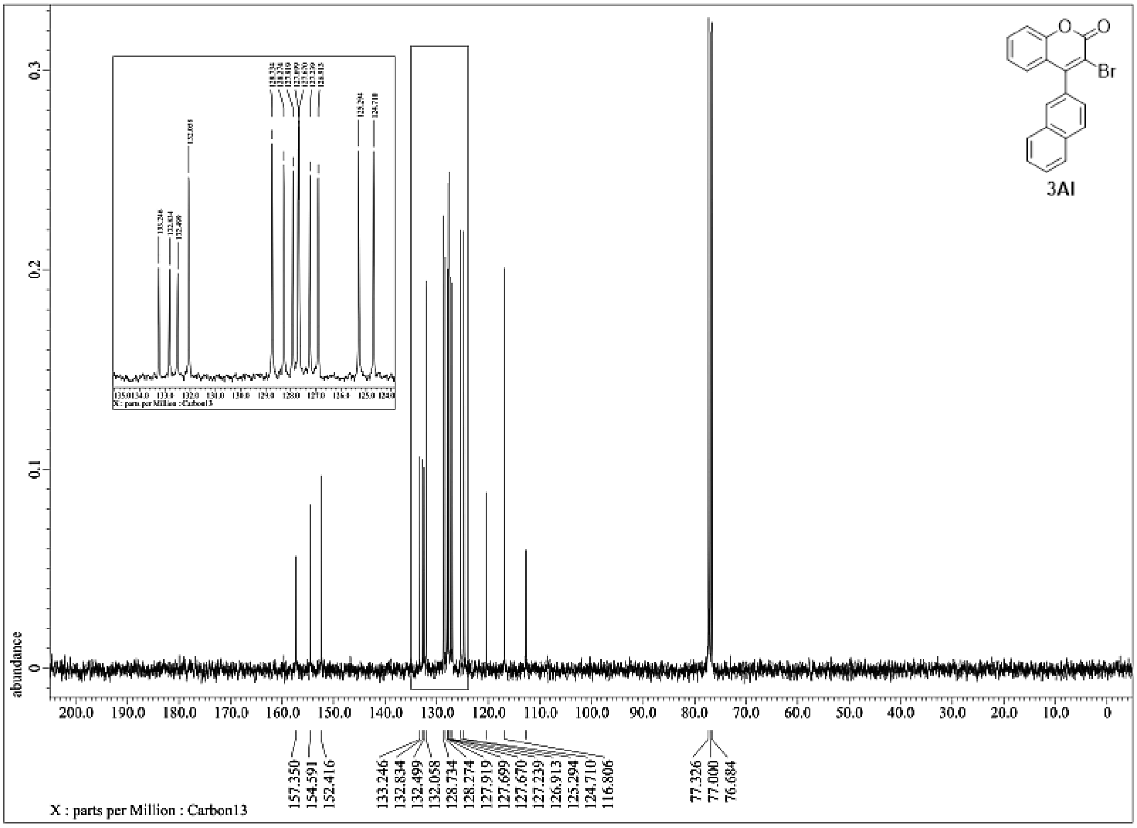
3AI
Br
abundance
X : parts per Million : Carbon13
157.350
154.591
152.416
133.246
132.834
132.499
132.058
128.734
128.274
127.919
127.699
127.670
127.239
126.913
125.294
124.710
116.806
77.326
77.000
76.684

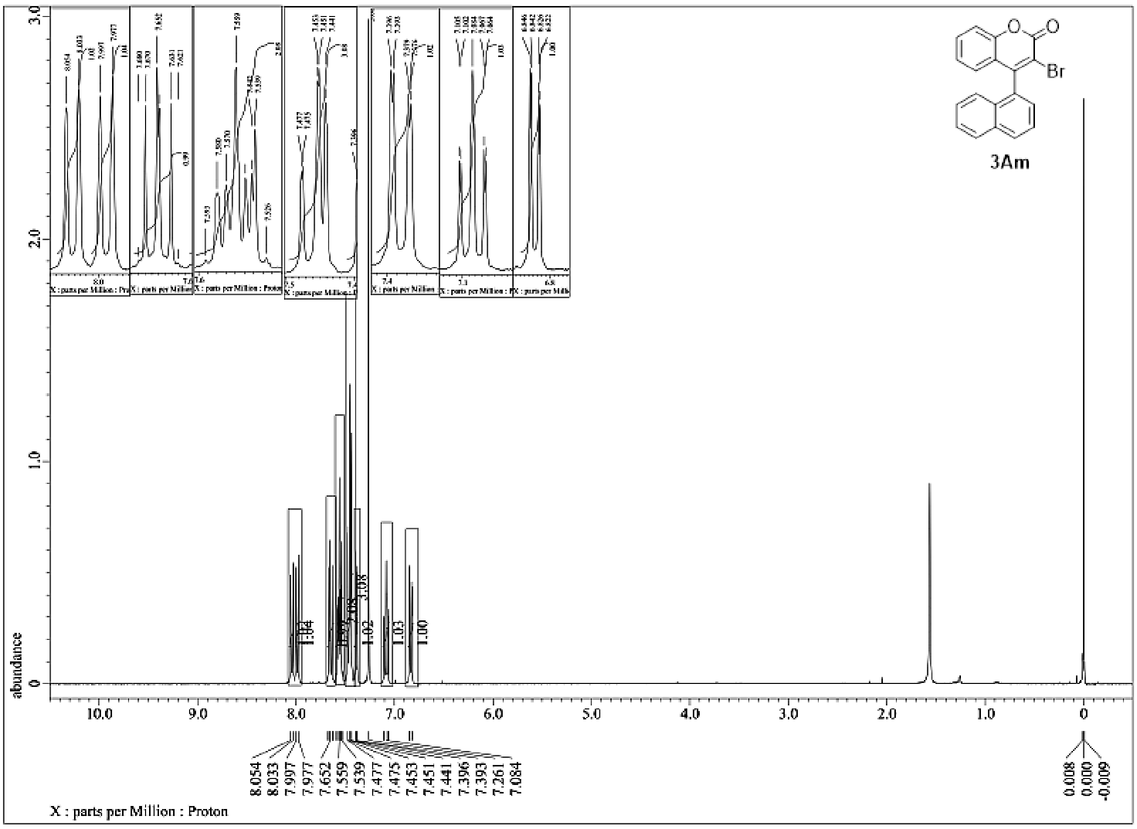

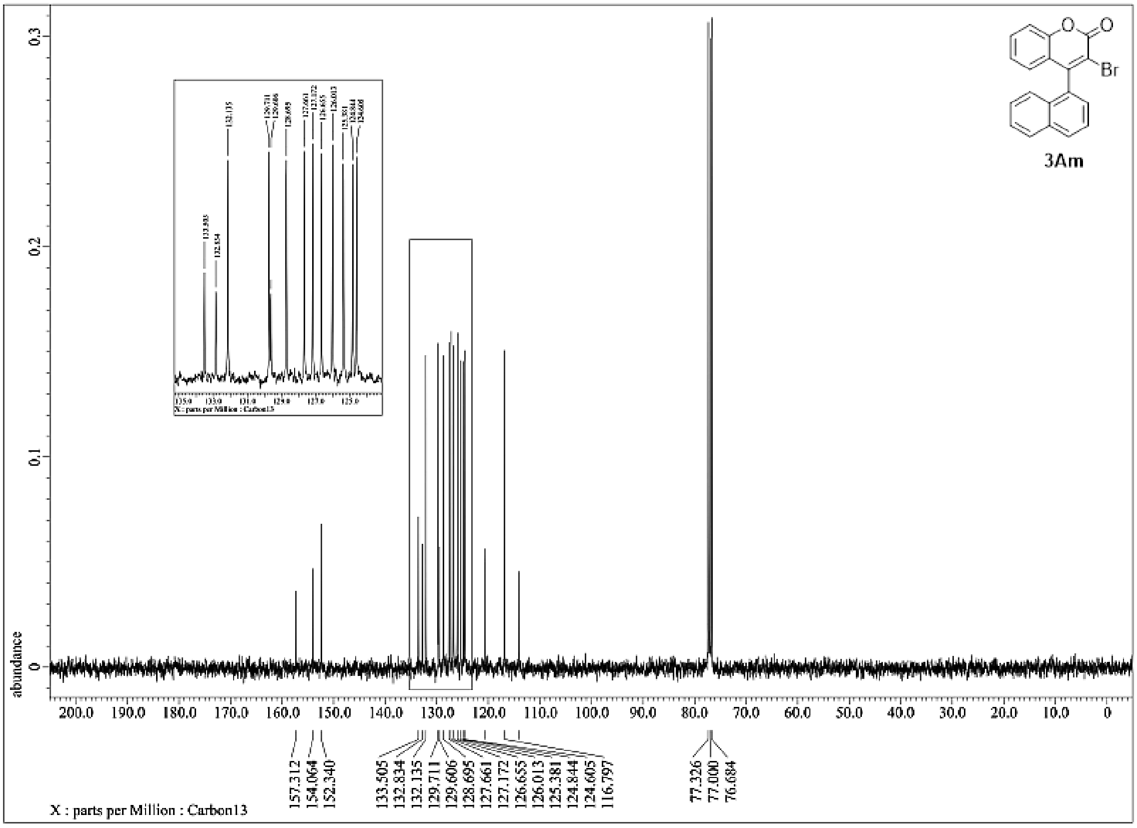

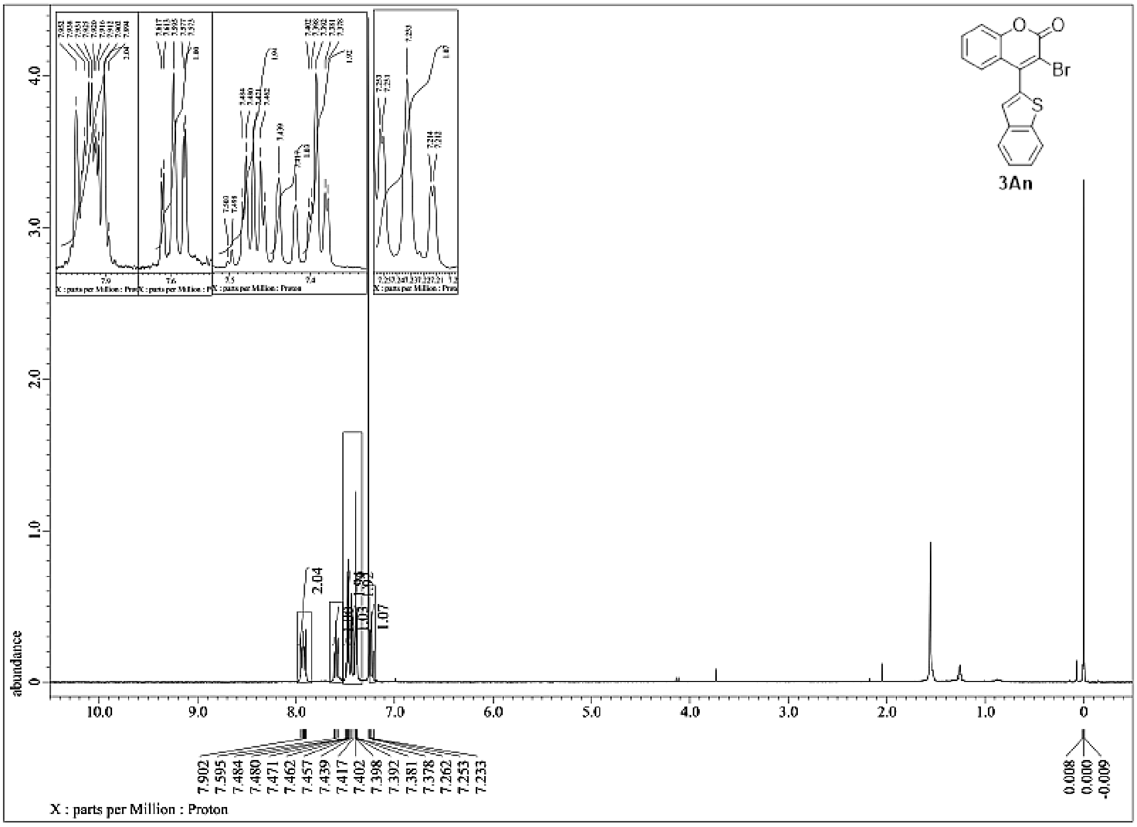
3An
abundance
X : parts per Million : Proton
7.902
7.595
7.484
7.480
7.471
7.462
7.457
7.439
7.417
7.402
7.398
7.392
7.381
7.378
7.262
7.253
7.233
0.008
0.000
-0.009
2.04
1.07

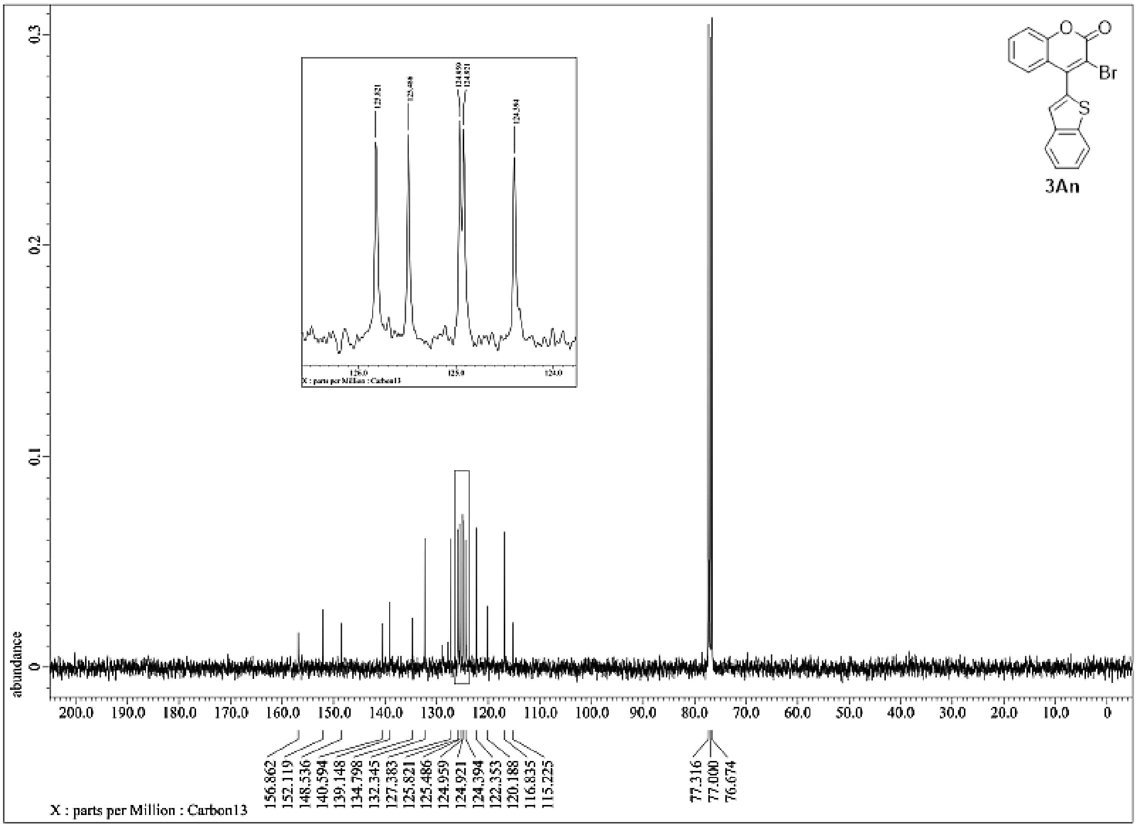
3An
abundance
X : parts per Million : Carbon13
156.862
152.119
148.536
140.594
139.148
134.798
132.345
127.383
125.821
125.486
124.959
124.921
124.394
122.353
120.188
116.835
115.225
77.316
77.000
76.674

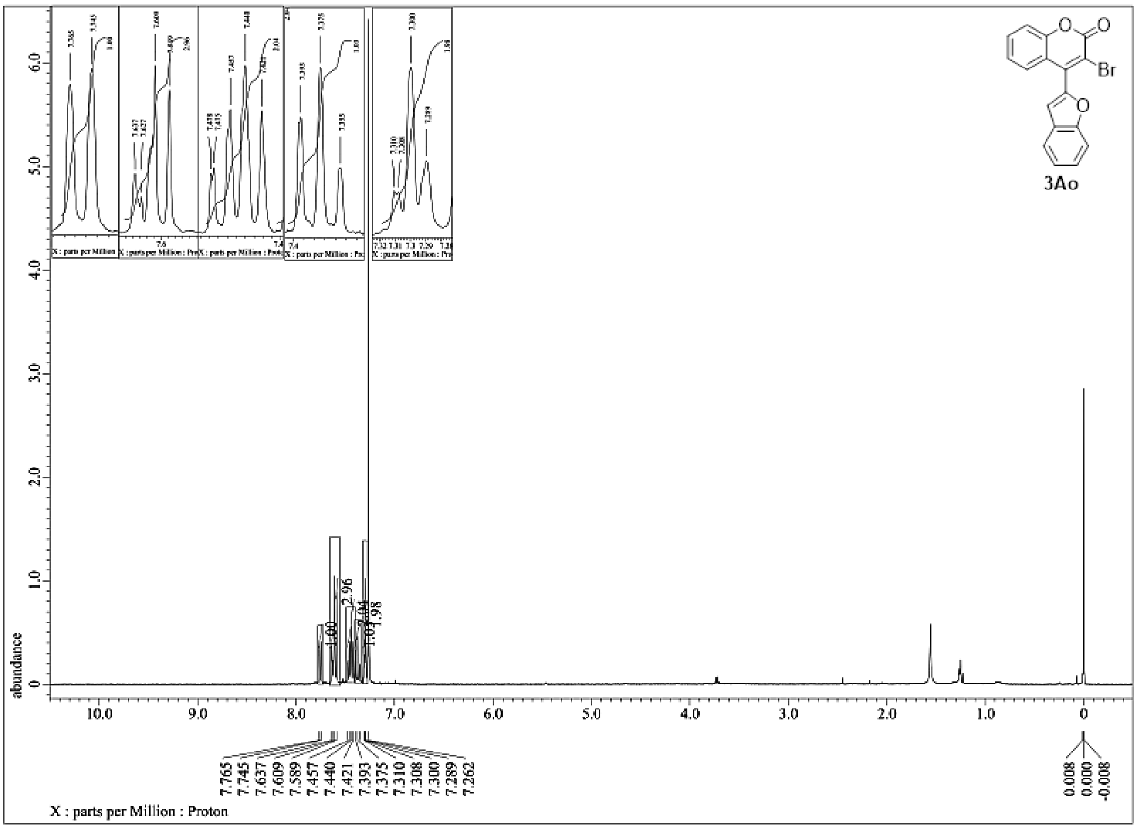
3Ao
abundance
X : parts per Million : Proton
7.765
7.745
7.637
7.609
7.589
7.457
7.440
7.421
7.393
7.375
7.310
7.308
7.300
7.289
7.262
0.008
0.000
-0.008

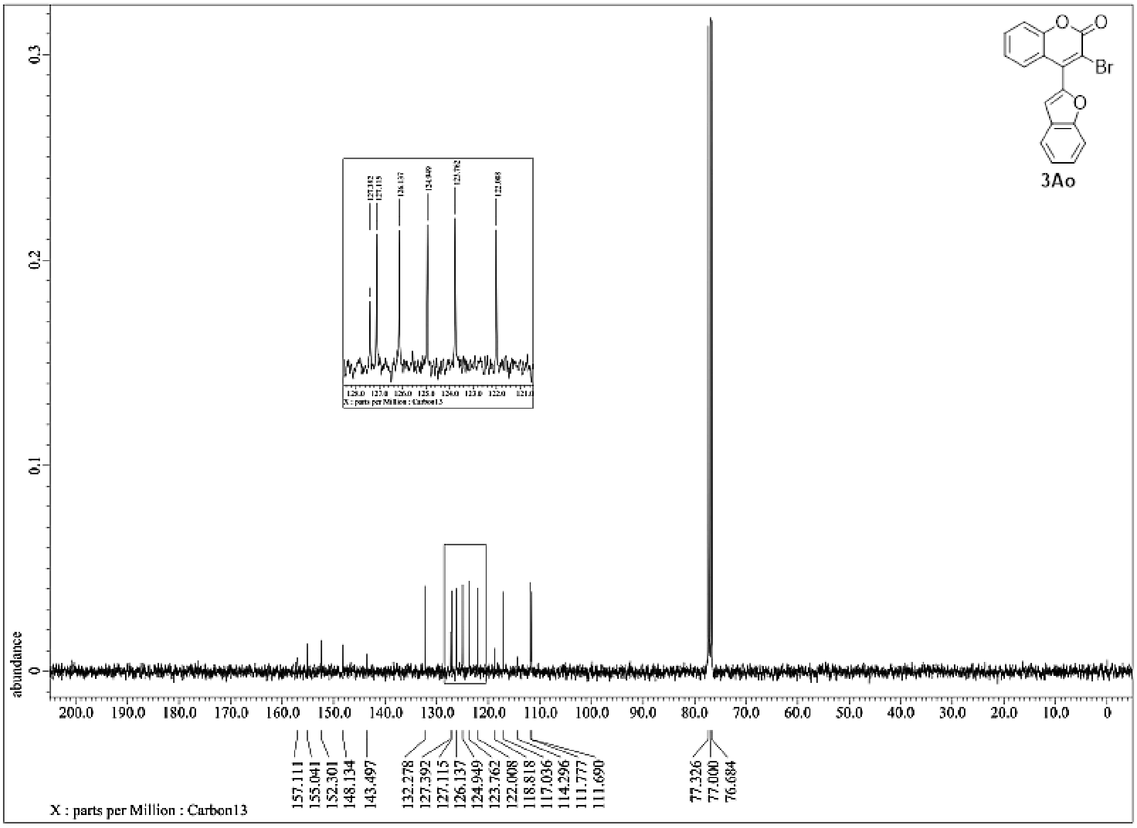
3Ao
abundance
X : parts per Million : Carbon13
157.111
155.041
152.301
148.134
143.497
132.278
127.392
127.115
126.137
124.949
123.762
122.008
118.818
117.036
114.296
111.777
111.690
77.326
77.000
76.684

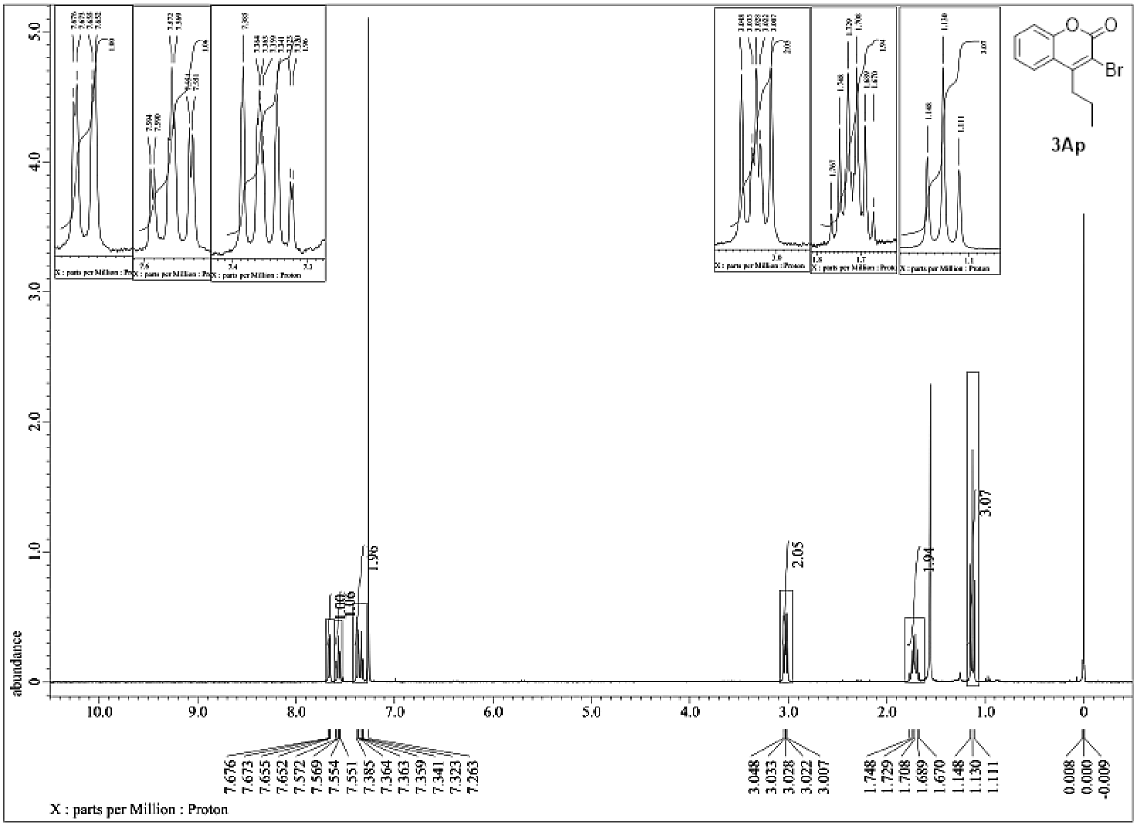
Br
3Ap
abundance
X : parts per Million : Proton
7.676
7.673
7.655
7.652
7.572
7.569
7.554
7.551
7.385
7.364
7.363
7.359
7.341
7.323
7.263
3.048
3.033
3.028
3.022
3.007
1.748
1.729
1.708
1.689
1.670
1.148
1.130
1.111
0.008
0.000
-0.009
1.96
2.05
1.94
3.07

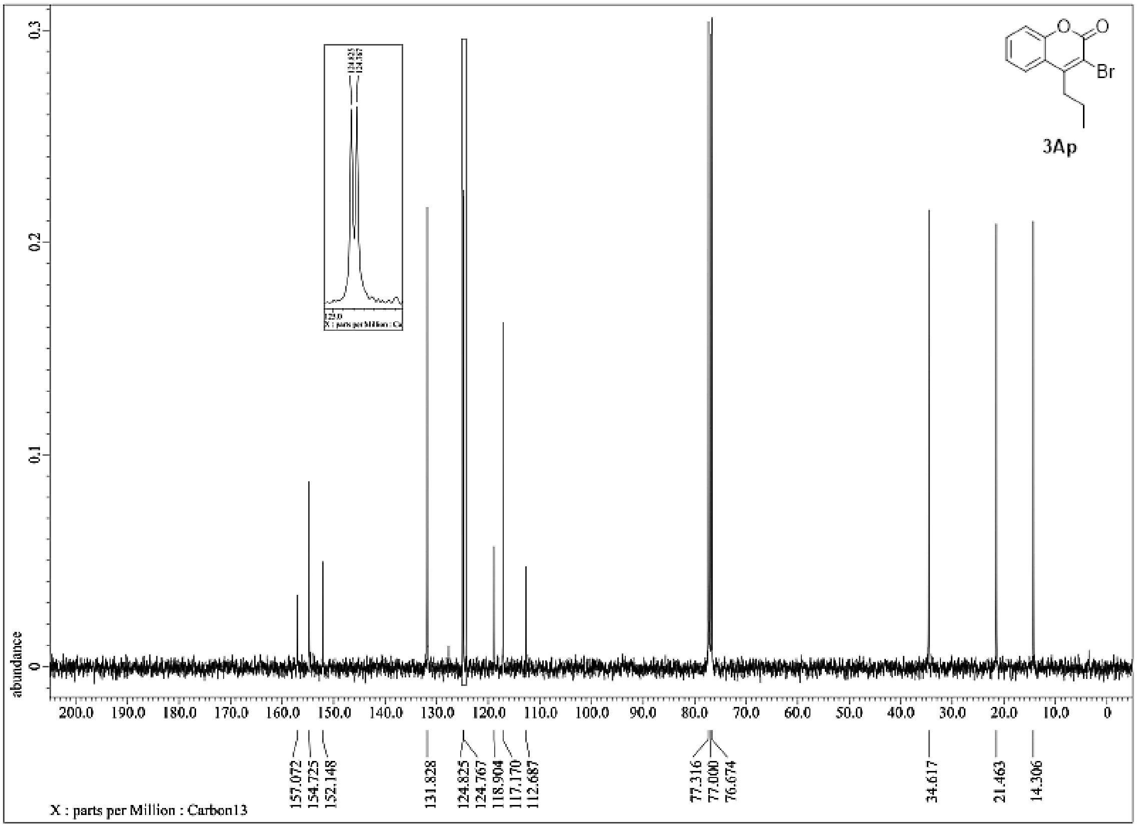
Br
3Ap
abundance
X : parts per Million : Carbon13
157.072
154.725
152.148
131.828
124.825
124.767
118.904
117.170
112.687
77.316
77.000
76.674
34.617
21.463
14.306

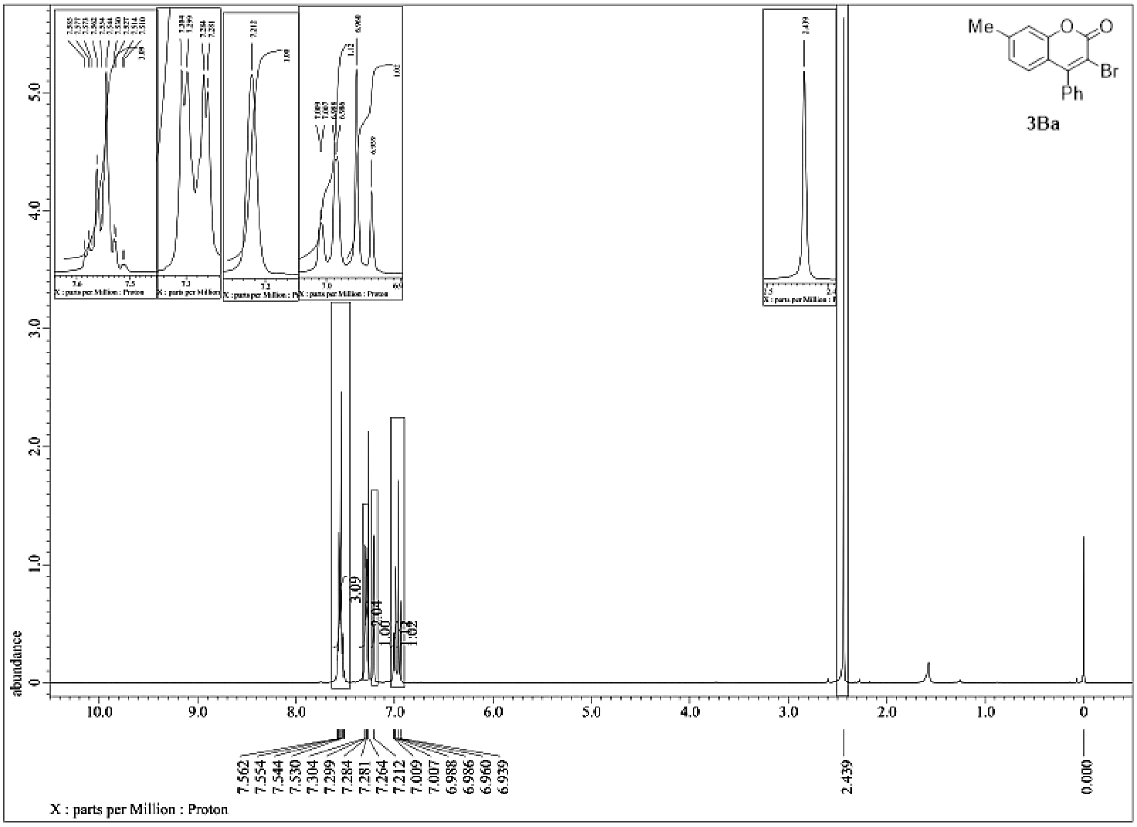
Me
O
O
Br
Ph
3Ba
abundance
X : parts per Million : Proton
3.09
2.04
1.00
1.12
1.02
7.562
7.554
7.544
7.530
7.304
7.299
7.284
7.281
7.264
7.212
7.009
7.007
6.988
6.986
6.960
6.939
2.439
0.000

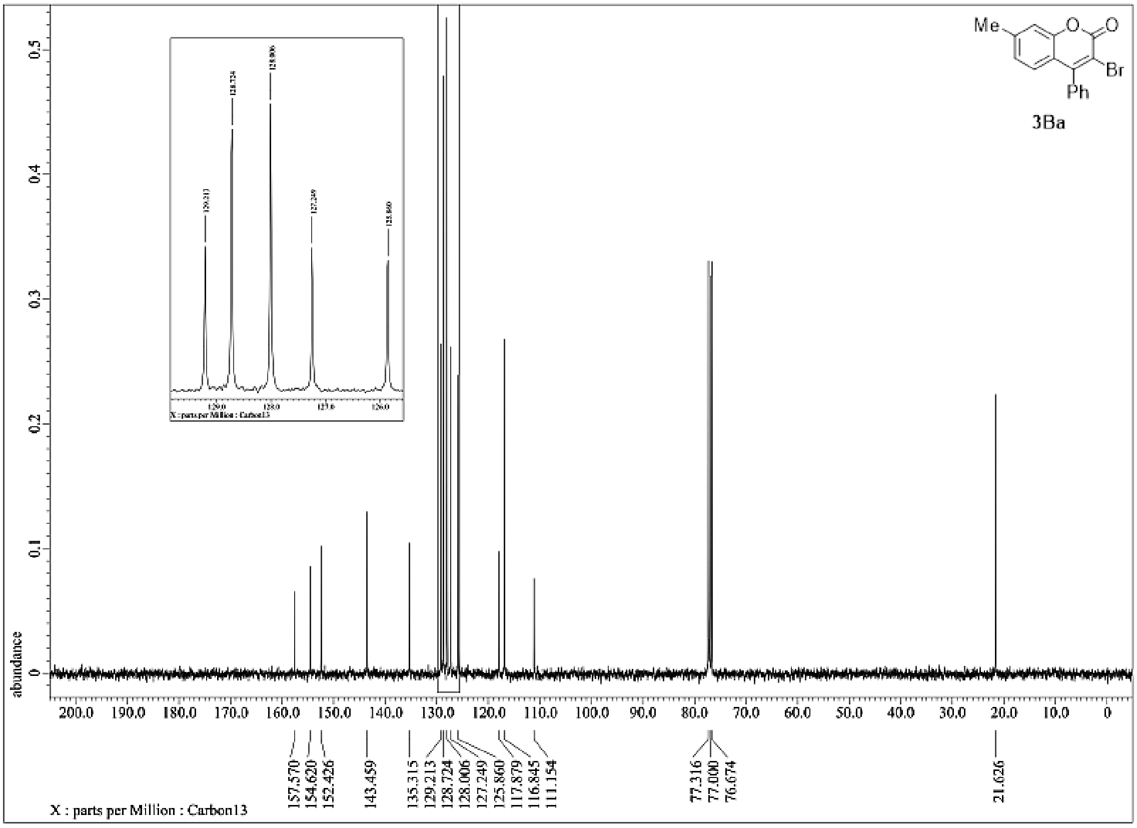
Me
O
O
Br
Ph
3Ba
abundance
X : parts per Million : Carbon13
157.570
154.620
152.426
143.459
135.315
129.213
128.724
128.006
127.249
125.860
117.879
116.845
111.154
77.316
77.000
76.674
21.026

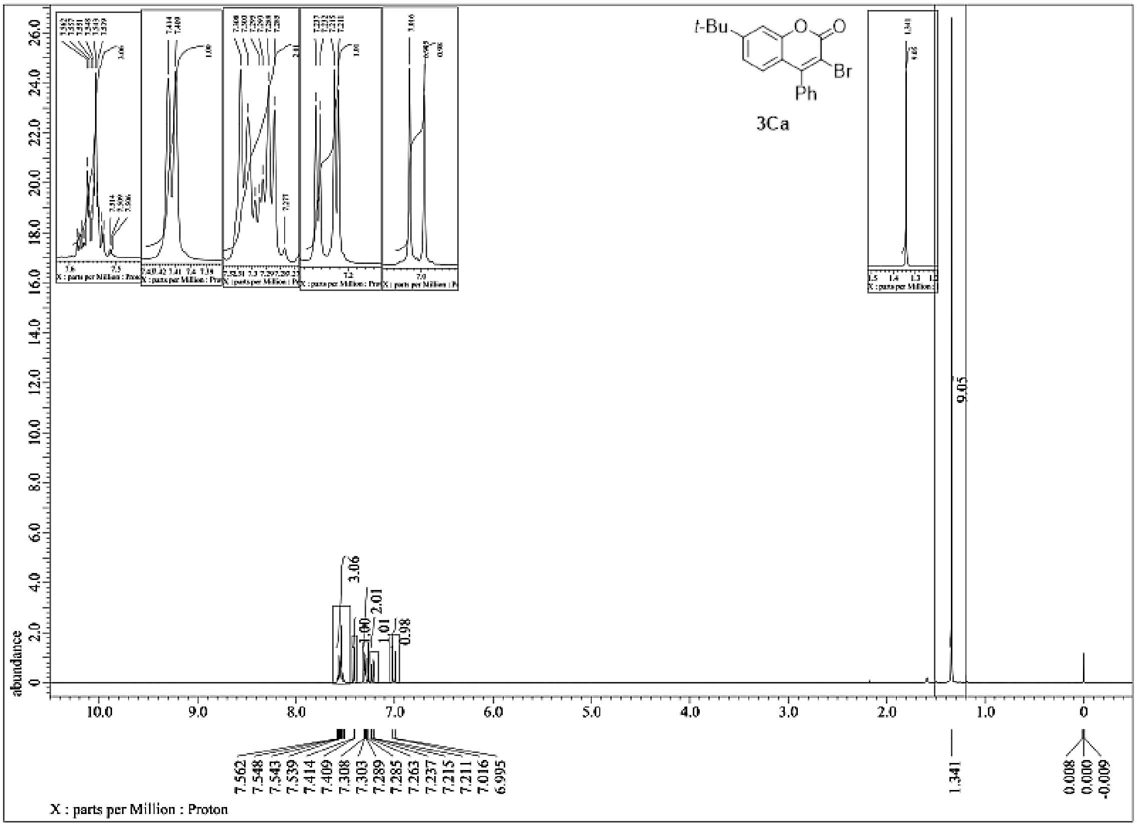
t-Bu
O
O
Br
Ph
3Ca
abundance
X : parts per Million : Proton
3.06
2.01
1.00
1.01
0.98
9.05
7.562
7.548
7.543
7.539
7.414
7.409
7.308
7.303
7.289
7.285
7.263
7.237
7.215
7.211
7.016
6.995
1.341
0.008
0.000
-0.009

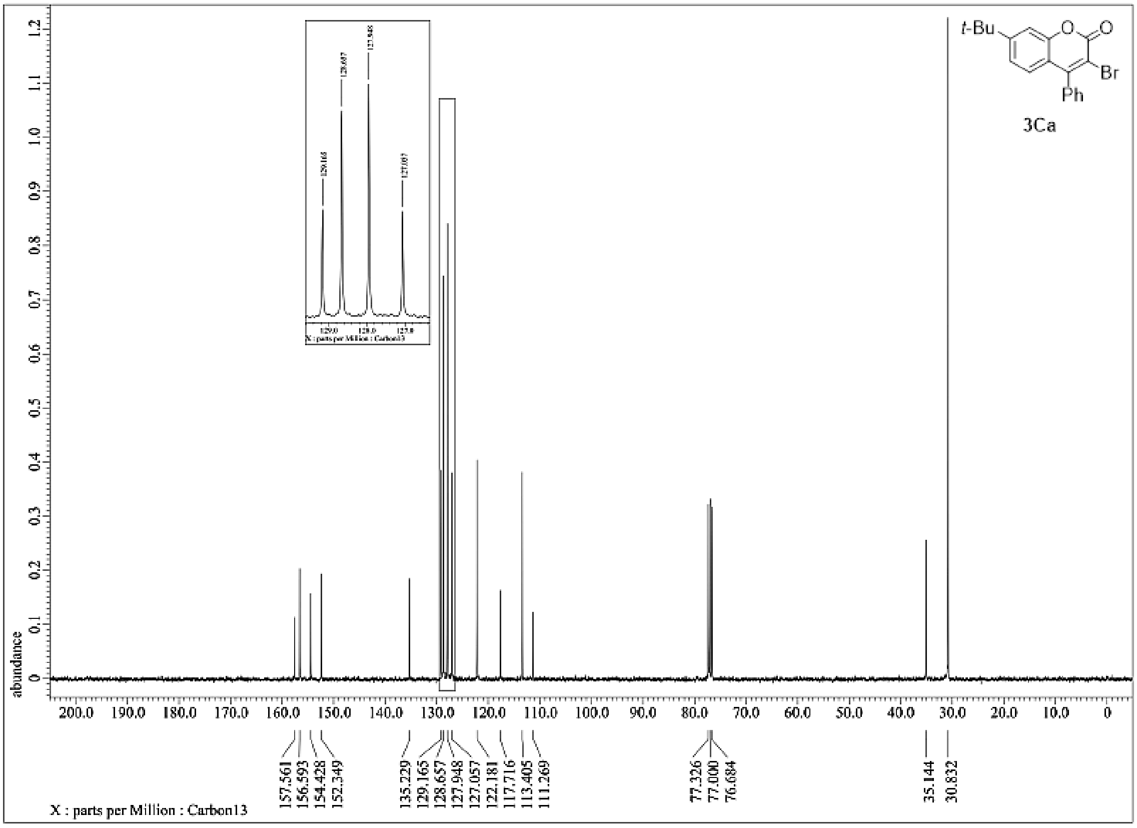
t-Bu
O
O
Br
Ph
3Ca
abundance
X : parts per Million : Carbon13
157.561
156.593
154.428
152.349
135.229
129.165
128.657
127.948
127.057
122.181
117.716
113.405
111.269
77.326
77.000
76.684
35.144
30.832

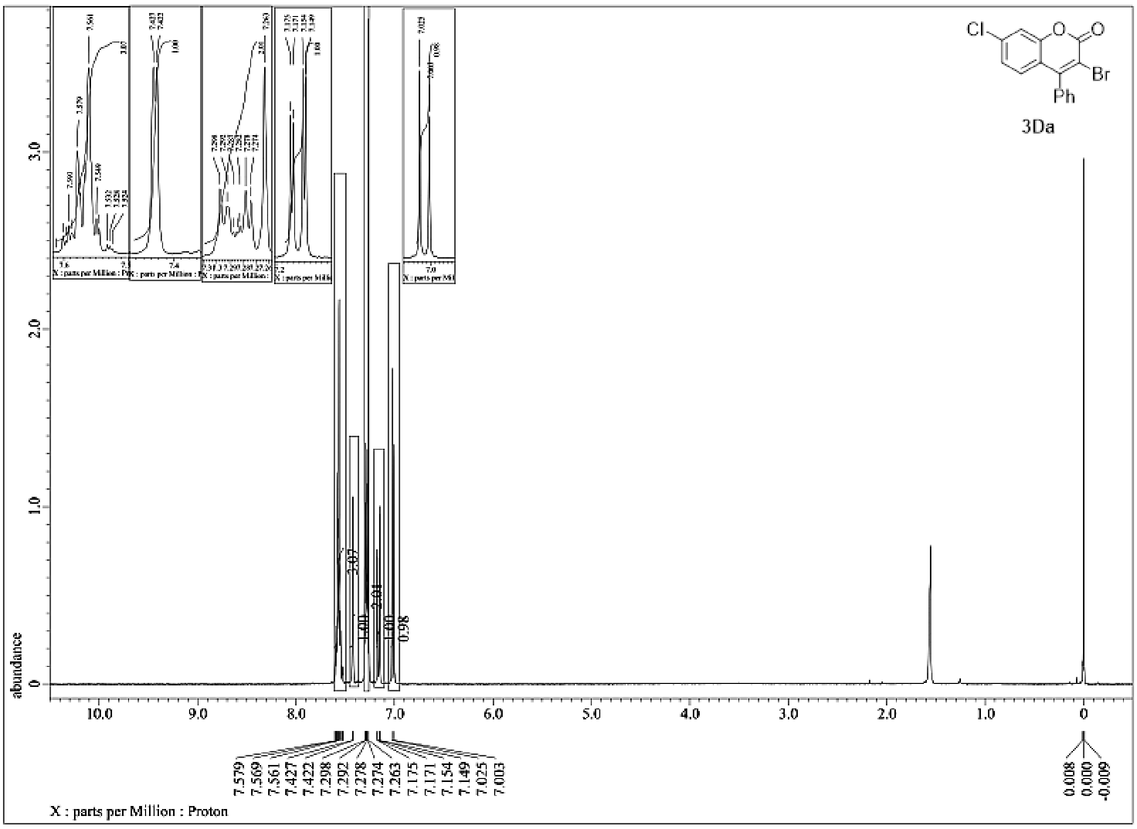
3Da
abundance
X : parts per Million : Proton
7.579
7.569
7.561
7.427
7.422
7.298
7.292
7.278
7.274
7.263
7.175
7.171
7.154
7.149
7.025
7.003
0.008
0.000
-0.009
2.07
1.01
2.01
1.00
0.98

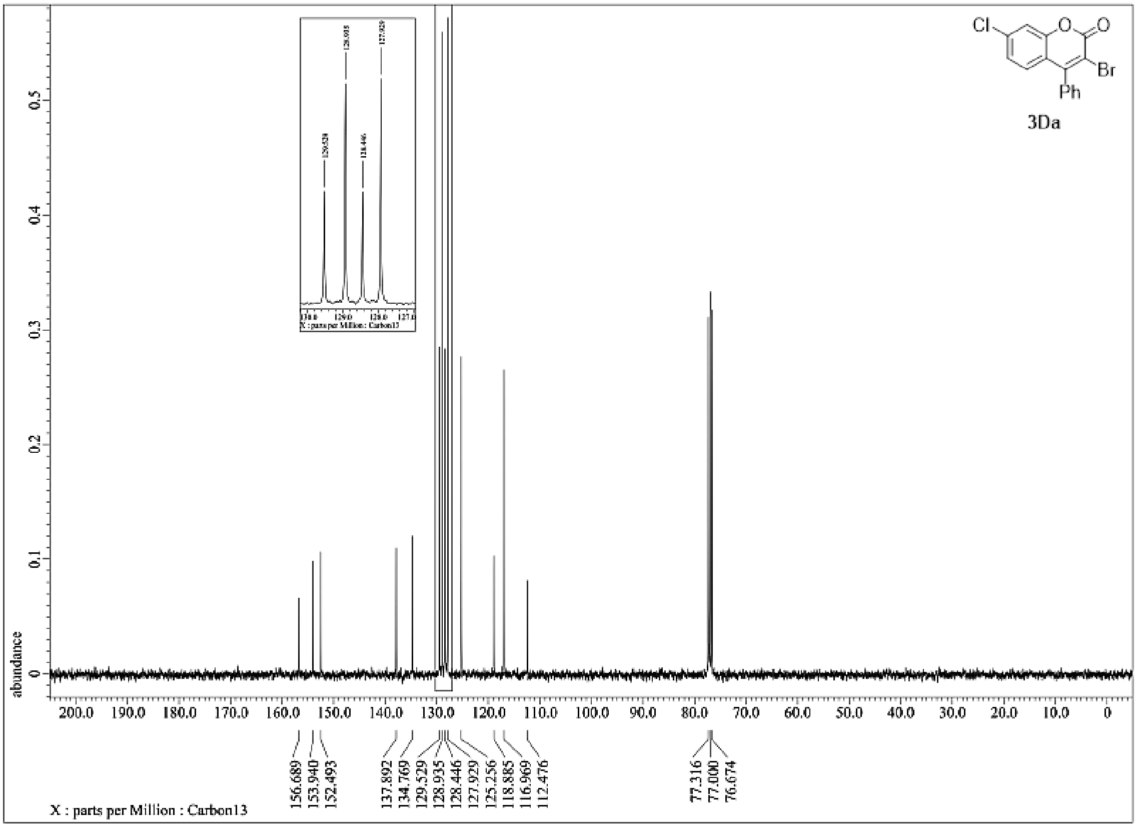
3Da
abundance
X : parts per Million : Carbon13
156.689
153.940
152.493
137.892
134.769
129.529
128.935
128.446
127.929
125.256
118.885
116.969
112.476
77.316
77.000
76.674

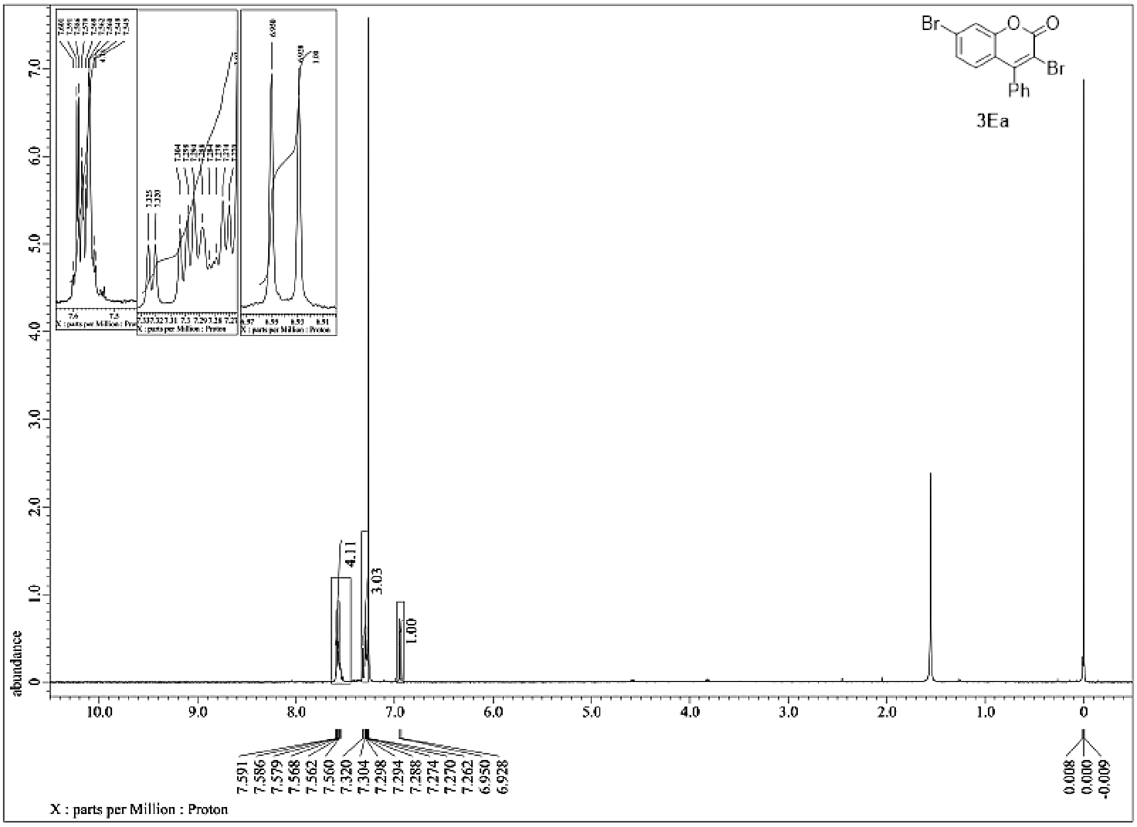
3Ea
abundance
X : parts per Million : Proton
7.591
7.586
7.579
7.568
7.562
7.560
7.320
7.304
7.298
7.294
7.288
7.274
7.270
7.262
6.950
6.928
0.008
0.000
-0.009
4.11
3.03
1.00

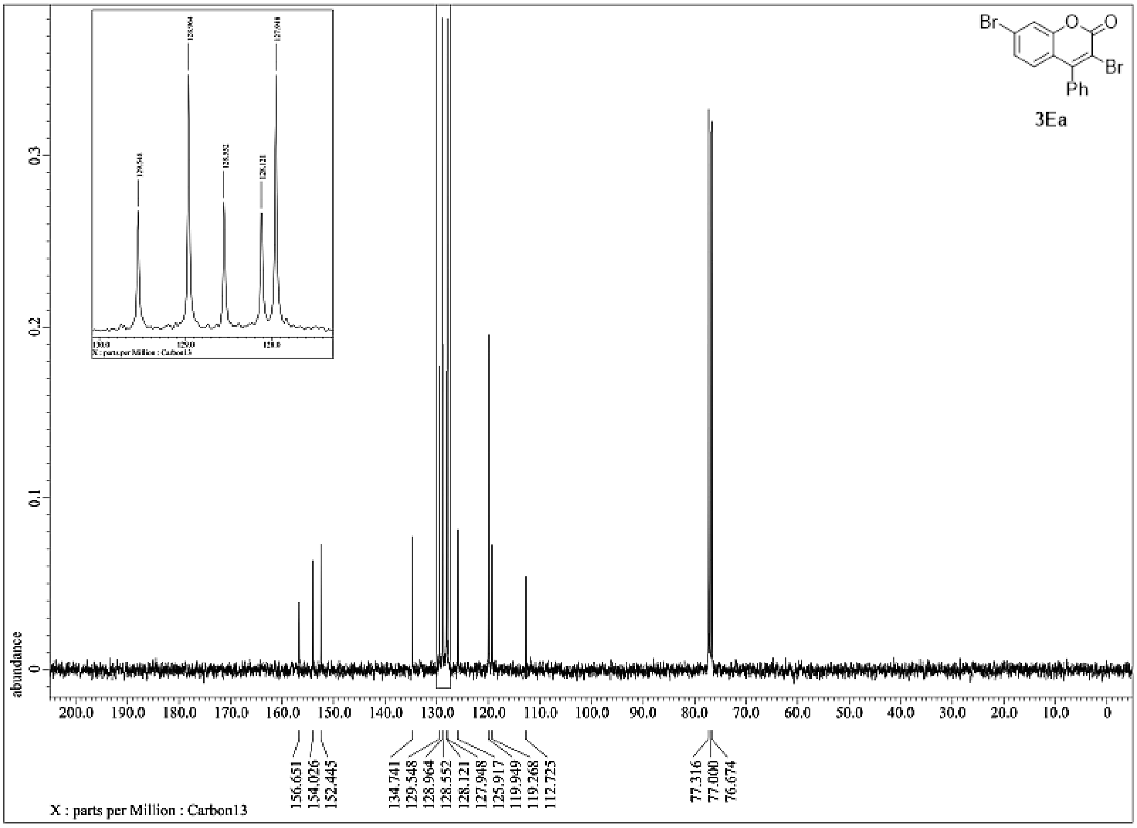
3Ea
abundance
X : parts per Million : Carbon13
156.651
154.026
152.445
134.741
129.548
128.964
128.552
128.121
127.948
125.917
119.949
119.268
112.725
77.316
77.000
76.674

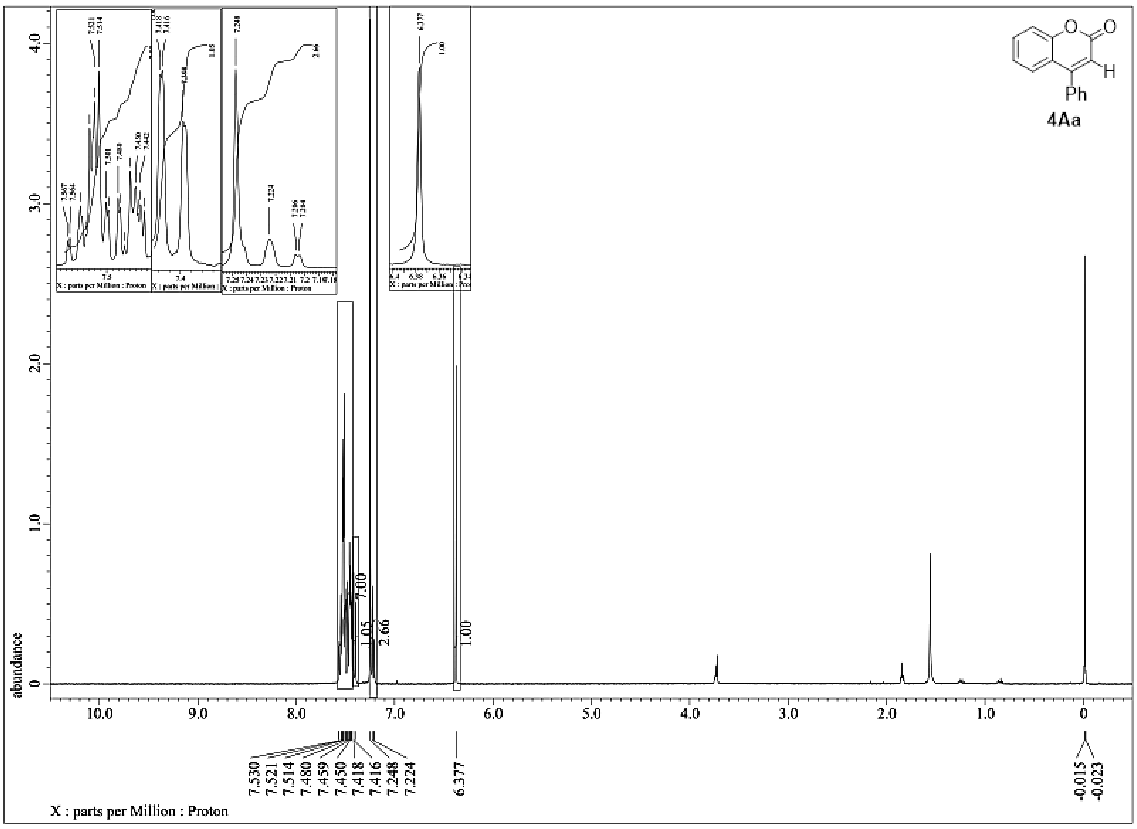

4Aa
abundance
X : parts per Million : Proton

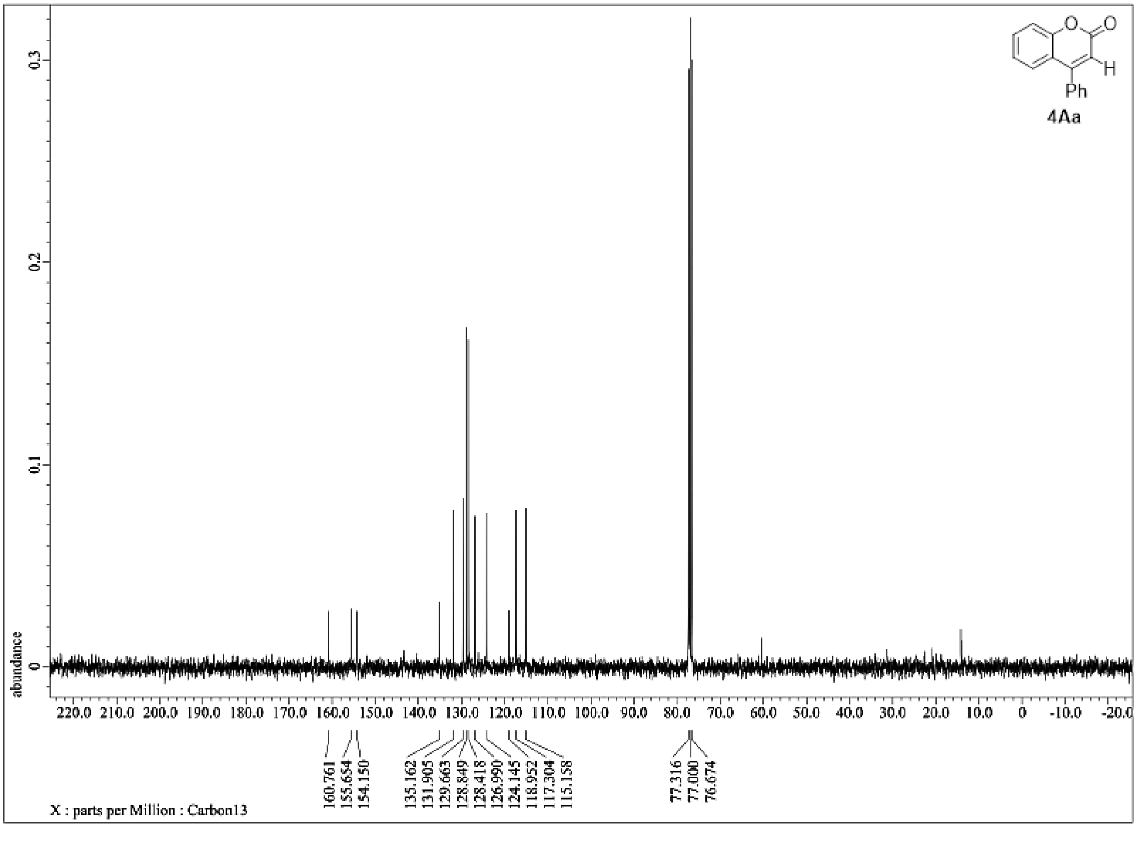

4Aa
abundance
X : parts per Million : Carbon13

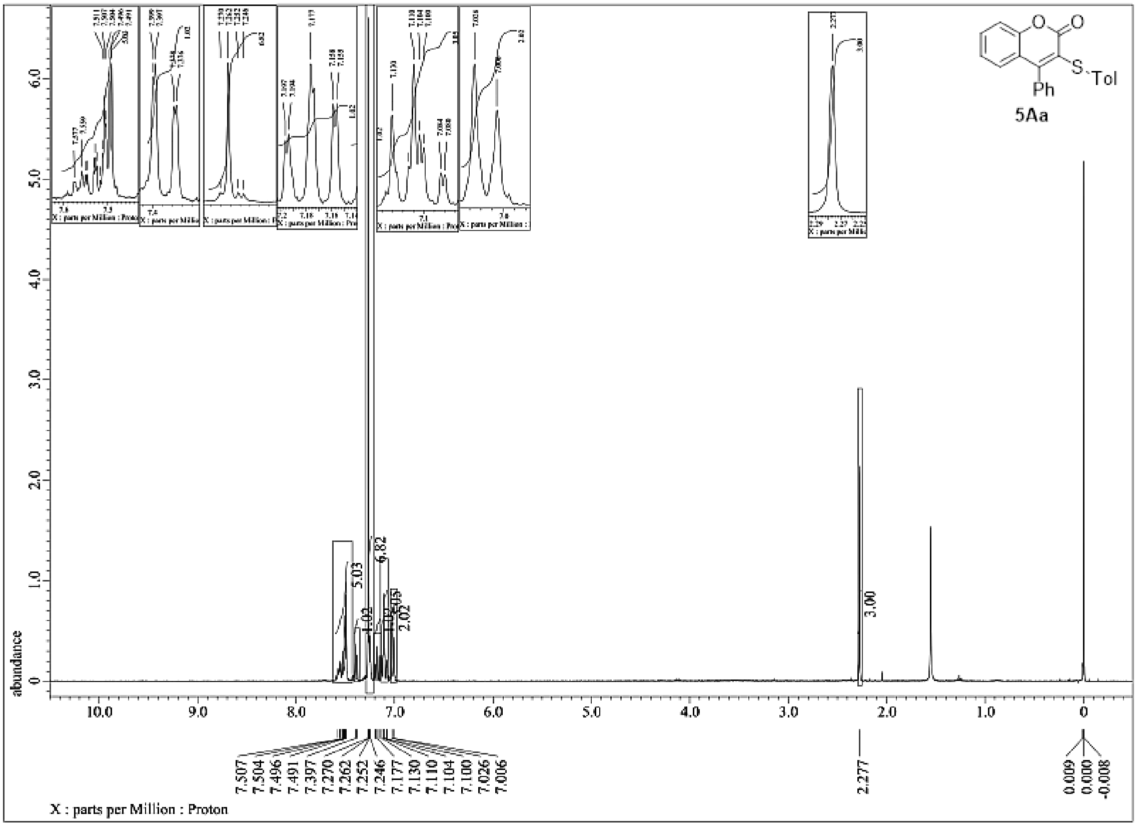
5Aa
X : parts per Million : Proton
abundance

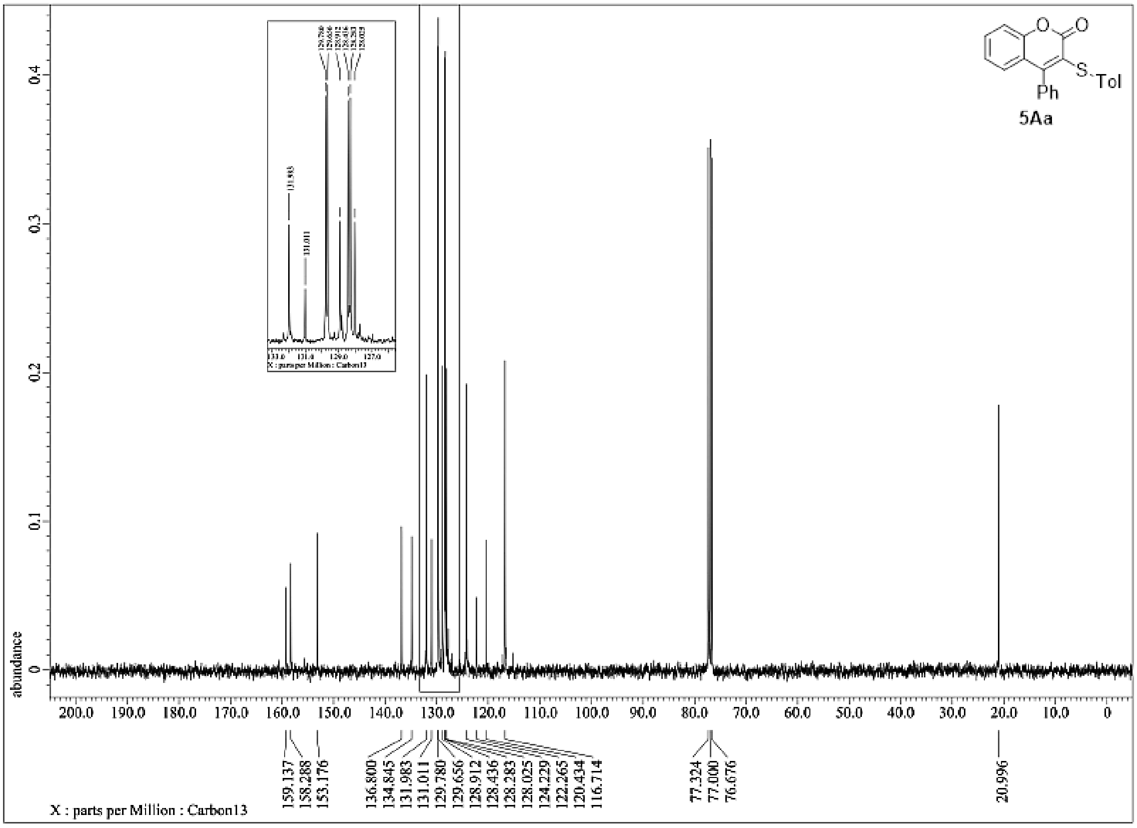
5Aa
X : parts per Million : Carbon13
abundance

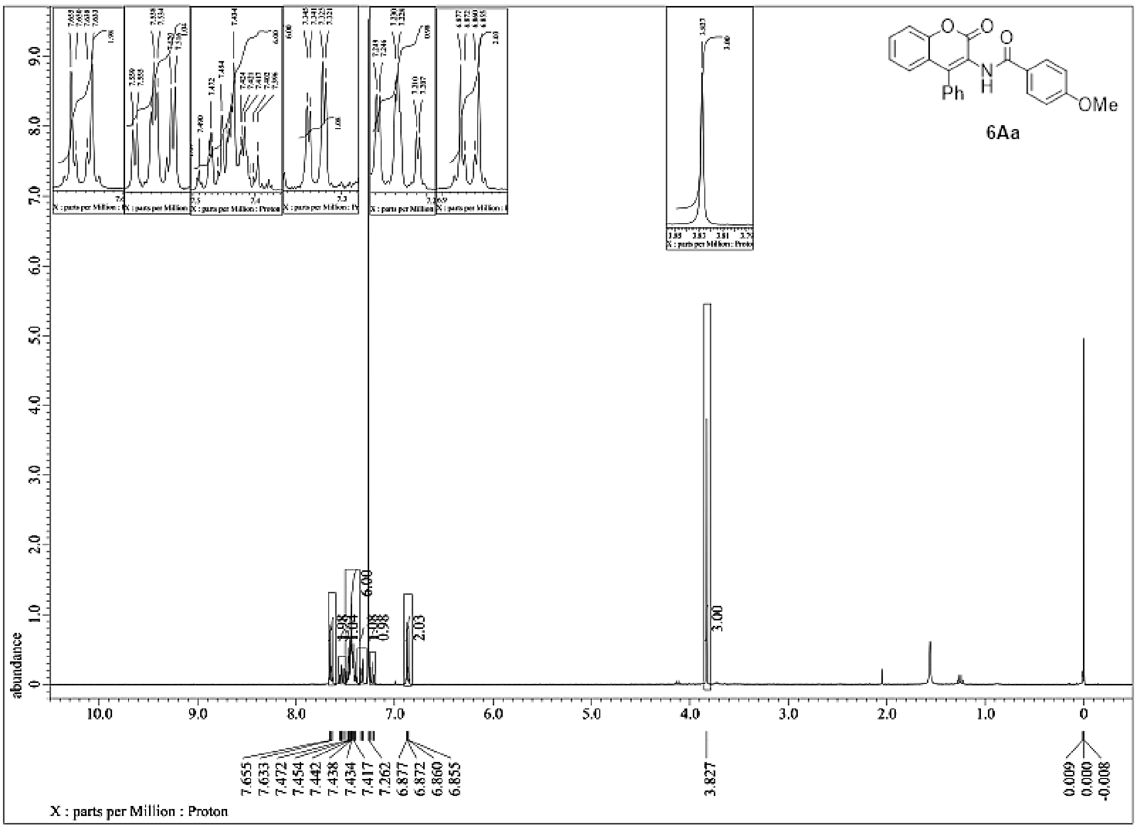
Ph
H
N
O
O
O
OMe
6Aa
abundance
X : parts per Million : Proton
7.655
7.633
7.472
7.454
7.442
7.438
7.434
7.417
7.262
6.877
6.872
6.860
6.855
3.827
0.009
0.000
-0.008
6.00
3.00
2.03

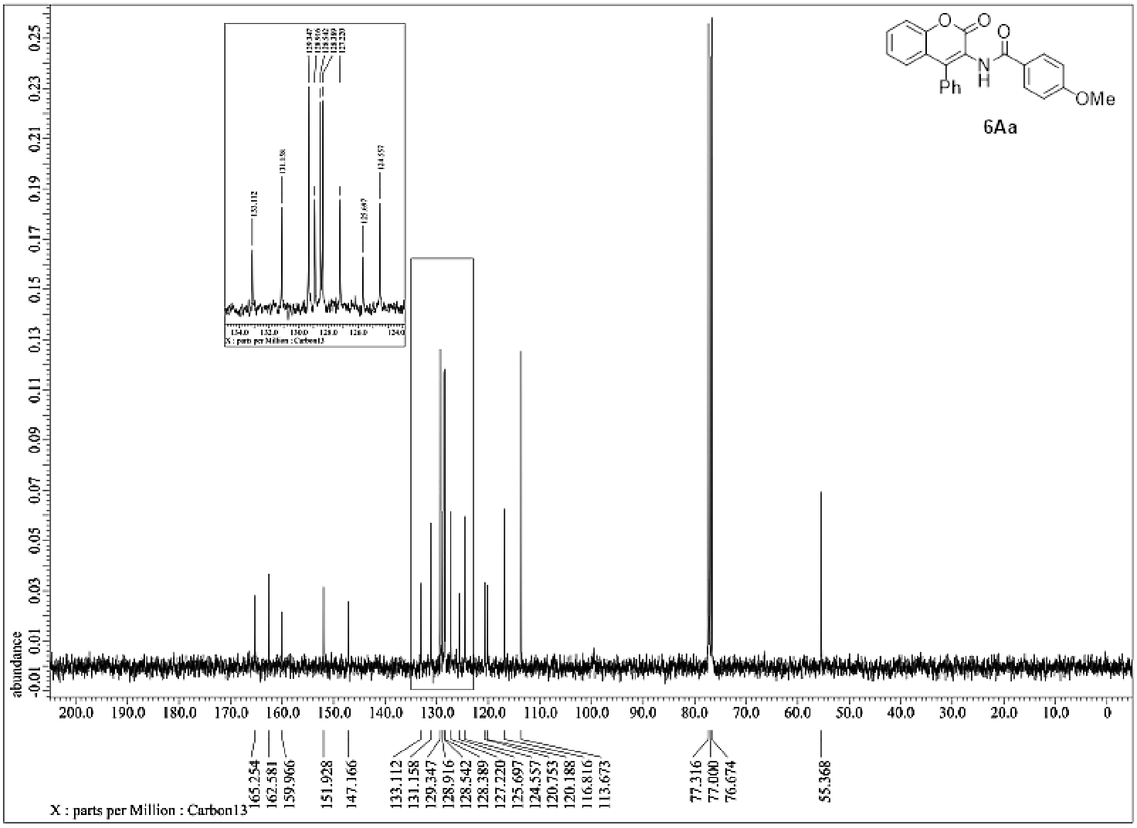
Ph
H
N
O
O
O
OMe
6Aa
abundance
X : parts per Million : Carbon13
165.254
162.581
159.966
151.928
147.166
133.112
131.158
129.347
128.916
128.542
128.389
127.220
125.697
124.557
120.753
120.188
116.816
113.673
77.316
77.000
76.674
55.368

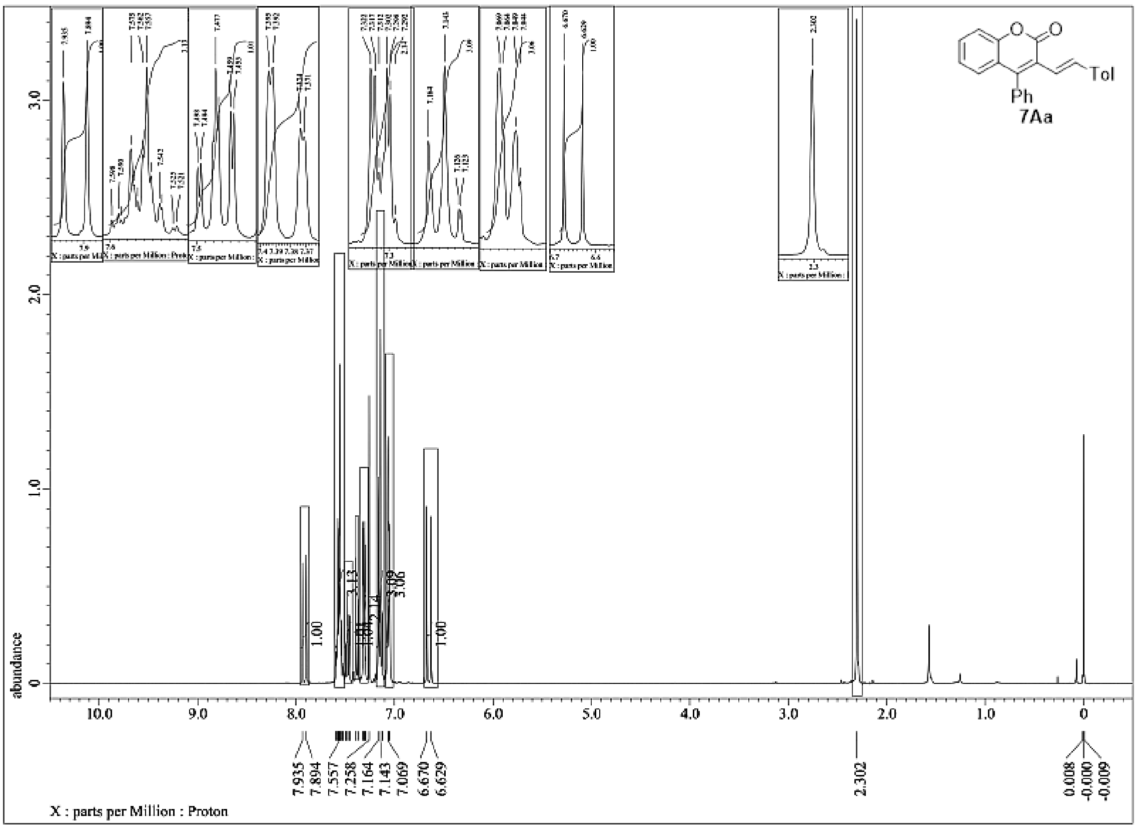
abundance
Ph
7Aa
Tol
7.935
7.894
7.557
7.258
7.164
7.143
7.069
6.670
6.629
2.302
0.008
-0.000
-0.009
X : parts per Million : Proton

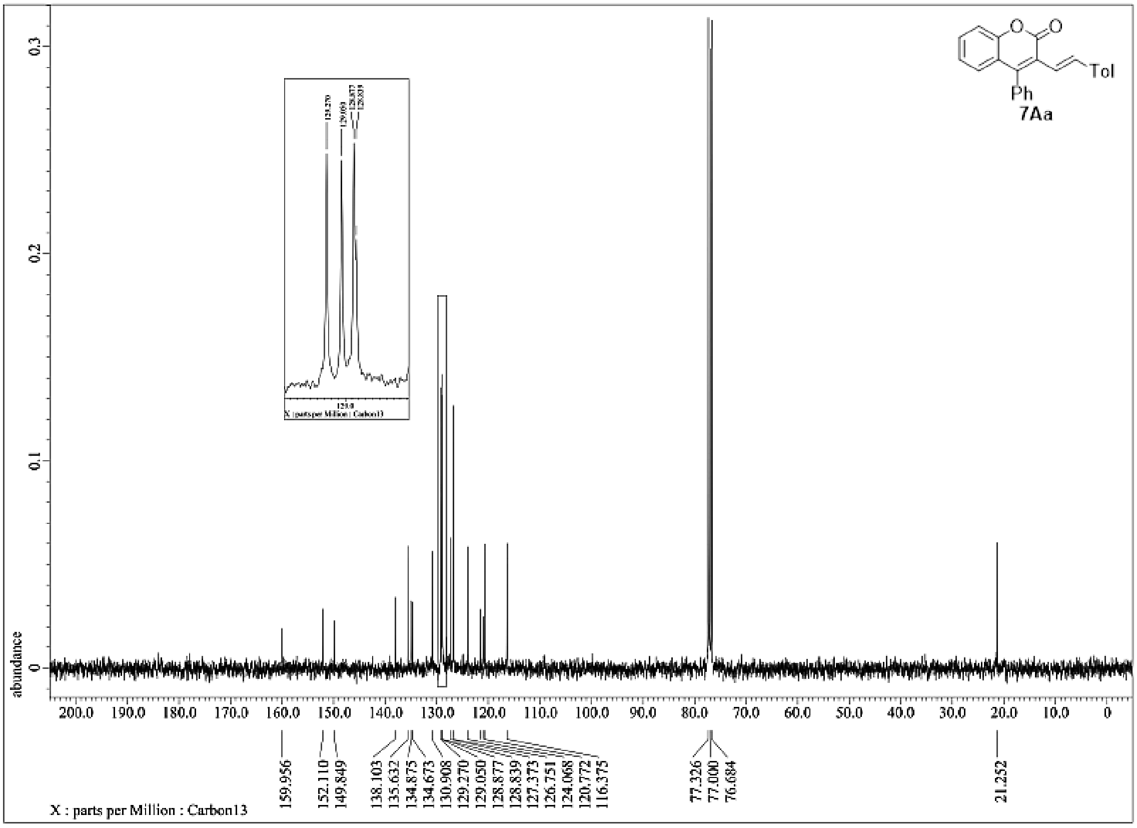
abundance
Ph
7Aa
Tol
159.956
152.110
149.849
138.103
135.632
134.875
134.673
130.908
129.270
129.050
128.877
128.839
127.373
126.751
124.068
120.772
116.375
77.326
77.000
76.684
21.252
X : parts per Million : Carbon13

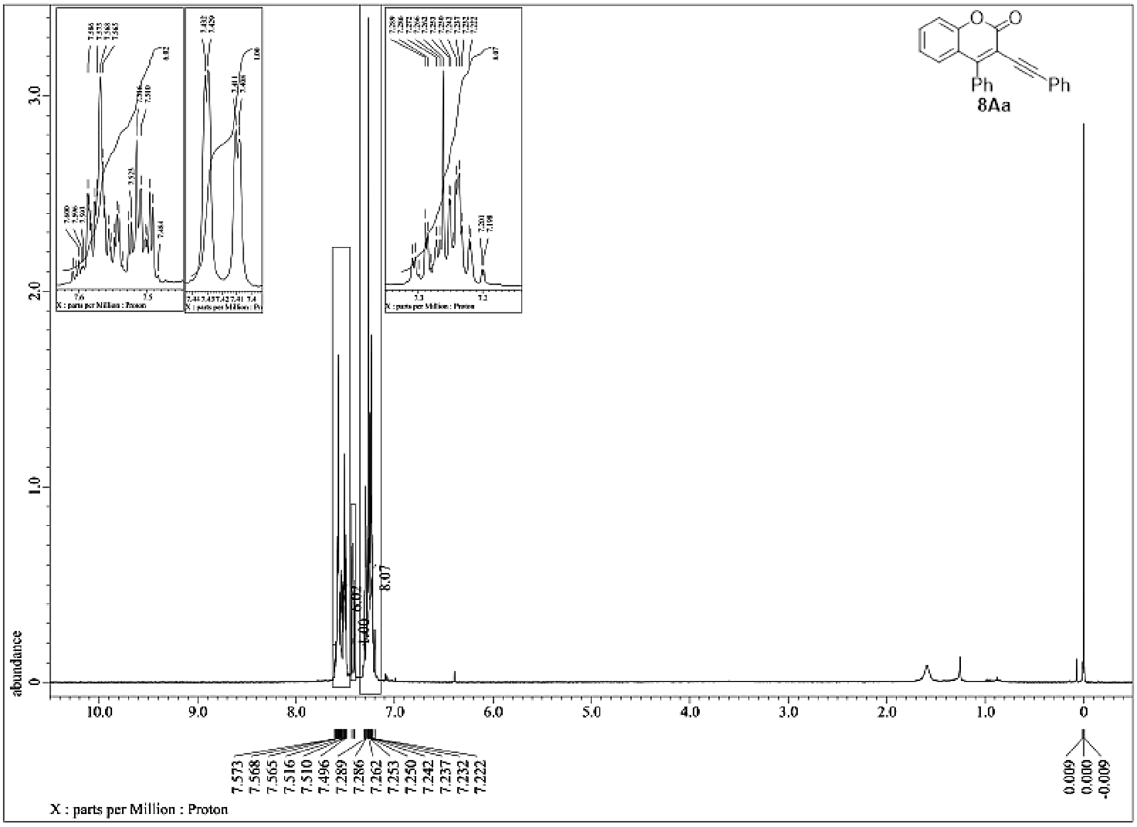
8Aa
Ph
Ph
abundance
X : parts per Million : Proton
7.573
7.568
7.565
7.516
7.510
7.496
7.289
7.286
7.262
7.253
7.250
7.242
7.237
7.232
7.222
0.009
0.000
-0.009
6.07
1.00
8.07

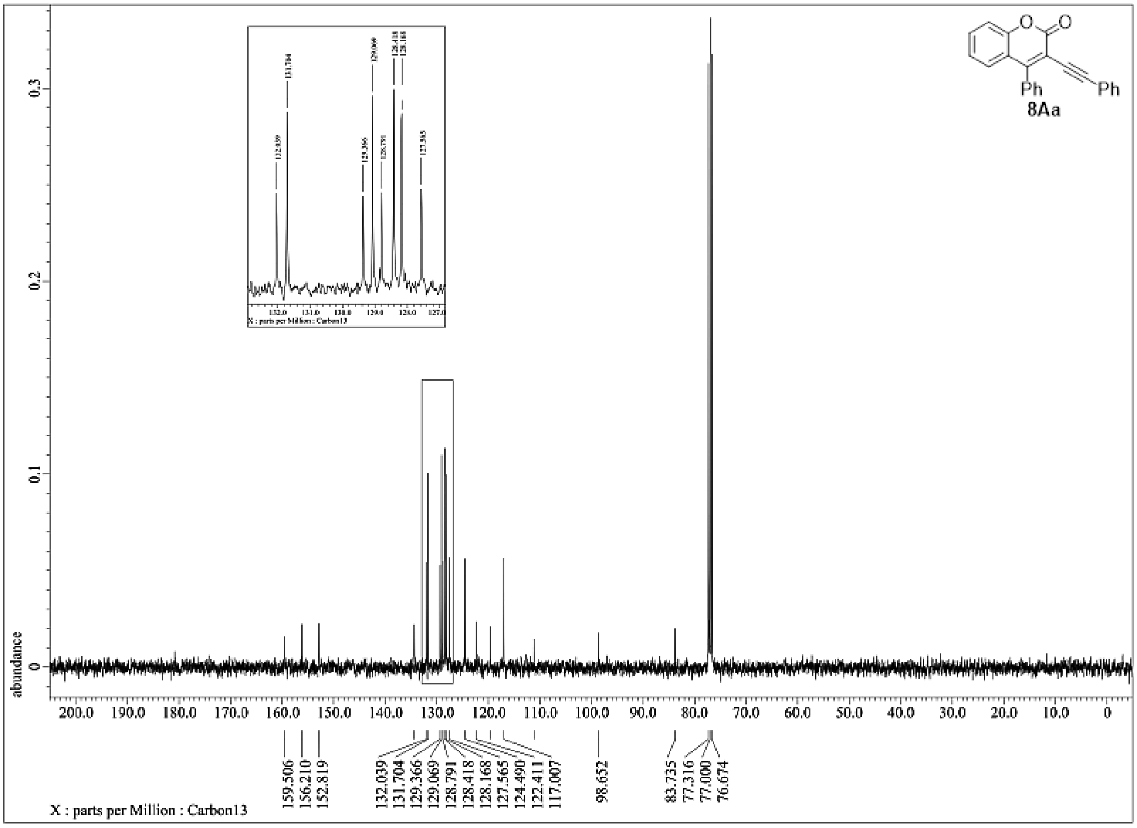
8Aa
Ph
Ph
abundance
X : parts per Million : Carbon13
159.506
156.210
152.819
132.039
131.704
129.366
129.069
128.791
128.418
128.168
127.565
124.490
122.411
117.007
98.652
83.735
77.316
77.000
76.674

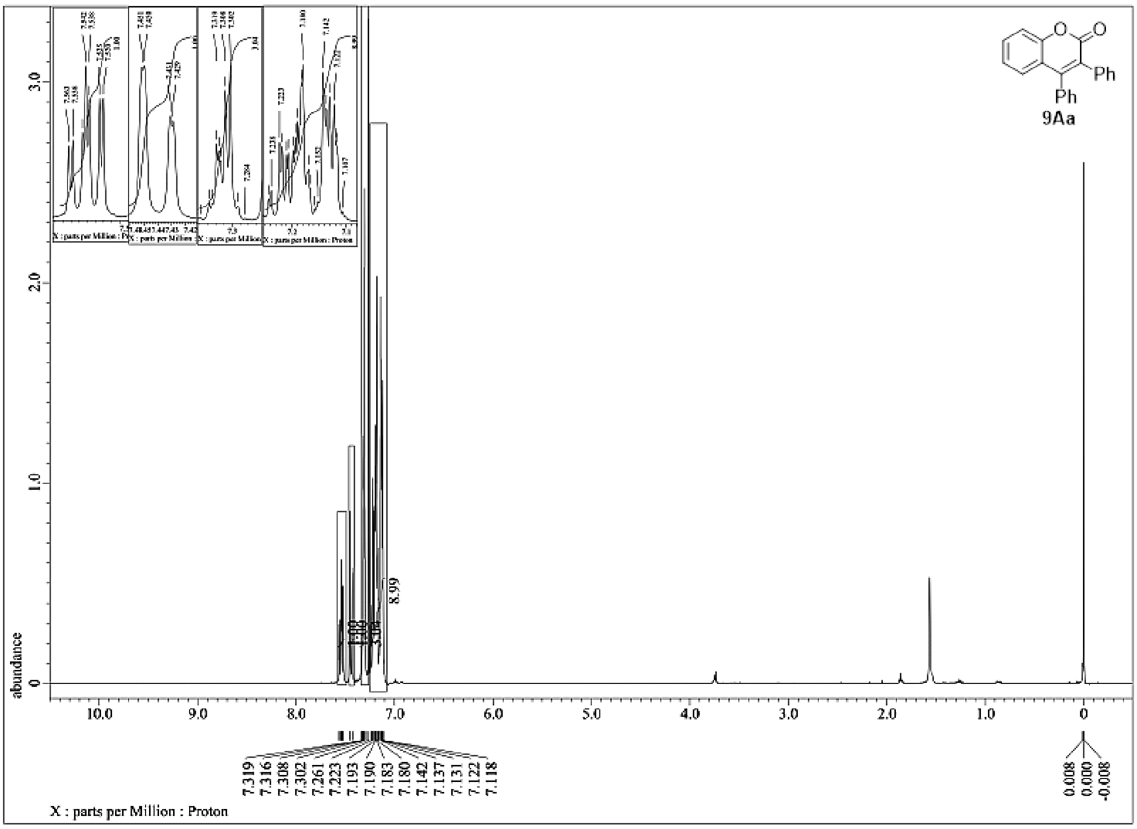
Ph
Ph
9Aa
abundance
X : parts per Million : Proton
7.319
7.316
7.308
7.302
7.261
7.223
7.193
7.190
7.183
7.180
7.142
7.137
7.131
7.122
7.118
0.008
0.000
-0.008
8.99

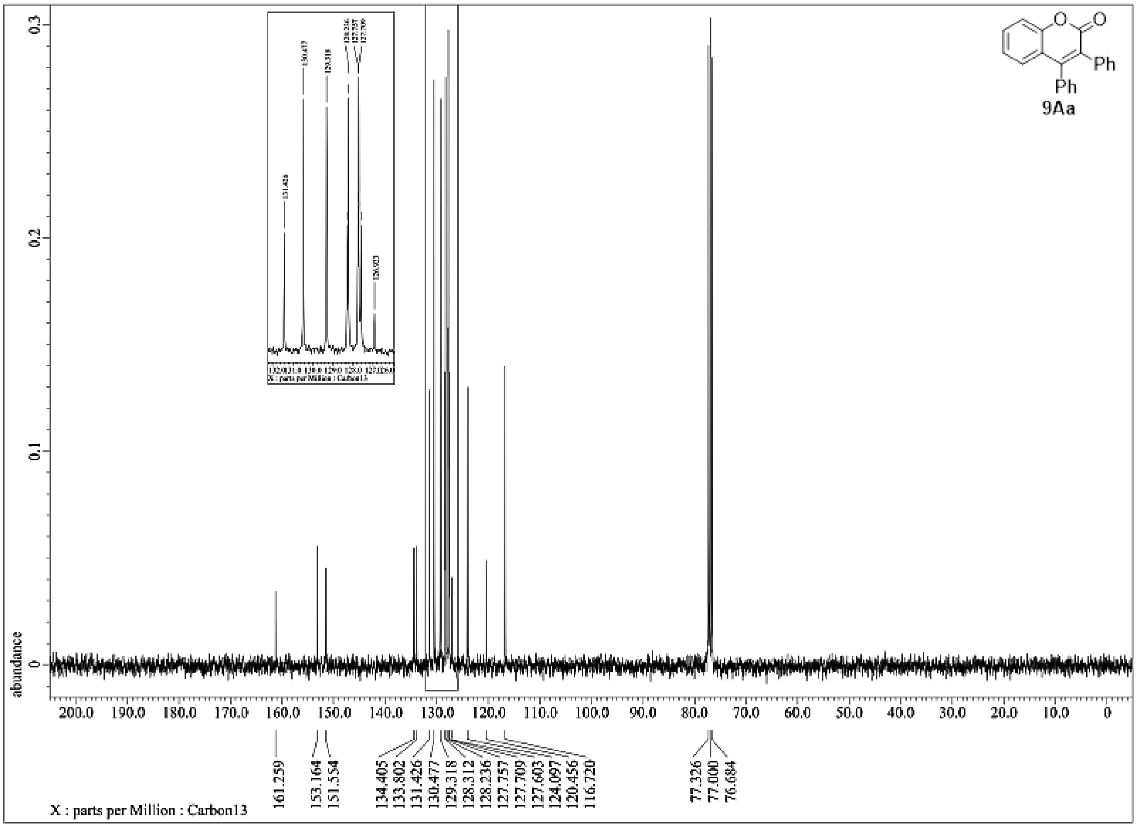
Ph
Ph
9Aa
abundance
X : parts per Million : Carbon13
161.259
153.164
151.554
134.405
133.802
131.426
130.477
129.318
128.312
128.236
127.757
127.709
127.603
124.097
120.456
116.720
77.326
77.000
76.684
